# Gold(I)-catalyzed alkene hydroamination: revelations from kinetic benchmarking

**DOI:** 10.3762/bjoc.22.129

**Published:** 2026-11-06

**Authors:** Jaden McCane, Yujing Qu, Ruichen Lan, Amanda C Jones

**Affiliations:** 1 Chemistry, Wake Forest University, 1834 Gulley Rd., Winston-Salem, NC, 27109, USA

**Keywords:** acid catalysis, alkene hydroamination, gold catalysis, phosphine ligand effect

## Abstract

Kinetic and reactivity studies on the intramolecular hydroamination of 1-allyl-1,3-diphenylurea and related analogues, as well as protected variants of 2-(allyloxy)aniline using both gold(I) and acid catalysts revealed gold catalyst resting states and deactivation pathways. The structure of the phosphine ligand and counterion on gold impacted the extent to which alkylgold intermediates could be detected, and, at higher temperatures, the rate of reaction with 1,2-dichloroethane (DCE) solvent to generate inactive LAuCl and Brønsted acid by-product. Comparisons to the reactivity of urea, carbamate, thiourea, and thiocarbamate showed that failure of gold to enable catalytic hydroamination does not correlate with failure to activate alkenes toward nucleophilic addition to form alkylgold intermediates. Furthermore, the presence of electron-withdrawing groups on the phosphine ligand can lead to increases in reactivity, despite greater amounts of deactivated complex. Acid catalysts are shown to react markedly different from gold catalysts, and in one case a cooperative effect was identified whereby a gold/acid mixture led to a faster rate by perturbing alkylgold intermediate concentration. The impact of counterion was not consistent across all substrates, suggesting a complex role.

## Introduction

Activation of alkenes by gold(I) catalysts has been a subject of dynamic debate since He’s 2006 report using sulfonamides and Ph_3_AuOTf [1]. This was followed by suspicions of “hidden Brønsted acid catalysis,” and kinetic and selectivity observations indistinguishable from use of HOTf alone [2–3]. We have remained interested in quantifying structure–activity relationships for gold(I) catalysts and have found the mechanistic features to be complicated. For example, the necessity of gold in many hydroaminations is supported by control studies where acid alone is shown ineffective, however, the observed behavior of alkylgold intermediates does not fully support a classic “π-activation protodeauration” pathway [4]. With this in mind, we continue to examine the explicit effects of substrate and catalyst identity on effective reactivity patterns. During recent studies we found two alkene substrates that revealed distinct pathways for catalyst deactivation that complicate easy conclusions about catalyst efficacy if only yield and reaction time are assessed. Instead, analysis of reaction intermediates and catalyst identity aid interpretation of results for future catalyst optimization.

## Results and Discussion

### Revelation 1: alkylgold observed in situ

**Scheme 1:**
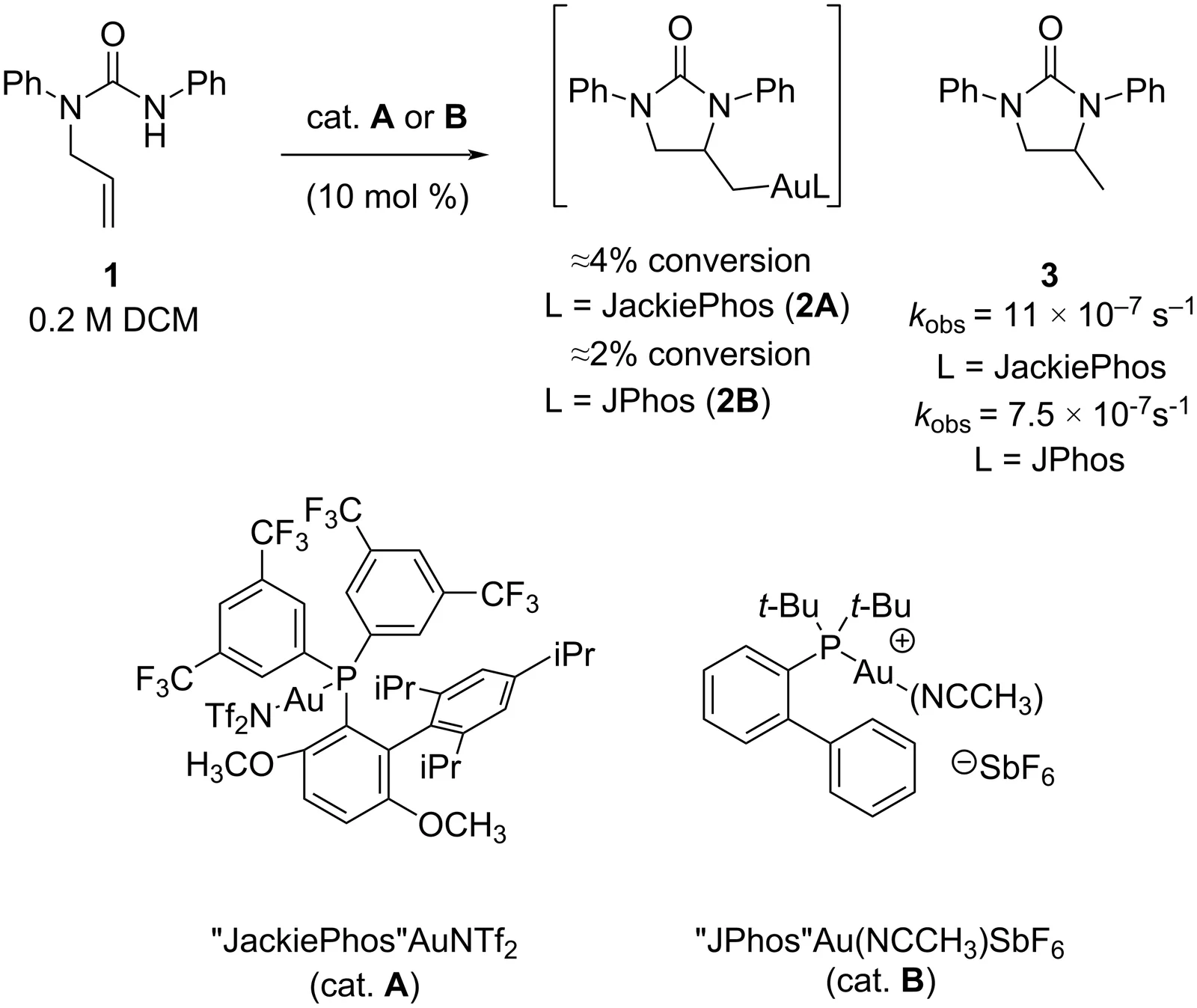
Different concentrations of alkylgold **2** observed in situ during hydroamination of 1-allyl-1,3-diphenylurea (**1**) with gold(I) catalysts (as monitored by ^1^H NMR spectroscopy, *k*_obs_ average of two trials).

In the context of other studies, we were examining the intramolecular hydroamination of urea **1** with gold catalysts to form **3**, a reaction first reported by Widenhoefer [5]. When **1** was treated with 10 mol % JackiePhosAuNTf_2_ (JackiePhos = bis(3,5-bis(trifluoromethyl)phenyl)(2′,4′,6′- triisopropyl-3,6-dimethoxybiphenyl-2-yl)phosphine, cat. **A**) or [JPhosAu(CH_3_CN)]SbF_6_ (JPhos = [1,1'-biphenyl]-2-yldi-*tert*-butylphosphane, cat. **B**) and monitored over time by ^1^H NMR spectroscopy, a ligand and/or anion-driven subtlety was identified (Scheme 1). Unexpectedly, alkylgold **2** was observed in situ as the reaction progressed as identified by diagnostic diastereotopic hydrogens alpha to gold and 3-bond ^31^P–^1^H coupling across gold (as first reported by Toste and co-workers [6], see for example Figure S23 in Supporting Information File 1). Alkylgold intermediates have been strategically intercepted by introduction of exogenous base [6–7]; or prepared independently using transmetalation [8]. This was the first time we observed alkylgold under traditional conditions. The overall rate of hydroamination was quite similar for both catalysts (*k*_rel_ = 1.5, **A**/**B**), while the relative amounts of alkylgold were significantly different. Catalyst **A** formed alkylgold at a 20-fold increase compared to catalyst **B**. A catalyst screen may have led to the conclusion that catalyst identity was not a significant driver of reaction efficiency and this presents an unanticipated opportunity to further probe the role of alkylgold in the mechanism of hydroamination.

### Revelation 2: degradation behavior

**Scheme 2:**
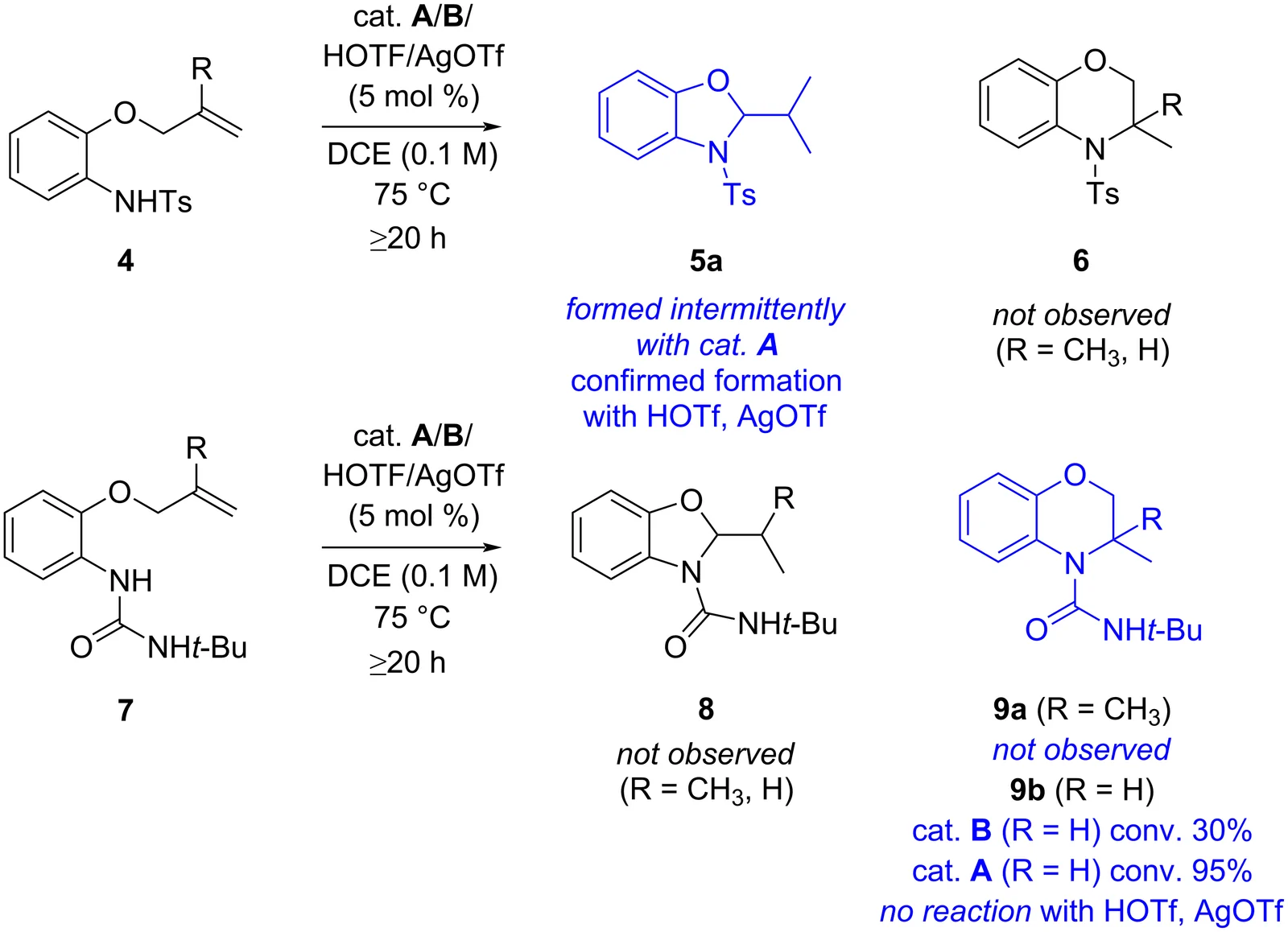
Initial survey of reactivity to compare gold(I) catalytic activity to that of AgOTf and HOTf in hydroamination and tandem isomerization/hydroamination.

In a separate study, we were examining the efficiency of gold(I) catalysts for the hydroamination of protected aminophenols such as sulfonamide **4** and urea **7** (Scheme 2). We were inspired by the work of Youn and co-workers, wherein AgOTf was shown to accomplish olefin isomerization/intramolecular hydroamination to generate heterocycle **5a** from **4a** (R = CH_3_) [9]. Control studies implicated HOTf generated in situ as the active catalyst, either through H_2_O hydrolysis, or the interaction of silver with 1,2-dichloroethane solvent or the substrate NH. Considering the uncertain role of hidden Brønsted acid catalysis in gold(I)-catalyzed reactions, we envisioned this as a potential probe, while also seeking to calibrate reactivity differences between substrate structure and catalyst identity. We performed an initial screen with 5 mol % cat. **A** and **B** at 75 °C in 1,2-dichloroethane (DCE), while also verifying the literature-reported reactivity with AgOTf and HOTf. Tandem isomerization/hydroamination was effective for **4a** (R = CH_3_), however, unlike AgOTf and HOTf, gold(I) catalysts were intermittently effective, with success only ever observed with cat. **A** (see Table S1, for further details on conditions screened, in Supporting Information File 1). The sulfonamide protecting group and methylallyl are both critical features for isomerization, as also seen in Youn’s original work. Direct hydroamination was only ever effective for substrate **7b** (R = H) when gold(I) catalysts were used, but ineffective with HOTf or AgOTf. What was more puzzling under gold catalytic conditions, however, was that when reaction mixtures were analyzed qualitatively by ^31^P NMR spectroscopy, both showed the appearance of LAuCl but with relatively different amounts of remaining active catalyst (see Supporting Information File 1, Figures S17 and S18). With cat. **B**, 30% conversion for hydroamination was observed after 21 hours, while cat. **A** gave 95% conversion in a similar amount of time (*t* = 24 hours). Unexpectedly, cat. **B** was predominately intact with minor amounts of LAuCl while cat. **A** was no longer detectable and showed a significant amount of LAuCl at reaction end. The more effective catalyst showed the greatest amount of degradation.

Assuming generation of LAuCl coincided with formation of HX, (where X is weakly coordinating), JackiePhosAuOTf was used during subsequent control studies for a more accurate comparison to the known reactivity of HOTf [9]. To check whether the by-products of degradation were themselves active, JackiePhosAuOTf was heated to 75 °C in DCE for 24 hours. We confirmed by ^31^P NMR spectroscopy the replacement of the signal for JackiePhosAuOTf by the signal for JackiePhosAuCl within 3 hours at 75 °C (Figure S20, Supporting Information File 1). Separately, substrates **4a** (R = CH_3_) and **7b** (R = H) were then added and the mixtures were heated again for 24 hours. Marginal isomerization/hydroaminaton was observed for sulfonamide **4a**, and no hydroamination was observed for urea **7b**. These results have two important implications – first, JackiePhosAuOTf is a poor source of acid, and second, activity for hydroamination to **9b** persists despite significant conversion to inactive LAuCl (independently prepared JackiePhosAuCl was confirmed to be ureactive).

JPhosAuNTf_2_, IPrAuNTf_2_ (IPr = 1,3-bis(2,6-diisopropylphenyl)imidazole-2-ylidene), and Ph_3_PAuNTf_2_ were also tested. Amongst those only the latter was a competent source of acid, as treatment of **4a** with 10 mol % Ph_3_PAuNTf_2_ in DCE led to 35% conversion to **5a** with concomitant formation of [(Ph_3_P)_2_Au]NTf_2_. Therefore, gold(I) catalyst degradation with acid release is both ligand and anion sensitive, and observation of LAuCl is not itself an indicator of acid generation. In the case of Ph_3_PAuNTf_2_, the absence of Ph_3_PAuCl suggests an alternate mechanism for acid release that does not coincide with initial halide abstraction from DCE.

### On the role of ligand

Gold(I)-catalyzed reactions have historically been favored by strong donor ligands such as JPhos and IPr, while recent results have drawn attention to the potential of ligands with electron-withdrawing groups [4,10–12]. The above case studies represent two examples where distractor processes (either formation of alkylgold or degradation to LAuCl) may be serving to prevent accurate quantification of catalyst activity, and thus suggesting that some catalysts are even more reactive than they at first seem.

We found that cyclization of **7b** also occurs at room temperature with JackiePhosAuNTf_2_ (cat. **A**) which thus allowed monitoring by ^1^H NMR spectroscopy for kinetics. For direct comparison to our previous work, we switched to 0.05 M substrate concentration; we previously saw that hydroamination of **10** with cat. **A** proceeded faster than could be measured by our methods. We measured rates of hydroamination of **7b** and **12**, a substrate previously reported as challenging (lacking backbone substitution) [13] in CD_2_Cl_2_ at room temperature with other catalysts. Both reactions showed first order dependence on catalyst concentration. JackiePhosAuNTf_2_ outperformed all other commonly used catalysts including one that employed the N-heterocyclic carbene IPr. Comparison of **10**, **7b**, and **12** shows inherent reactivity differences from the so-called Thorpe–Ingold effect and the greater difficulty of making a 6-membered ring compared to a 5-membered ring (Table 1).

**Table 1:** Relative rates of hydroamination with various catalysts.

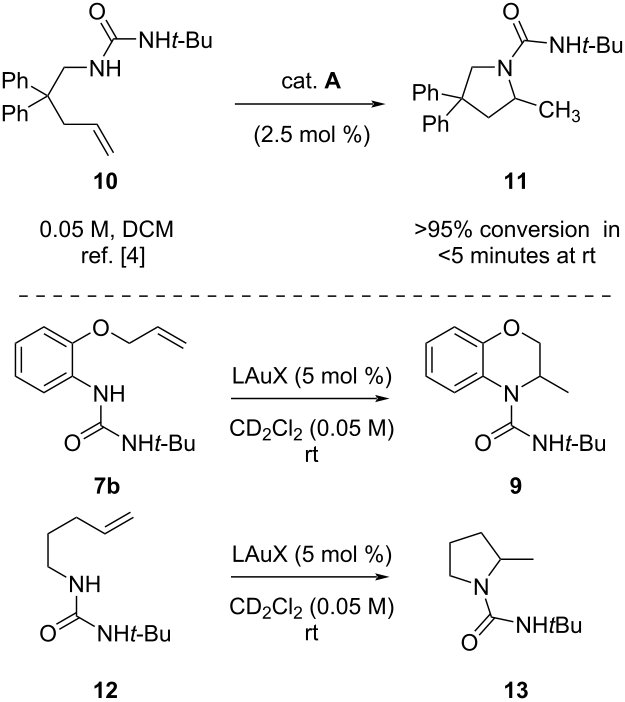			

entry	urea	catalyst	*k*_rel_^a^

1^b^	**7b**	Ph_3_PAuNTf_2_	0.7
2^b^	**7b**	IPrAuNTf_2_	1
3^b^	**7b**	JPhosAuNTf_2_	1.6
4	**7b**	JackiePhosAuOTf	14.2
5^b^	**7b**	Cat. **A**	50
6^b^	**12**	IPrAuNTf_2_	32.5
7^b^	**12**	Cat. **A**	650

^a^*k*_rel_ = 1 set to IPrAuNTf_2_ (0.06 × 10^−5^ s^−1^); ^b^averages of 3 trials.

### On the intermediacy of alkylgold

**Scheme 3:**
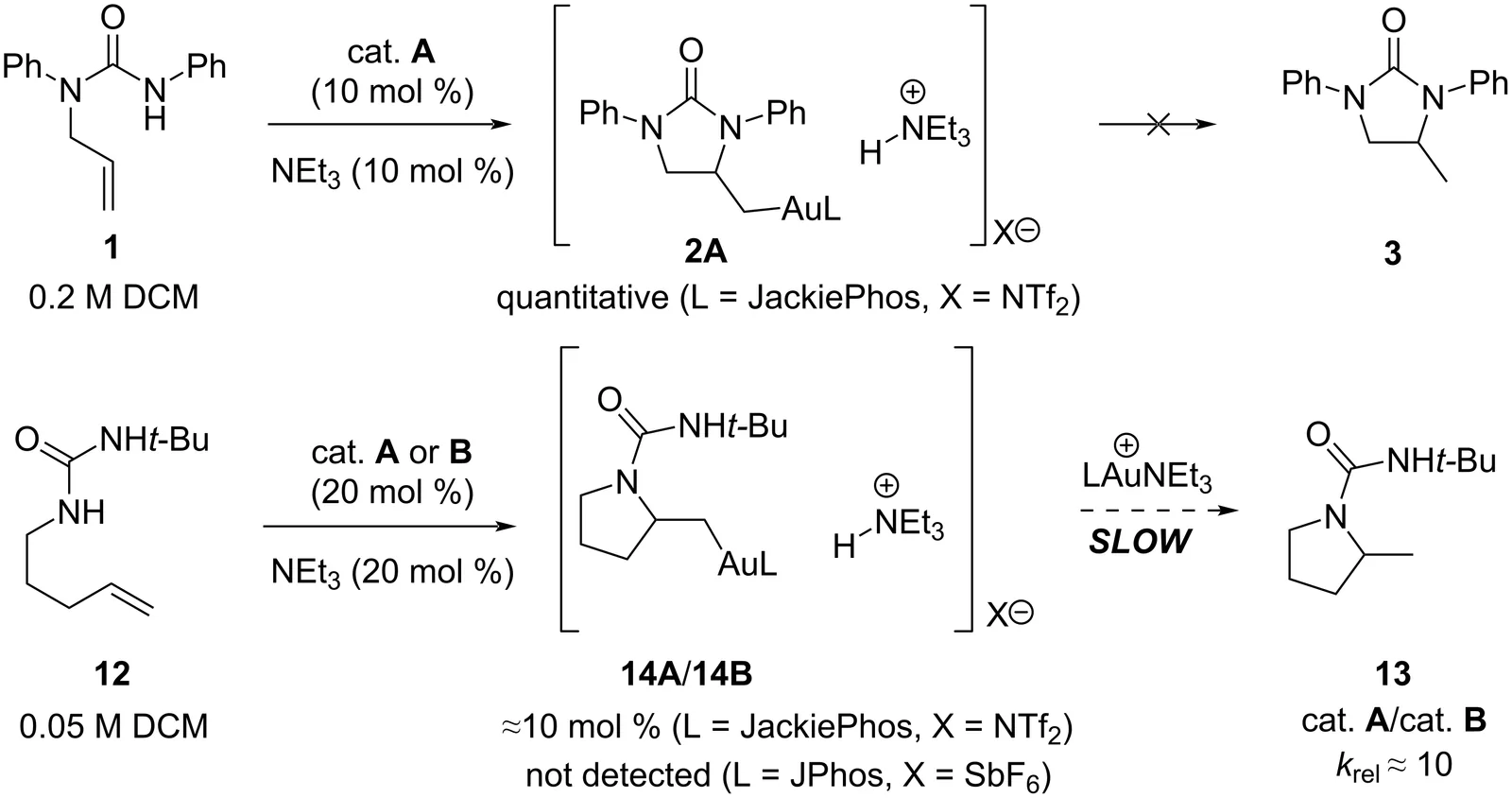
Alkylgold formation in the presence of LAu(I)/NEt_3_.

In contrast to reactions with **1**, we see no evidence of alkylgold intermediacy in the hydroamination of **7b** or **12** under standard reactive conditions. Control experiments were performed with gold(I) and triethylamine (TEA) to confirm the identity of alkyl gold (Figures S21–S26, Supporting Information File 1). When JackiePhosAuNTf_2_ (10 mol %) was combined with TEA (10 mol %) and added to diphenylurea **1** (Scheme 3), conversion to alkylgold was quantitative and hydroamination completely inhibited (no further reaction after 24 hours). This supports the conclusion that the JackiePhosAu–C bond is stable to protonolysis with HNEt_3_NTf_2_. In other experiments with JackiePhos or JPhosAu(NCCH_3_)SbF_6_, the amount of alkylgold formed is proportional to the amount of LAuNEt_3_ present, however, small quantities of remaining nominal [LAu]^+^ very slowly catalyze the formation of cyclized urea. With Ph_3_PAuNTf_2_, the decomposition product [Ph_3_PAuPPh_3_]NTf_2_ dominates over formation of alkylgold, but some amount of active catalyst remains that appears to facilitate similarly inefficient cyclization.

With urea **12**, no alkylgold **14B** is observed with JPhosAu(NCCH_3_)SbF_6_ whereas about 50% conversion (relative to the amount of gold) to alkylgold **14A** is observed with JackiePhosAuNTf_2_. Both show slow conversion to cyclized pyrrolidine, with JackiePhosAuNTf_2_ (cat. **A**) outperforming JPhosAu(NCCH_3_)SbF_6_ (cat. **B**) by about 10:1 (Figures S24–S26, Supporting Information File 1). Since base experiments with **1** demonstrate the resistance of alkylgold to protodeauration with [HNEt_3_]^+^, we conclude that the higher effectiveness of JackiePhos is in spite of and not due to higher concentrations of alkylgold.

In acid-catalyzed reactions, sulfonamides and thioureas are especially reactive [14–15]. To better understand the effectiveness of gold compared to acids we included other functional groups in our study. Carbamate **15**, thiocarbamate **16**, and thiourea **17** do not undergo hydroamination upon treatment with JPhosAu(NCCH_3_)SbF_6_ (cat. **B**) or JackiePhosAuNTf_2_ (cat. **A**) at room temperature (no evidence of cyclized product after 24 hours). Table 2 summarizes the intermediates observed with these substrates as detected by ^31^P NMR spectroscopy. In the absence of base, carbamate **15** shows weak to no interaction with [LAu]^+^ as the ^31^P chemical shift observed matches that of the original catalyst. With L = JackiePhos, carbamate **15** degrades over 24 hours in solution. With thiocarbamate **16** and thiourea **17**, the chemical shifts observed are consistent with gold(I) coordination to sulfur. In the presence of NEt_3_, carbamate **15** shows slow conversion to alkylgold with cat. **B** and rapid degradation with cat. **A**. Thiocarbamate **16** shows partial conversion to alkylgold **22** with cat. **A**, but not cat. **B**. Thiourea **17** shows partial conversion to alkylgold **23** with both catalysts. These results reaffirm the effectiveness of exogenous base to increase the equilibrium toward alkene addition, while also reiterating that accessibility of alkylgold is not a predictor of whether a substrate can undergo catalytic hydroamination [7].

**Table 2:** ^31^P NMR chemical shifts (δ, ppm) for gold intermediates observed after treatment of substrates **15**, **16**, **17** with cat. **A** and **B** with and without NEt_3_.^a^

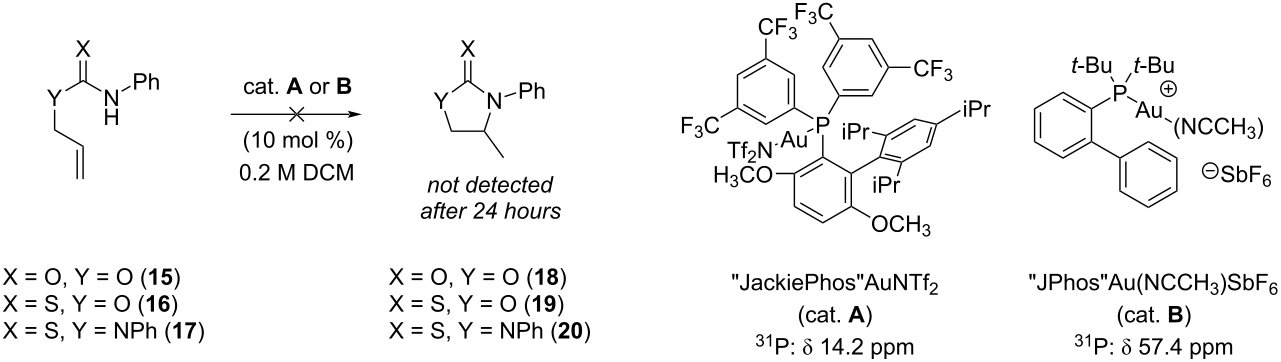					

entry	alkene	cat. **A** L = JackiePhos	cat. **A**/NEt_3_ L = JackiePhos	cat. **B** L = JPhos	cat. **B**/NEt_3_ L = JPhos

1	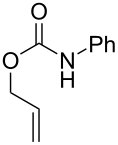 **15**	cat. **A** δ 14.2 ppm (free catalyst) → Unknown degradation	rapid unknown degradation.	cat. **B** δ 57.4 ppm (free catalyst)	LAu(NEt_3_)SbF_6_ δ 57.5 ppm 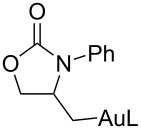 **21B** δ 67.7 ppm

2^b^	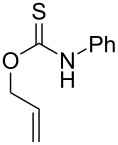 **16**	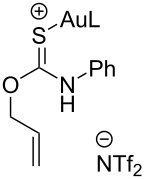 **[16AuA]^+^** δ 23 ppm → unknown degradation	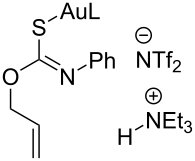 **16AuA** δ 24 ppm 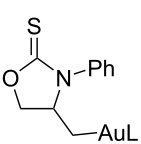 **22A** δ 35 ppm	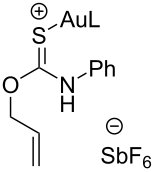 **[16AuB]^+^** δ 65.3 ppm → unknown degradation	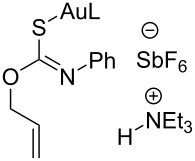 **16AuB** δ 62.7 ppm

3^b^	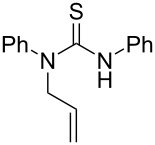 **17**	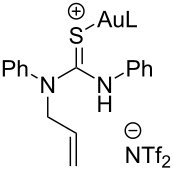 **[17AuA]^+^** δ 22 ppm	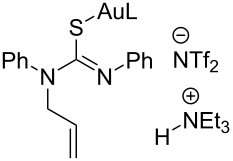 **17AuA** δ 22 ppm 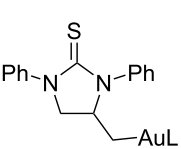 **23A** δ 36 ppm	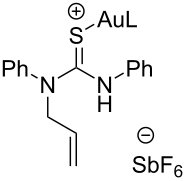 **[17AuB]^+^** δ 64.5 ppm	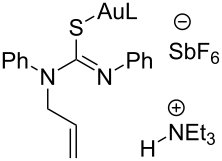 **17AuB** δ 64.3 ppm 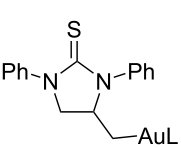 **23B** δ 67.4 ppm

^a^See Figures S27–S36 in Supporting Information File 1; ^b^in cationic complexes of **16** and **17** the exact position of the proton was not determined.

### Role of acid co-catalyst on reactivity of urea **1**

Control studies with HOTf show that Brønsted acid alone is an ineffective catalyst for urea alkene hydroamination and this is consistent with others’ observations. We surmised that added HOTf may be effective for perturbing the alkylgold equilibrium. When HOTf was added to **1** under hydroamination conditions (Table 3), it had no significant impact on the reaction rate, except with cat. **A** (Table 3, entry 3 vs 4). With JPhos, alkylgold formation is negligible regardless of anion or presence of exogenous acid. With JackiePhos, alkylgold was equally prominent regardless of anion, but with X = OTf it dissipated over the course of the more efficient reaction, while it persisted over the course of the much slower reaction with X = NTf_2_. Addition of acid to reactions with JackiePhos diminishes the initial concentration of alkylgold*.* Where alkylgold was significant and persistent (as with JackiePhosAuNTf_2_, cat. **A**) the decrease in alkylgold concentration corresponds to an increase in overall reaction rate. Where alkylgold was not significant or persistent (as with JackiePhosAuOTf, JPhosAuOTf, JPhosAuNTf_2_) addition of HOTf had a negligible impact on the reaction rate. This could be evidence of a HOTf-assisted increase in the rate of expected slower protodeauration of **2A** (alkylgold with electron-withdrawing ligand L = JackiePhos) [16]; it could also be evidence of a different mechanism that is dependent upon the re-release of [LAu]^+^ and it is not clear yet whether this is an acid or counteranion effect. We continue studies to quantify possible kinetic scenarios. The counteranion effect here is also interesting as ^−^OTf outperforms ^−^NTf_2_ for the reaction of **1**, but the opposite trend was seen with **7b** (vide supra, Table 1).

**Table 3:** Impact of acid co-catalyst on the rate of hydroamination of **1**.

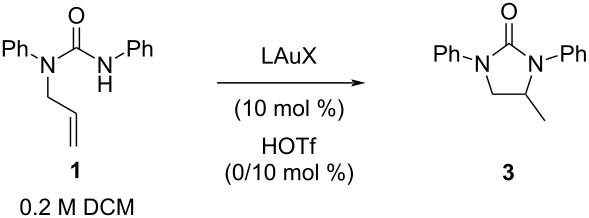				

entry	L	X	add.	*k*_obs_ × 10^7^ [s^−1^]

1	JackiePhos	OTf		332 ± 30
2	JackiePhos	OTf	HOTf	276 ± 50
3	JackiePhos	NTf_2_		11 ± 2
4	JackiePhos	NTf_2_	HOTf	106 ± 30
5	JPhos	OTf		56 ± 10
6	JPhos	OTf	HOTf	54 ± 4
7	JPhos	NTf_2_		8 ± 2
8	JPhos	NTf_2_	HOTf	12.5 ± 0.7

## Conclusion

Put together, these results present several practical caveats. First, they provide further evidence that gold(I) catalysts operate by mechanisms that are distinct from Brønsted acid catalysts. Second, they suggest that caution should be used when making conclusions about reactivity at high temperatures in DCE, as deactivation to LAuCl is feasible. While halide abstraction is precedented and at times deliberate in the use of silver catalysts, we have not seen it reported with gold catalysts [17].

For the acid-catalyzed reactions, we posit that the special reactivity of sulfonamides is primarily related to the weak basicity of the nucleophile. The failure of urea **7a** (R = CH_3_) to undergo acid-catalyzed isomerization/hydroamination appears to be a buffering effect since isomerization/hydroamination of **4a** (R = CH_3_) is shut down in the presence of exogenous dimethylurea (no reaction after 24 hours at 75 °C). For gold(I) reactivity, a fine balance is required. Weakly basic/nucleophilic sulfonamides do not engage, while non-productive association to thiocarbonyls is too strong.

The evolution of intermediates and rate of competing decomposition can be controlled by ligand and anion. JackiePhos ligand and ^−^NTf_2_ counterion favor the build-up of alkylgold, potentially due to enhanced Lewis acidity of gold(I) and diminished Brønsted acidity of HNTf_2_. Where alkylgold is readily accessible (as in **1→3**), this leads to diminished reaction rates; where it is not (as in **7→9b**) it leads to enhanced reaction rates suggesting dual roles for the counterion. There may be untapped potential in using ligands with electron-withdrawing groups, if distractor processes can be minimized and the continued ability to identify and characterize reactive intermediates means acid co-catalysts [18] may employed more strategically. We continue to examine the dynamics of the reactivity trends seen here and recognize the opportunity for plentiful quantitative information to be gleaned from considering the impact of ligand and anion on elementary reaction steps in addition to overall reaction efficiency.

## Supporting Information

File 1 Experimental procedures, kinetic data, and relevant spectra for starting materials, products, kinetics reactions, intermediates.

## Data Availability

Data generated and analyzed during this study is available from the corresponding author upon reasonable request.
