# Supplementary material for: Gold(I)-catalyzed alkene hydroamination: revelations from kinetic benchmarking
Source: Beilstein J Org Chem. 2026 Nov 6;22:1597–604. doi: 10.3762/bjoc.22.129 (PMC13746454; doi:10.3762/bjoc.22.129)
Supplement: Supplementary file 1 [file Beilstein_J_Org_Chem-22-1597-s001.pdf]

BEILSTEIN JOURNAL OF ORGANIC CHEMISTRY

Supporting Information

for

Gold(I)-catalyzed alkene hydroamination: revelations from kinetic benchmarking

Jaden McCane, Yujing Qu, Ruichen Lan and Amanda C. Jones

Beilstein J. Org. Chem. **2026,** *22,* 1597–1604. doi:10.3762/bjoc.22.129

Experimental procedures, kinetic data, and relevant spectra for starting materials, products, kinetics reactions, intermediates

License and Terms: This is a supporting information file under the terms of the Creative Commons Attribution License (https://creativecommons.org/licenses/by/4.0). Please note that the reuse, redistribution and reproduction in particular requires that the author(s) and source are credited and that individual graphics may be subject to special legal provisions.
The license is subject to the *Beilstein Journal of Organic Chemistry* terms and conditions: (https://www.beilstein-journals.org/bjoc/terms)

Contents

General experimental S3
Synthesis S4
Alkene substrate syntheses S4
Product syntheses. S6
Catalyst syntheses: S8
Synthesis of JackiePhosAuCl S8
Synthesis of JackiePhosAuNTf_2 (cat. **A**) and JackiePhosAuOTf S8
Kinetics S9
Kinetics with 1-allyl-1,3-diphenylurea (**1**) S9
Kinetics with 1-(2-(allyloxy)phenyl)-3-(*tert*-butyl)urea (**7b**) S11
Order in catalyst. S12
Anion effect. S13
Ligand effect S14
Ligand effect summary. S17
Kinetics with 1-(*tert*-butyl)-3-(pent-4-en-1-yl)urea (**12**) S18
Ligand effect S18
Control studies S22
Vial Reaction Screen S22
Table S1. Reactivity screen for 4-methyl-*N*-(2-((2-methylallyl)oxy)phenyl) benzenesulfonamide (**4a**) and 1-(2-(allyloxy)phenyl)-3-(*tert*-butyl)urea (**7b**) S22
Table S2 Summary of ^{31}P NMR chemical shifts for species observed during vial reactions. S23
Generation of JackiePhosAuCl during reaction kinetics S24
Regeneration of JackiePhosAuCl from JackiePhosAuX in DCE at 75 °C: S26
Table S3. ^{31}P NMR chemical shifts for solutions of JackiePhosAuX in DCE after heating at 75 °C at t = 3, 24, 48 hours. S26
Thiourea reactivity. S28
Gold coordination to phenol thiourea. S28
Table S4. ^{31}P NMR chemical shifts for solutions of LAuNTf_2 coordinated to 1-(2-hydroxyphenyl)-3-phenylthiourea in DCM-d_2. .... S28

Impact of urea on acid reactivity S29
Reactivity of alkenyl amines with allylic tethers with JackiePhosAuNTf$_2$ (cat. **A**)..... S30
Additional control experiments for 1-allyl-1,3-diphenylurea (**1**) S30
Characterization of alkylgold from reactivity of 1-allyl-1,3-diphenylurea (**1**) S31
Identification of alkylgold from reactivity of 1-(*tert*-butyl)-3-(pent-4-en-1-yl)urea (**12**) S34
Gold interactions with carbamate, thiocarbamate, thiourea S37
NMR spectra of pure materials S47
References S67

General experimental

Catalyst complexes were prepared in an oxygen and moisture-free glovebox under nitrogen atmosphere. Deuterated solvents were purchased from Cambridge Isotope Laboratories. NMR spectra were obtained on a Bruker spectrometer operating at a proton frequency of 400 MHz. Unless otherwise noted, spectra were collected at 25 °C. High-resolution mass spectra (HRMS) were recorded on a LC–MS instrument (Vanquish Flex, ThermoFisher) equipped with an Exploris 240 Orbitrap analyzer using ESI(+). For kinetic plots, the total concentrations of alkene starting material, cyclized product and any intermediates were normalized, assuming the sum of the signals for each were constant.

Observed rates and corresponding errors for individual experiments were calculated using the LINEST function in EXCEL. Where multiple trials were conducted, the standard deviation on the average was calculated.

Synthesis

Alkene substrate syntheses

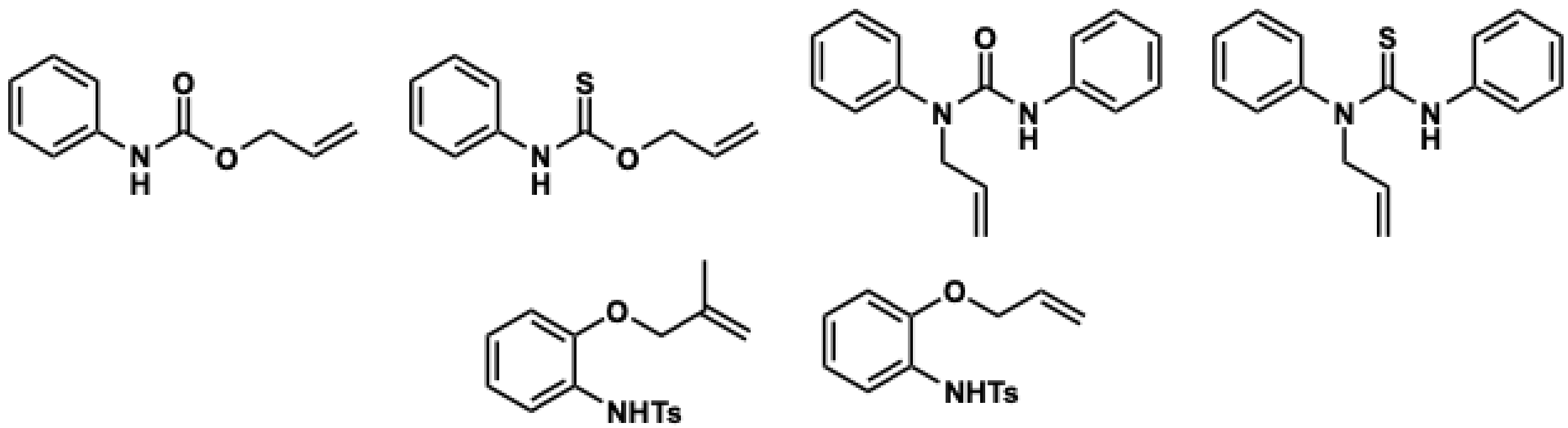

Compounds allyl phenylcarbamate (**15**)[S1], *O*-allyl phenylcarbamothioate (**16**)[S2][S3], 1-allyl-1,3-diphenylurea (**1**) [S4], allyl diphenylthiourea (**17**)[S5], 4-methyl-*N*-(2-((2-methylallyl)oxy)phenyl)benzenesulfonamide (**4a**), and *N*-(2-(allyloxy)phenyl)-4-methylbenzenesulfonamide[S6] are known and were synthesized according to the literature procedures.

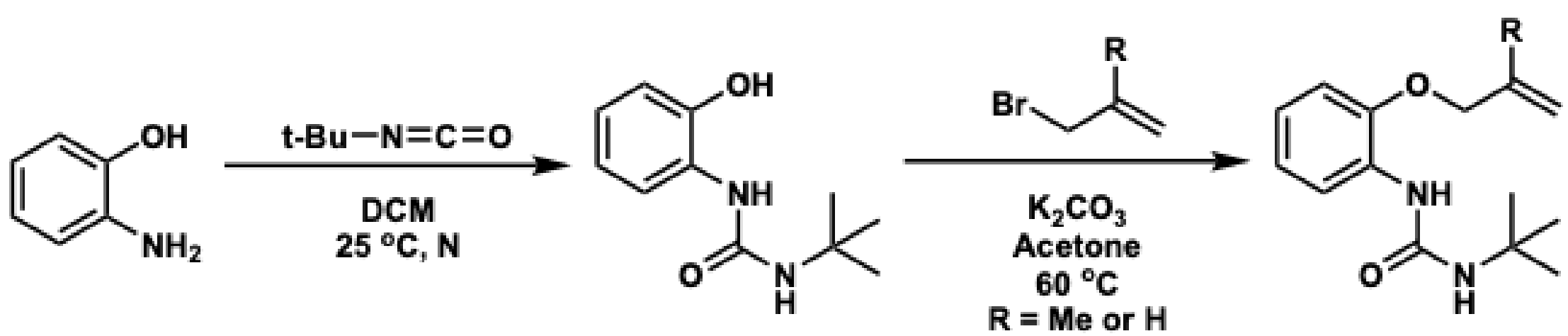

1-(*tert*-Butyl)-3-(2-((2-methylallyl)oxy)phenyl)urea (7a) and 1-(2-(allyloxy)phenyl)-3-(*tert*-butyl)urea (7b). To a suspension of 2-aminophenol (5.0 mmol, 1.0 equiv) in DCM (20 mL, 0.25 M), was added *tert*-butyl isocyanate (5.0 mmol, 1.0 equiv) at 0 °C. The reaction was allowed to stir overnight at room temperature under nitrogen atmosphere. After being concentrated in vacuo, 1-(*tert*-butyl)-3-(2-hydroxyphenyl)urea was used in the following step without further purification (90 % yield).

To a solution of 1-(*tert*-butyl)-3-(2-hydroxyphenyl)urea (4.5 mmol, 1.0 equiv) in acetone (4.5 mL, 1.0 M), were added K_2CO_3 (5.4 mmol, 1.2 equiv) and then allyl bromide (4.5 mmol, 1.0 equiv) dropwise at 0 °C. After being allowed to stir overnight at 60 °C, the reaction mixture was cooled to room temperature and diluted with EtOAc, washed with H_2O, dried over $MgSO_4$, and concentrated in vacuo. Purification was achieved by column chromatography on silica gel (EtOAc/*n*-hexane 1:5–1:3). Typical yields were ≈80%.

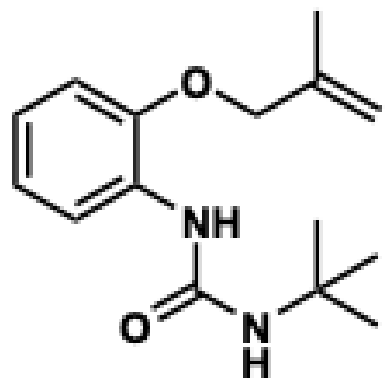

1-(*tert*-Butyl)-3-(2-((2-methylallyl)oxy)phenyl)urea (7a):

¹H NMR (400 MHz, $CDCl_3$) δ 8.01 – 7.93 (m, 1H), 6.98 – 6.87 (m, 2H), 6.87 – 6.79 (m, 1H), 6.64 (bs, 1H), 5.07 (m, 1H), 5.01 (m, 1H), 4.63 (bs, 1H), 4.48 (app s, 2H), 1.83 (s, 3H), 1.39 (s, 9H). **¹³C NMR** (101 MHz, $CDCl_3$) δ 154.56, 147.38, 140.71, 129.09, 122.39, 121.51, 119.86, 113.28, 111.79, 72.53, 50.97, 29.54, 19.62. **HRMS** (ESI+) calcd. for $[M+H]^+$ = $C_{15}H_{23}N_2O_2$: 263.1759, found 263.1751.

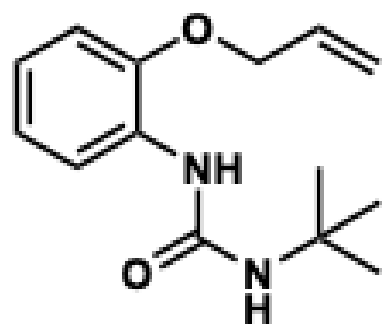

1-(2-(Allyloxy)phenyl)-3-(*tert*-butyl)urea (7b):

¹H NMR (400 MHz, $CDCl_3$) δ 8.05 – 7.95 (m, 1H), 6.96 – 6.88 (m, 2H), 6.87 – 6.80 (m, 1H), 6.77 (bs, 1H), 6.05 (ddt, *J* = 17.3, 10.9, 5.5 Hz, 1H), 5.38 (dq, *J* = 17.3, 1.5 Hz, 1H), 5.29 (dq, *J* = 10.5, 1.4 Hz, 1H), 4.75 (bs, 1H), 4.57 (dt, *J* = 5.5, 1.5 Hz, 2H), 1.38 (s, 9H). **¹³C NMR** (101 MHz, $CDCl_3$) δ 154.65, 147.12, 133.22, 129.22, 122.21, 121.51, 119.72, 118.33, 111.67, 69.70, 50.87, 29.51. **HRMS** (ESI+) calcd. for $[M+H]^+$ = $C_{14}H_{21}N_2O_2$: 249.1603, found 249.1594.

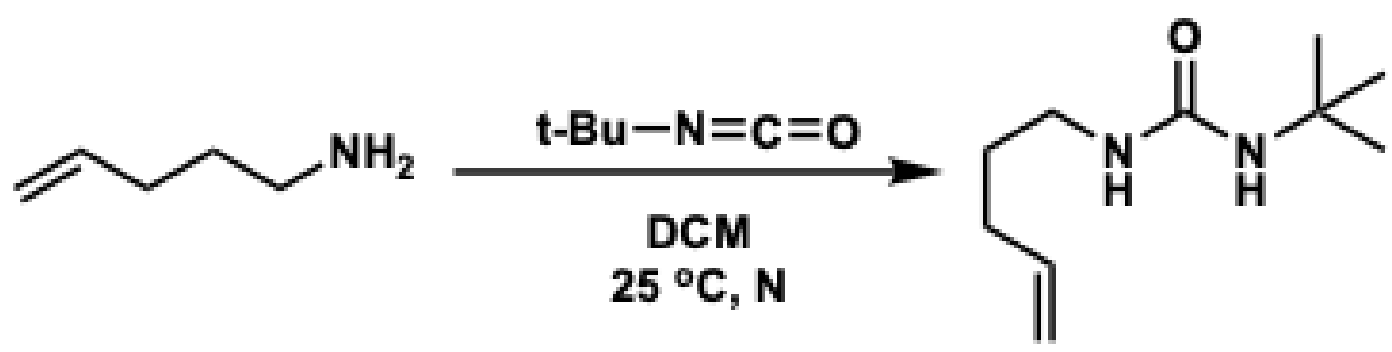

1-(*tert*-Butyl)-3-(pent-4-en-1-yl)urea (12). To a solution of pent-4-en-1-amine (2.0 mmol, 1.0 equiv) in DCM (8 mL, 0.25 M) was added *tert*-butyl isocyanate (2.0 mmol, 1.0 equiv) at 0 °C. The reaction was allowed to stir overnight at room temperature under nitrogen atmosphere. The reaction was diluted with EtOAc, washed with H_2O, and dried over $MgSO_4$. Purification was achieved by column chromatography on silica gel (EtOAc/*n*-hexane 1:3). Typical yields were ≈90%. **¹H NMR** (400 MHz, $CDCl_3$) δ 5.78 (ddt, *J* = 16.9, 10.1, 6.6 Hz, 1H), 5.06 – 4.91 (m, 2H), 4.60 (bs, 2H), 3.11 (t, *J* = 7.1 Hz, 2H), 2.07 (qt, *J* = 6.8, 1.4 Hz, 2H), 1.55 (p, *J* = 7.3 Hz, 2H), 1.31 (d, *J* = 0.8 Hz, 9H). **¹³C NMR** (101 MHz, $CDCl_3$) δ 157.97, 138.15, 115.09, 50.33, 39.84, 31.25, 29.71, 29.65. **HRMS** (ESI+) calcd. for $[M+H]^+$ = $C_{10}H_{21}N_2O$: 185.1654, found 185.1644.

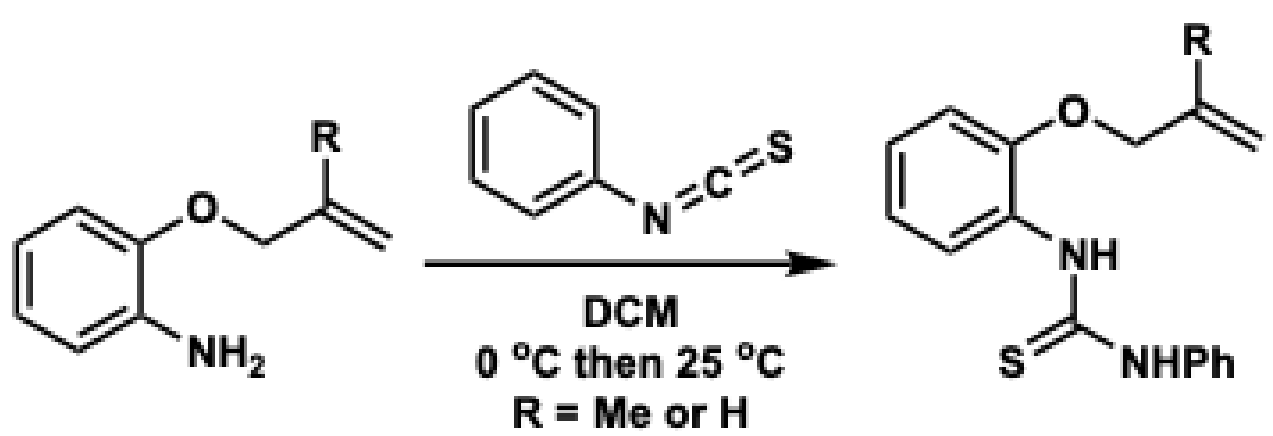

1-(2-(Allyloxy)phenyl)-3-phenylthiourea (R = H) and 1-phenyl-3-(2-((2-methylallyl)oxy)phenyl)thiourea (R = CH_3). Phenyl isothiocyanate (1 mmol, 1 equiv) was added to a solution of aniline (1.0 mmol, 1 equiv) in DCM (3.3 mL, 0.3 M) dropwise at 0 ºC. After being allowed to stir overnight at room temperature, the solvent was removed by vacuum and the crude product was purified by column chromatography on silica gel (1:6 EtOAc:Hexanes) to give products in 82 – 88 % yield.

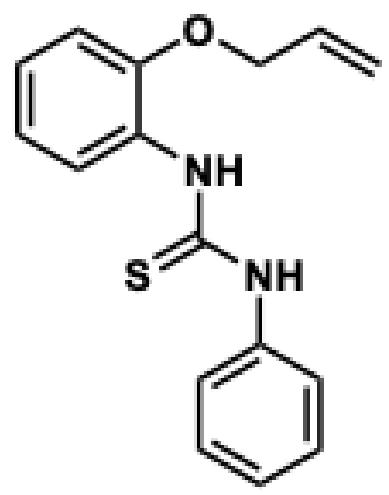

1-(2-(Allyloxy)phenyl)-3-phenylthiourea:

^{1}H NMR (400 MHz, $CDCl_3$) δ 8.33 (d, *J* = 8.0 Hz, 1H), 8.21 (bs, 1H), 8.03 (bs, 1H), 7.42 (dd, *J* = 8.4, 7.0 Hz, 2H), 7.39 – 7.34 (m, 2H), 7.33 – 7.27 (m, 1H), 7.13 (td, *J* = 7.8, 1.6 Hz, 1H), 7.00 (td, *J* = 7.7, 1.4 Hz, 1H), 6.88 (dd, *J* = 8.2, 1.3 Hz, 1H), 5.88 (ddt, *J* = 17.3, 10.6, 5.4 Hz, 1H), 5.29 – 5.19 (m, 2H), 4.48 (dt, *J* = 5.5, 1.5 Hz, 2H). **^{13}C NMR** (101 MHz, $CDCl_3$) δ 178.76, 149.88, 136.72, 132.51, 130.00, 127.39, 127.33, 126.21, 125.49, 123.89, 120.97, 118.36, 112.18, 69.60. **HRMS** (ESI+) calcd. for $[M+H]^+$ = $C_{16}H_{17}N_2OS$: 285.1062, found 285.1050.

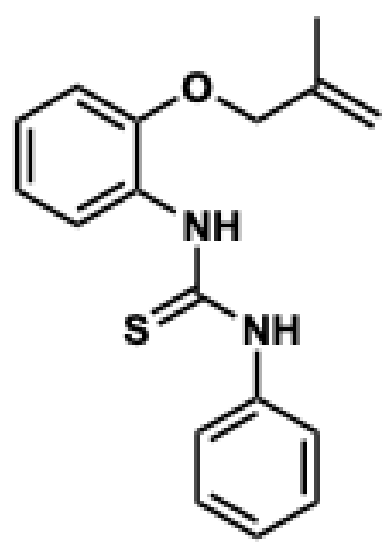

1-Phenyl-3-(2-((2-methylallyl)oxy)phenyl)thiourea:

^{1}H NMR (400 MHz, $CDCl_3$) δ 8.33 (d, *J* = 8.0 Hz, 1H), 8.18 (bs, 1H), 8.03 (bs, 1H), 7.44 – 7.38 (m, 2H), 7.38 – 7.34 (m, 2H), 7.32 – 7.26 (m, 1H), 7.12 (td, *J* = 7.9, 1.6 Hz, 1H), 7.00 (td, *J* = 7.8, 1.3 Hz, 1H), 6.88 (dd, *J* = 8.2, 1.3 Hz, 1H), 4.90 (app d, 2H), 4.37 (s, 2H), 1.65 (s, 3H). **^{13}C NMR** (101 MHz, $CDCl_3$) δ 178.84, 150.00, 140.03, 136.69, 130.00, 127.37, 126.24, 125.47, 123.97, 120.87, 113.60, 112.12, 72.47, 19.52. **HRMS** (ESI+) calcd. for $[M+H]^+$ = $C_{17}H_{19}N_2OS$: 299.1218, found 299.1206.

Product syntheses.

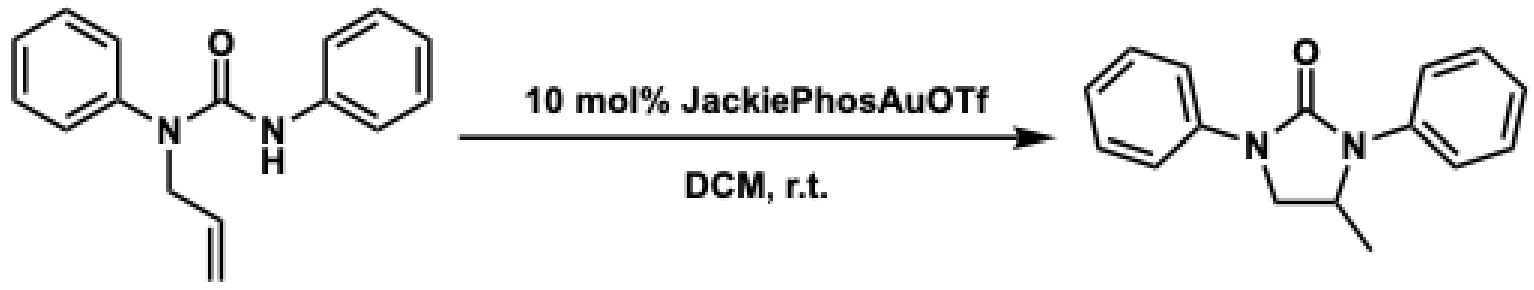

4-Methyl-1,3-diphenylimidazolidin-2-one (3). JackiePhosAuOTF (0.042 mmol, 0.0479 g) was weighed out in a scintillation vial. 1-Allyl-1,3-diphenylurea (0.42 mmol, 0.1059 g) was added. DCM (2.1 mL) was added to dissolve the substrate and catalyst. The vial was sealed with parafilm. The reaction was stirred for 36 hours at room temperature. 4-Methyl-1,3-diphenylimidazolidin-2-one was purified by column chromatography on silica gel (1:9 EtOAc/hexanes) and isolated in 96% yield. **^{1}H NMR** (400 MHz, $CDCl_3$) δ 7.62 – 7.47 (m, 2H), 7.44 – 7.37 (m, 2H), 7.37 – 7.22 (m, 4H), 7.12 – 6.97 (m, 2H), 4.41 (dp, *J* = 8.7, 6.1 Hz, 1H), 4.05 (t, *J* = 8.7 Hz, 1H), 3.49 (dd, *J* = 8.8, 6.1 Hz, 1H), 1.32 (d, *J* = 6.1 Hz, 3H). **^{13}C NMR** (101 MHz, $CDCl_3$) δ 128.95, 128.88, 124.34, 122.85, 122.08, 117.94, 77.23, 49.97, 48.81, 19.31. **HRMS** (ESI+) calcd. for $[M+H]^+$ = $C_{16}H_{17}N_2O$: 253.1341, found 253.1330.

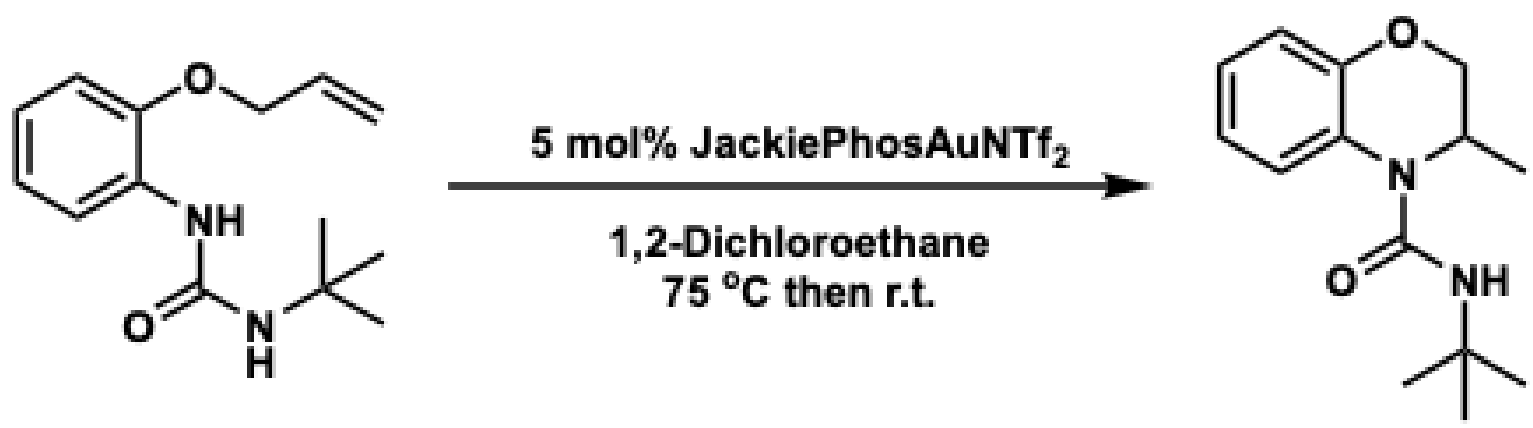

***N*-(*tert*-Butyl)-3-methyl-2,3-dihydro-4*H*-benzo[*b*][1,4]oxazine-4-carboxamide (9b).** Aryl urea alkene (0.24 mmol, 59.59 mg) and JackiePhosAuNTf$_2$ (cat. **A**, 0.012 mmol, 15.32 mg) were added to an RBF and dissolved in DCE (0.08 M, 3mL). A condenser was attached and the reaction was allowed to stir at 75 °C for 5 hours and then overnight at room temperature. The product was purified by column chromatography on silica gel (1:6 EtOAc/hexanes) and isolated in 54% yield. **^{1}H NMR** (400 MHz, CDCl$_3$) δ 7.21 (dd, *J* = 8.0, 1.6 Hz, 1H), 7.02 (td, *J* = 7.1, 1.6 Hz, 1H), 6.96 – 6.86 (m, 2H), 5.25 (bs, 1H), 4.83 (qdd, *J* = 7.2, 2.9, 1.4 Hz, 1H), 4.14 (dd, *J* = 10.6, 1.4 Hz, 1H), 4.05 (dd, *J* = 10.7, 2.9 Hz, 1H), 1.35 (s, 9H), 1.13 (d, *J* = 7.1 Hz, 3H). **^{13}C NMR** (101 MHz, CDCl$_3$) δ 154.39, 146.30, 125.03, 124.82, 123.58, 120.83, 117.71, 70.03, 51.24, 43.79, 29.38, 15.77. **HRMS** (ESI+) calcd. for $[M+H]^+$ = $C_{14}H_{21}N_2O_2$: 249.1603, found 249.1593.

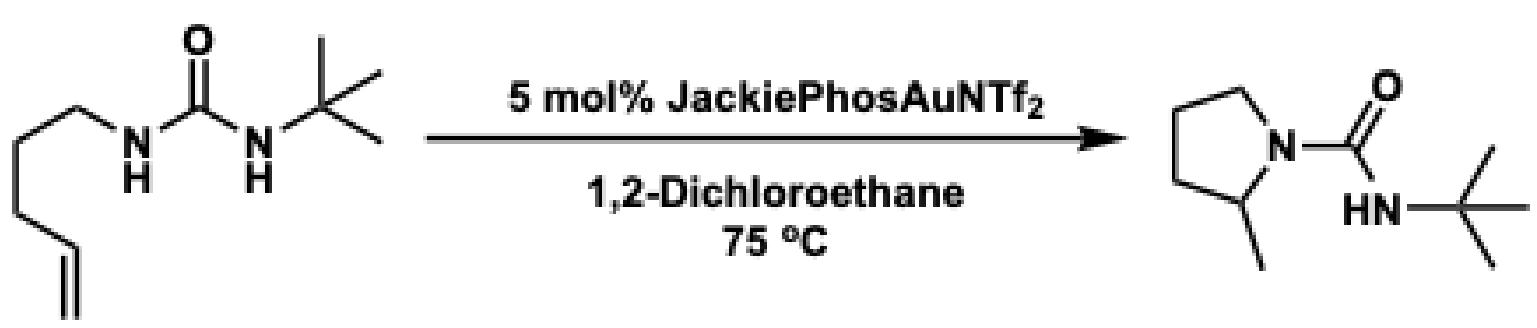

***N*-(*tert*-Butyl)-2-methylpyrrolidine-1-carboxamide (13)**. Alkenyl urea (0.3 mmol, 55.28 mg) and JackiePhosAuNTf$_2$ (cat. **A**, 0.015 mmol, 19.11 mg) were added to a scintillation vial and dissolved in DCE (0.1 M, 3 mL). The vial was sealed with a screw cap and parafilm and allowed to heat at 75 ˚C for 1 hour. After allowing to cool to room temperature the product was purified by column chromatography on silica gel (1:4 EtOAc/hexanes) and isolated in 90% yield. **^{1}H NMR** (400 MHz, CDCl$_3$) δ 4.01 (bs, 1H), 3.92 (pd, *J* = 6.4, 2.6 Hz, 1H), 3.34 – 3.26 (m, 1H), 3.26 – 3.17 (m, 1H), 2.04 – 1.90 (m, 2H), 1.90 – 1.78 (m, 1H), 1.61 – 1.51 (m, 1H), 1.34 (s, 9H), 1.15 (d, *J* = 6.3 Hz, 3H). **^{13}C NMR** (101 MHz, CDCl$_3$) δ 156.23, 52.50, 50.67, 46.06, 33.02, 29.79, 23.71, 20.82. **HRMS** (ESI+) calcd. for $[M+H]^+$ = $C_{10}H_{21}N_2O$: 185.1654, found 185.1642.

Catalyst syntheses:

Synthesis of JackiePhosAuCl

JackiePhosAuCl[S7] is known and synthesized according to the literature procedures.

Synthesis of JackiePhosAuNTf_2 (cat. **A**) and JackiePhosAuOTf

JackiePhosAuNTf_2 is commercially available. Preparations of JackiePhosAuOTf and JackiePhosAuNTf_2 are based on precedented procedures and spectra are shown below to confirm catalyst purity.

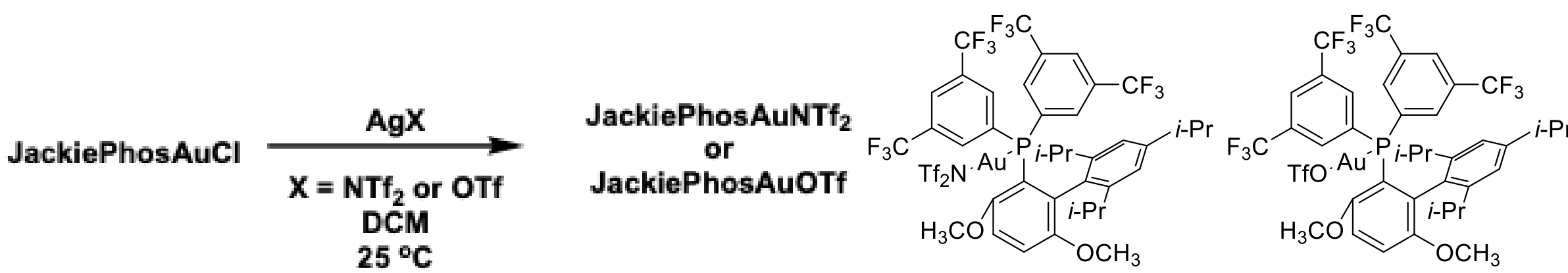

JackiePhosAuCl (0.097 mmol, 1 equiv) was weighed into a scintillation vial. The vial containing the gold chloride was brought into the glove box and AgX (0.102 mmol, 1.05 equiv) was subsequently added to the vial. The vial was septum sealed, removed from the glove box, connected to a nitrogen line, and DCM (5 mL, 0.02 M) was added through the septum. The vial was sonicated for 6 minutes and the AgCl precipitate was subsequently removed via celite filtration. DCM was evaporated by vacuum and purification was achieved by recrystallization using 1:2 DCM:Hexanes. Typical yields are ~ 80 %.

JackiePhosAuNTf_2 (cat. **A**). **^{1}H NMR** (400 MHz, CD_2Cl_2) δ 8.06 (s, 2H), 7.87 (d, *J* = 13.4 Hz, 4H), 7.36 (d, *J* = 9.2 Hz, 1H), 7.18 (s, 2H), 7.05 (dd, *J* = 9.2, 5.2 Hz, 1H), 3.70 (s, 3H), 3.44 (s, 3H), 2.97 (hept, *J* = 6.8 Hz, 1H), 2.33 (hept, *J* = 6.9 Hz, 2H), 1.32 (d, *J* = 6.9 Hz, 6H), 1.01 (d, *J* = 6.9 Hz, 6H), 0.96 (d, *J* = 6.7 Hz, 6H). **^{31}P NMR** (162 MHz) δ 14.16.

JackiePhosAuOTf. **^{1}H NMR** (400 MHz, None) δ 8.06 (s, 2H), 7.86 (d, *J* = 13.5 Hz, 4H), 7.38 (d, *J* = 9.2 Hz, 1H), 7.18 (s, 2H), 7.06 (dd, *J* = 9.1, 5.2 Hz, 1H), 3.72 (s, 3H), 3.39 (s, 3H), 2.96 (hept, *J* = 6.9 Hz, 1H), 2.33 (hept, *J* = 6.8 Hz, 2H), 1.31 (d, *J* = 7.0 Hz, 6H), 1.03 (d, *J* = 6.9 Hz, 6H), 0.98 (d, *J* = 6.7 Hz, 6H). **^{31}P NMR** (162 MHz) δ 10.92.

Kinetics

Kinetics with 1-allyl-1,3-diphenylurea (**1**)

The following standard procedure was used for kinetics with 1-allyl-1,3-diphenylurea (**1**). Alkene (0.14 mmol) and catalyst (0.014 mmol) were weighed simultaneously into the same scintillation vial. Solvent (0.7 mL) was added to the vial for a 0.2 M solution with 10 mol % catalyst loading. If appropriate, HOTF was then added. The resulting solution was transferred to an NMR tube using a glass pipette. The tube was immediately sealed, and the NMR experiment was initiated. The first spectrum was acquired within 5 minutes.

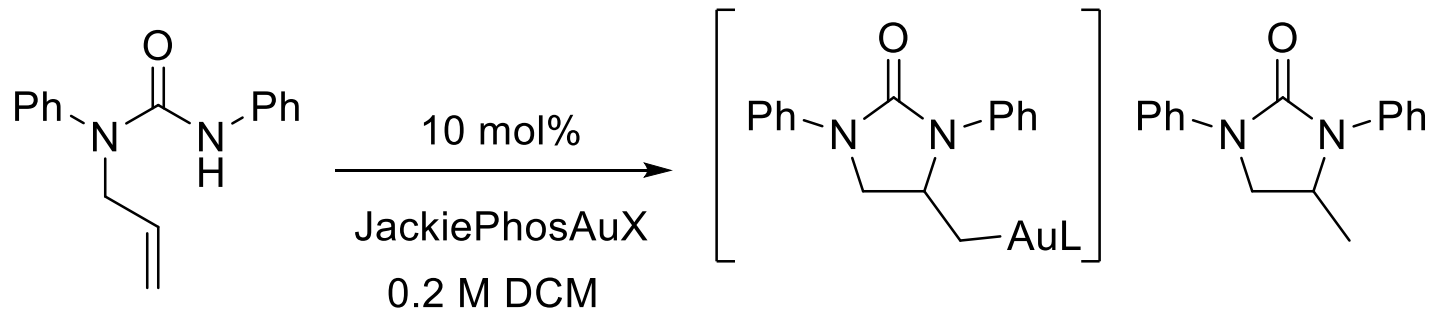

Entry	experiment Code	X	HOTf	$10^7 \times k_{obs}$ [s^{-1}]	alkylgold at start
1	AYQ 1007	NTf_2	/	12.0 ± 0.4	≈ 4 mol %
2	AYQ 2095	NTf_2	/	9.8 ± 0.4	≈ 4 mol %
3	AYQ 2065	NTf_2	10 mol %	125 ± 2	< 1 mol %
4	AYQ 2097	NTf_2	10 mol %	87 ± 2	< 1 mol %
5	AYQ 2093	OTf	/	311 ± 6	≈ 5.5 mol %
6	AYQ 2061	OTf	/	353 ± 4	≈ 5 mol %
7	AYQ 2057	OTf	10 mol %	241 ± 10	≈ 1 mol %
8	AYQ 2091	OTf	10 mol %	312 ± 7	≈ 1.5 mol %

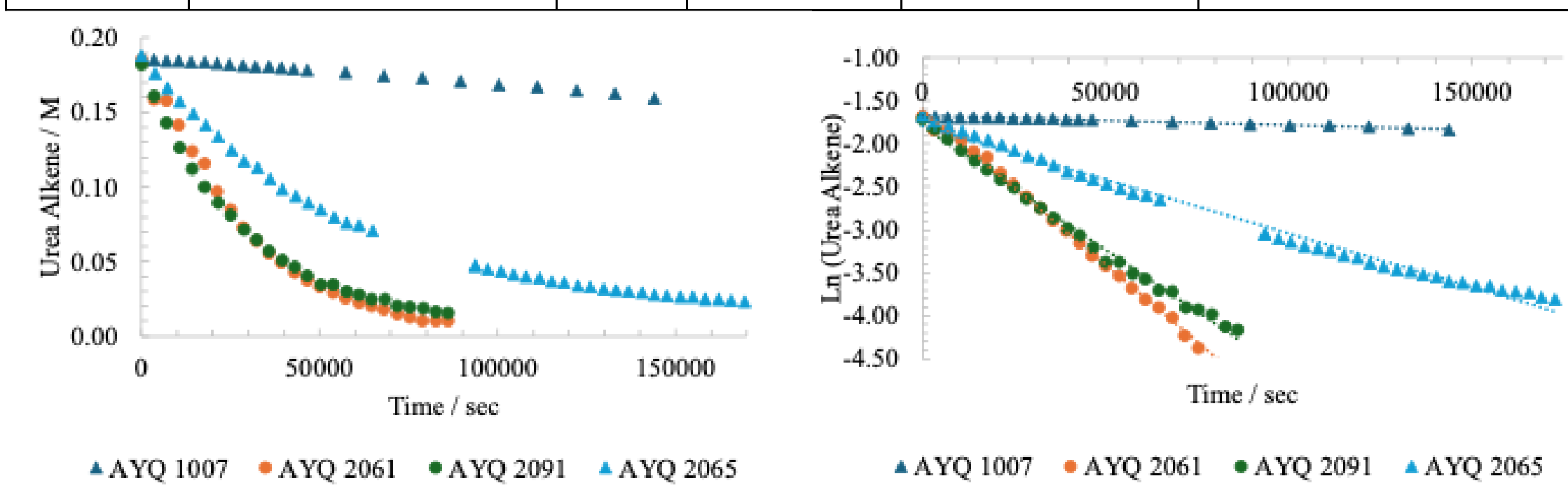

Figure S1. Raw and corresponding first order plots for urea **1** decay (0.2 M in 0.7 mL DCM) with 10 mol % JackiePhosAuX with and without 10 mol % HOTf. Duplicate data not shown.

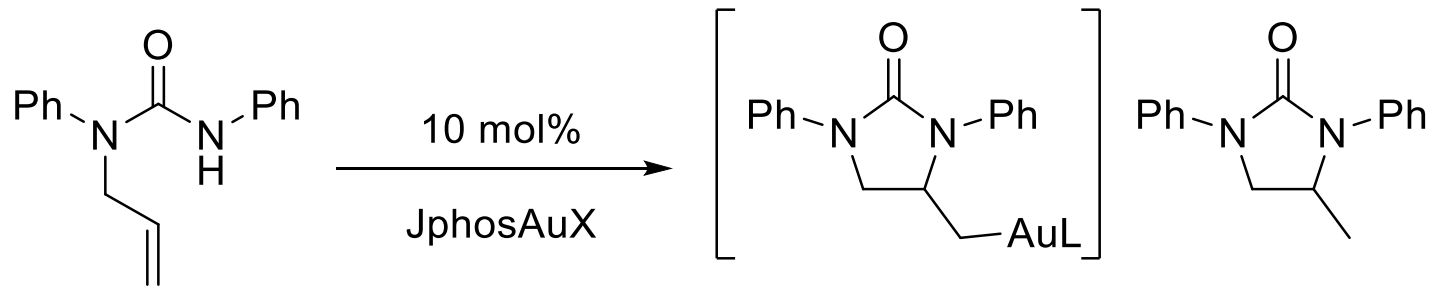

0.2 M DCM

Entry	experiment code	X	HOTf Loading	$10^7 \times k_{obs}$ [s^{-1}]	alkylgold at start
1	AYQ 2071	NTf_2	/	6.0 ± 0.2	n.d.
2	AYQ 2109	NTf_2	/	9.0 ± 0.1	<0.2 mol %
3	AYQ 2073	NTf_2	10 mol %	13.0 ± 0.2	n.d.
4	AYQ 2111	NTf_2	10 mol %	12.0 ± 0.1	n.d.
5	AYQ 2075	OTF	/	63.0 ± 0.4	<1 mol %
6	AYQ 2115	OTF	/	49.0 ± 0.3	<1 mol %
7	AYQ 2077	OTF	10 mol %	57.0 ± 0.7	n.d.
8	AYQ 2117	OTF	10 mol %	51.0 ± 0.5	n.d.

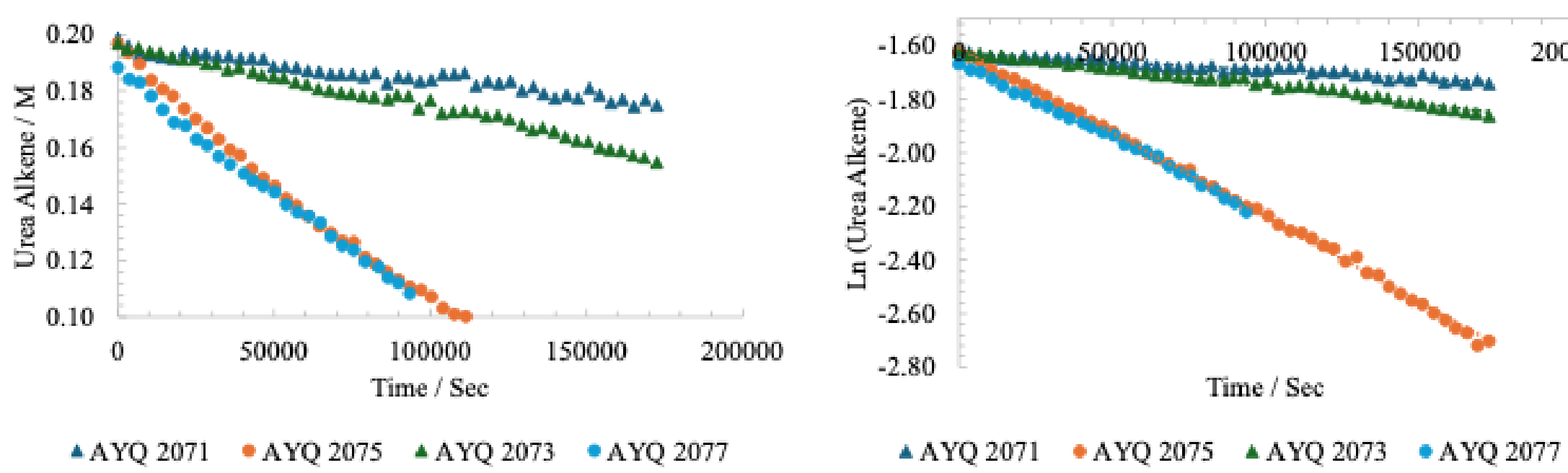

Figure S2. Raw and corresponding first order plots for urea **1** decay (0.2 M in 0.7 mL DCM) with 10 mol % JPhosAuX with and without 10 mol % HOTf. Duplicate data not shown.

Kinetics with 1-(2-(allyloxy)phenyl)-3-(*tert*-butyl)urea (**7b**)

Urea alkene **7b** (248.33 g/mol, 8.69 mg, 0.035 mmol) was weighed into a scintillation vial. JackiePhosAuNTf$_2$ (cat. **A**, 1273.78 g/mol, 2.23 mg, 0.00175 mmol) was subsequently weighed into the same vial and 0.7 mL DCM-d_2 was added. The solution was transferred to an NMR tube and placed into the spectrometer for ^{1}H NMR collection. Roughly 5 minutes elapsed between injection and acquisition of spectrum #1.

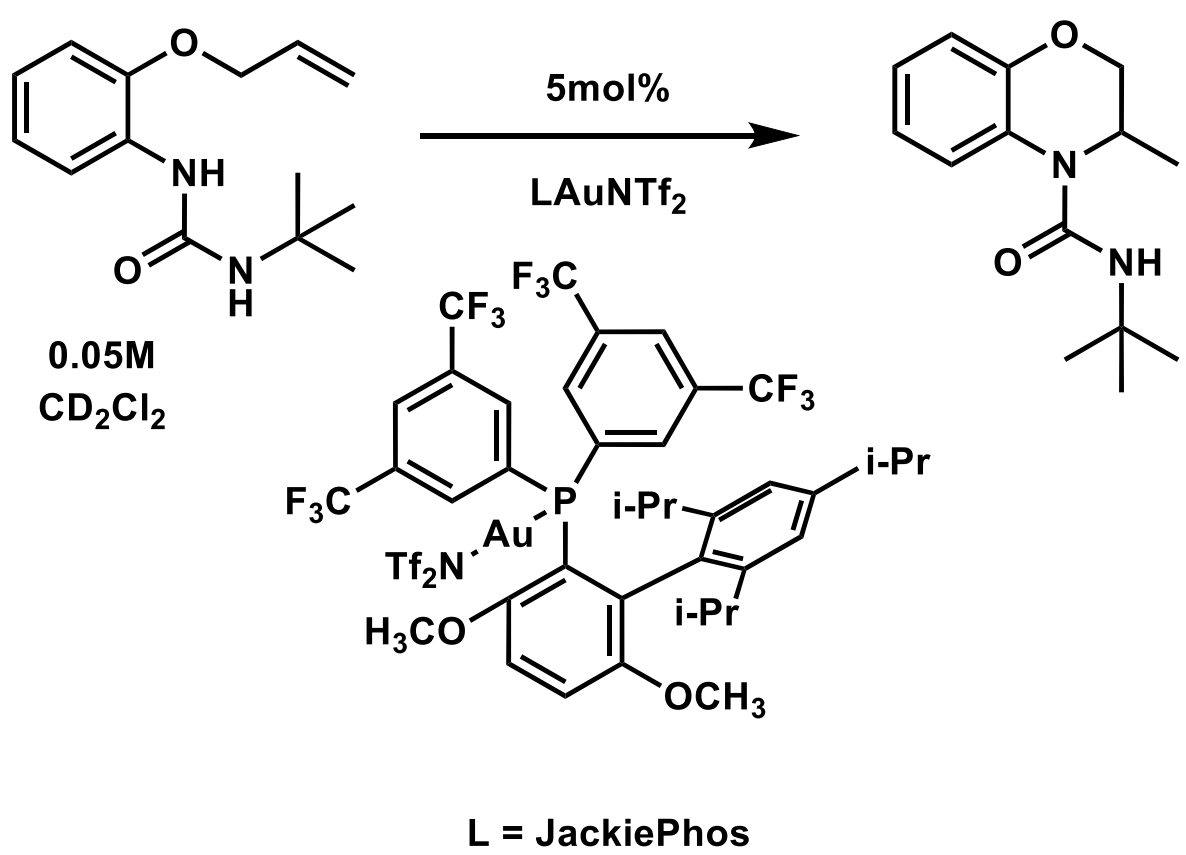

Entry	experiment code	catalyst amount	catalyst loading	CD_2Cl_2	$10^5 \times k_{obs}$ [s^{-1}]	
1	JM 3039	0.00175 mmol	5 mol %	0.7 mL	2.80 ± 0.03	average:
2	JM 5081	0.00175 mmol	5 mol %	0.7 mL	3.10 ± 0.03	3.0 ± 0.2
3	JM 5083	0.00175 mmol	5 mol %	0.7 mL	3.20 ± 0.03	

First order plots were linear to at least 68% conversion.

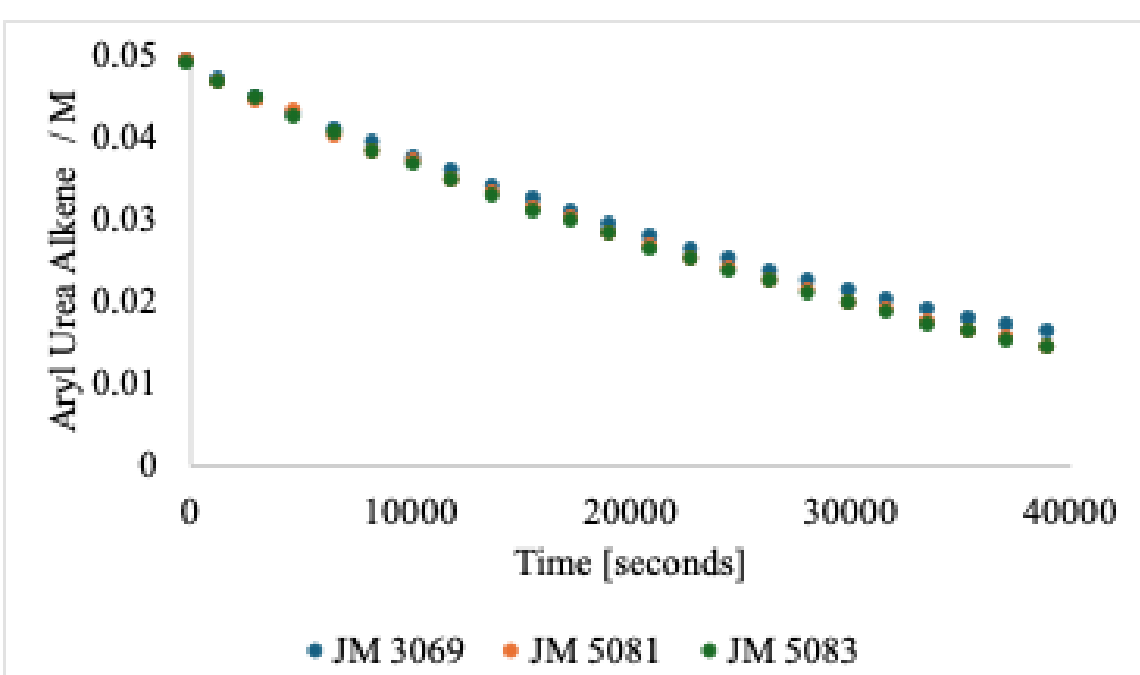

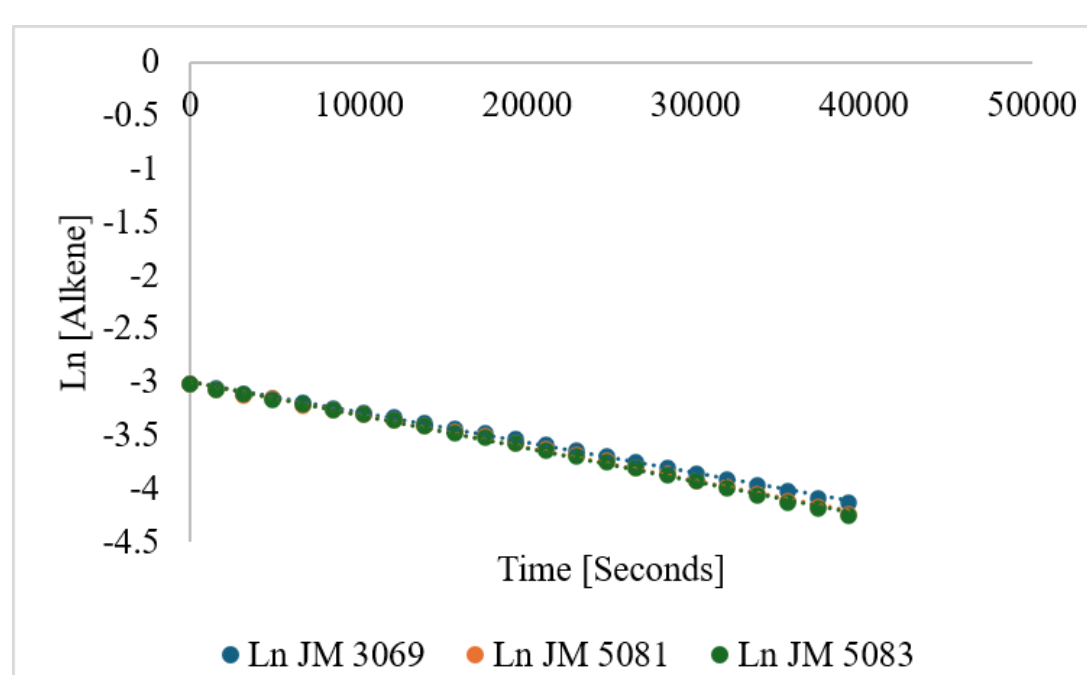

Figure S3. Raw and corresponding first order plots for decay of **7b** (0.05 M) in 0.7 mL CD_2Cl_2 with 5 mol % cat. **A**.

Order in catalyst.

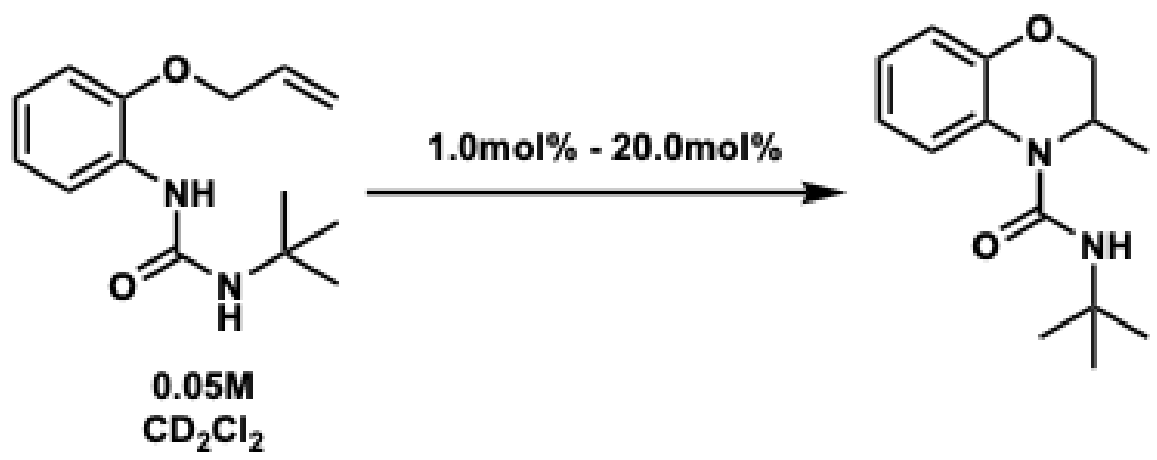

Cat. **A**=

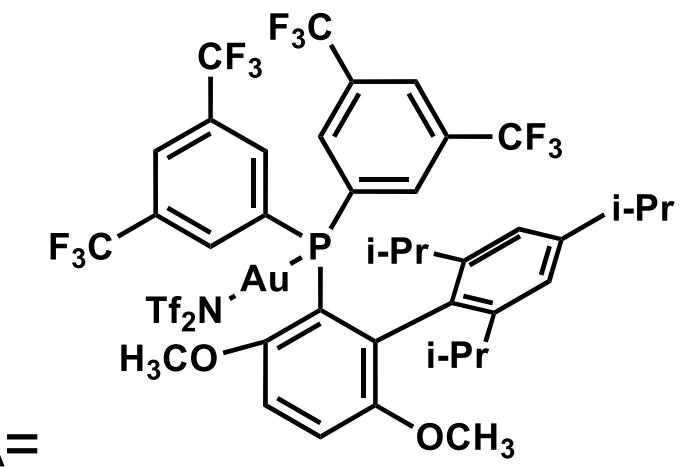

Entry	experiment Ccode	catalyst amount	catalyst loading	CD_2Cl_2	$10^5 \times k_{obs}$ [s^{-1}]
1	JM 5137	0.00035 mmol	1.0 mol %	0.7 mL	0.3 ± 0.004
2	JM 5135	0.000875 mmol	2.5 mol %	0.7 mL	1.1 ± 0.004
3	JM 3039	0.00175 mmol	5.0 mol %	0.7 mL	3.0 ± 0.03
4	JM 3057	0.0035 mmol	10.0 mol %	0.7 mL	4.3 ± 0.03
5	JM 3075	0.007 mmol	20.0 mol %	0.7 mL	8.8 ± 0.07

First order plots were linear to at least 13 % - 98 %conversion (1.0 mol % - 20 mol %).

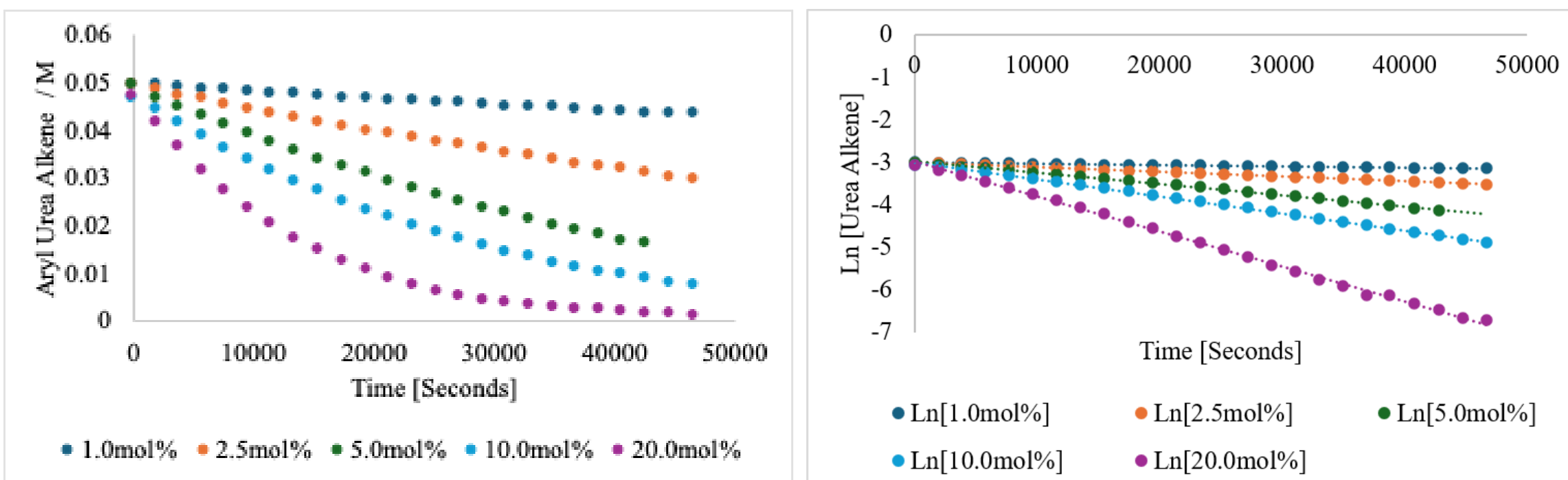

Figure S4. Raw and corresponding first order plots for the decay of **7b** (0.05 M) in 0.7 mL DCM-d_2 with varied catalyst concentration.

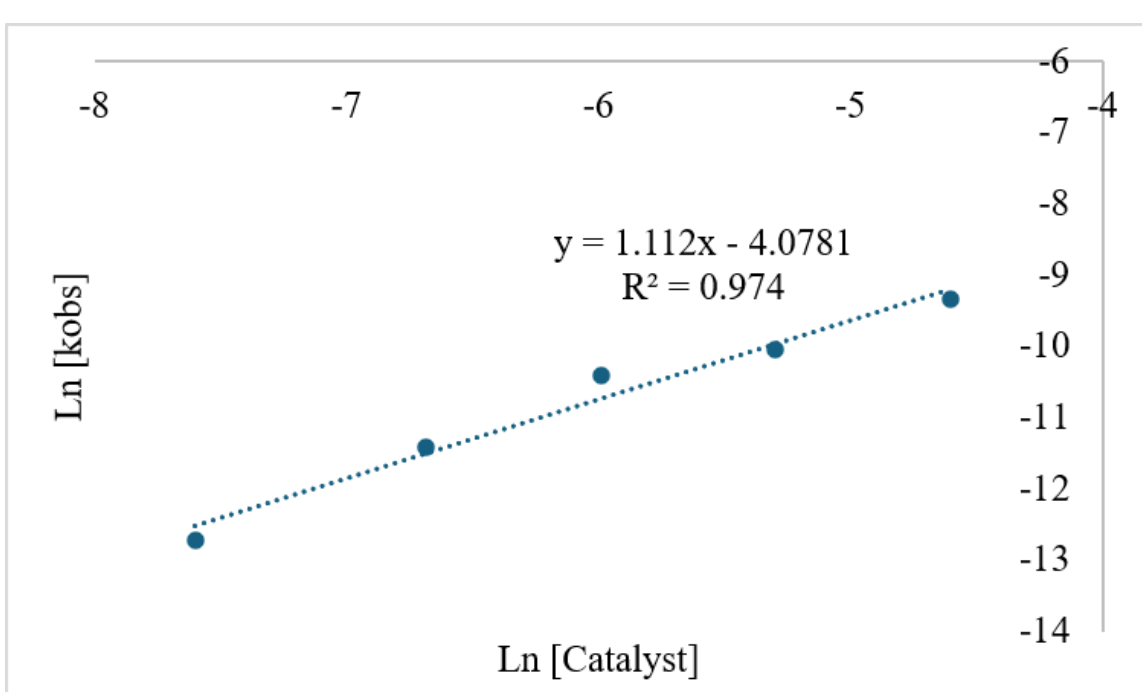

Figure S5. Plot of ln[cat. **A**] vs. ln[k_{obs}] gives a slope of 1 indicating first order in catalyst.

Anion effect.

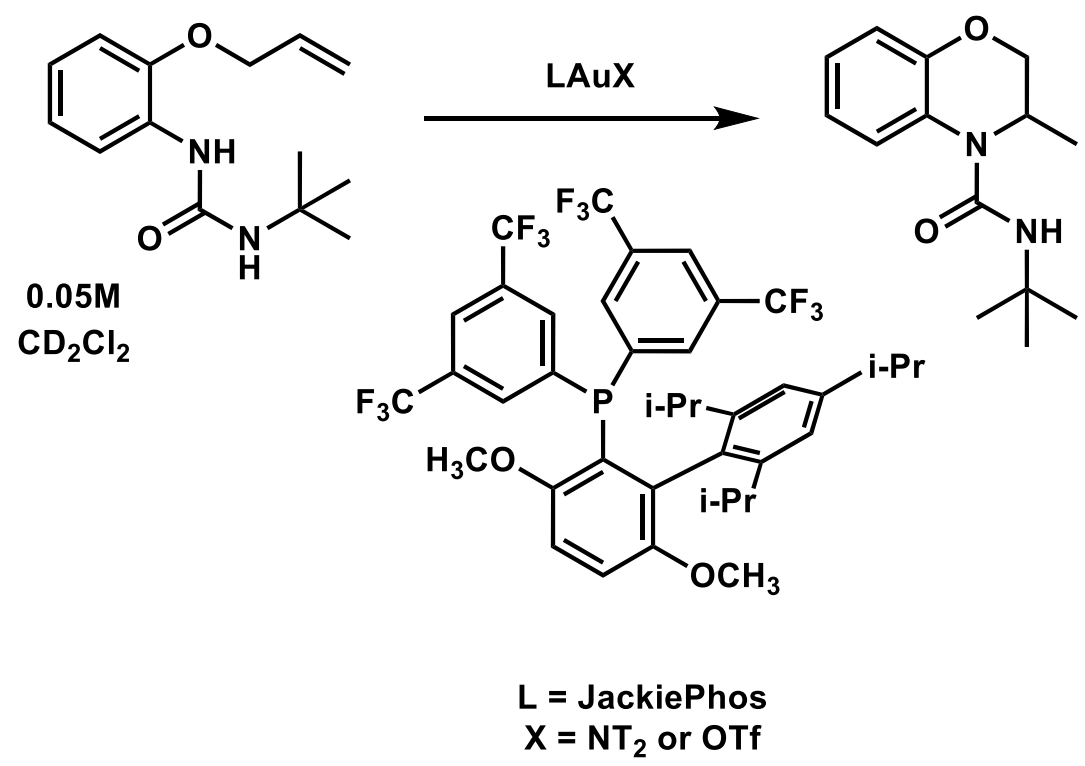

Entry	experiment code	catalyst	X	$10^5 \times k_{obs}$ [s^{-1}]	entry	experiment code	catalyst	X	$10^5 \times k_{obs}$ [s^{-1}]
1	JM 3039	5.0 mol %	NTf_2	3 ± 0.03	4	JM 4053	5.0 mol %	OTf	0.85 ± 0.01
2	JM 3057	10.0 mol %	NTf_2	4.3 ± 0.03	5	JM 4055	10.0 mol %	OTf	2.7 ± 0.03
3	JM 3075	20.0 mol %	NTf_2	8.8 ± 0.07	6	JM 4057	20.0 mol %	OTf	6.3 ± 0.07

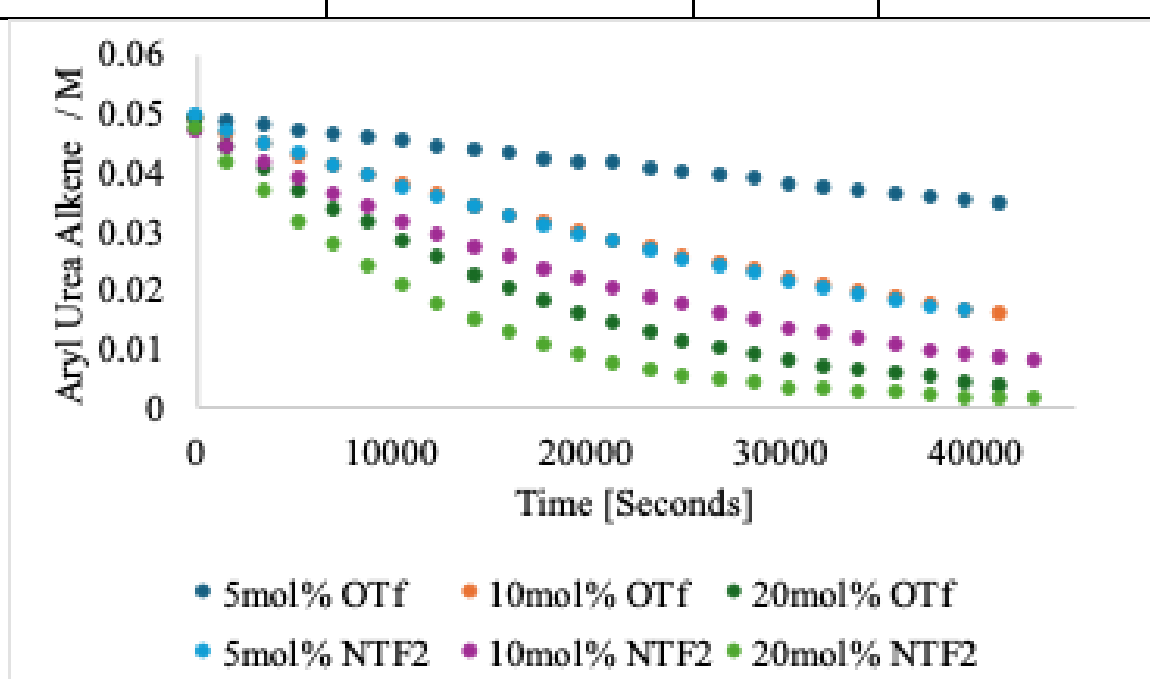

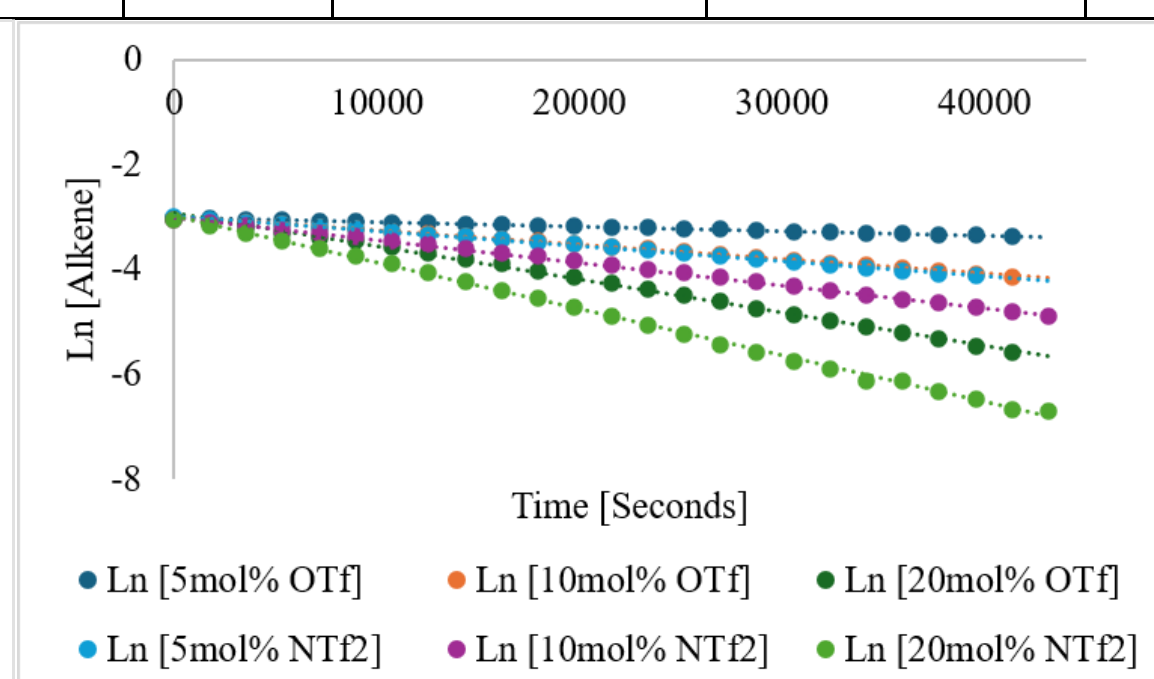

Figure S6. Raw and corresponding first order plots for the decay of **7b** (0.05 M) in 0.7 mL of DCM-d_2 with varied catalyst loading. First order plots were monitored and linear up to 30% at 5.0 mol % catalyst loading and to completion at 20.0 mol % catalyst loading.

Ligand effect.

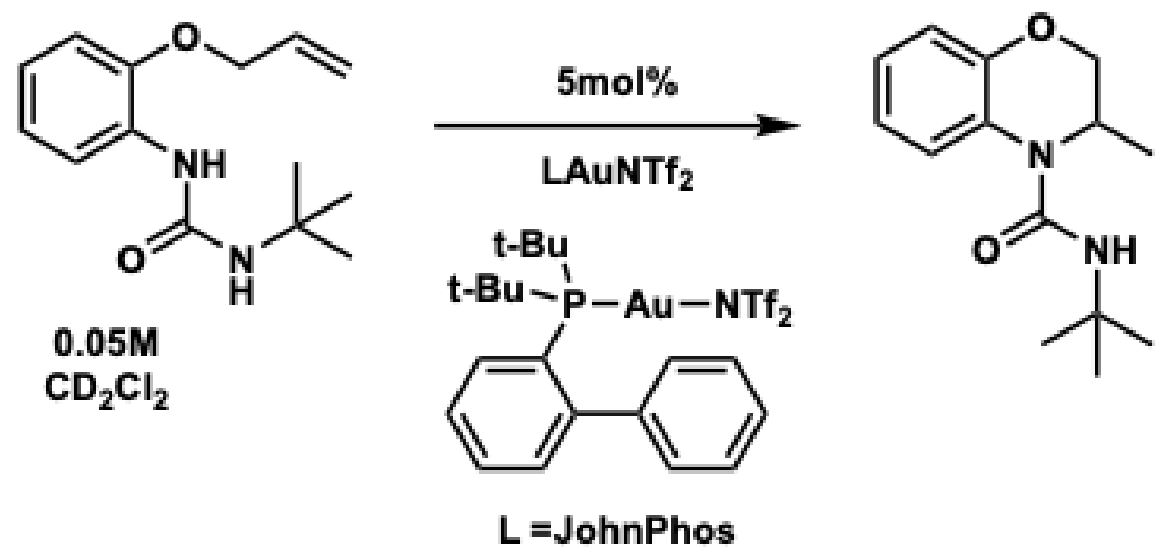

Entry	experiment code	catalyst amount	catalyst loading	$10^5 \times k_{obs}$ [s^{-1}]	average
1	JM 5013	0.00175 mmol	5 mol %	0.13 ± 0.004	0.095 ± 0.03
2	JM 5145	0.00175 mmol	5 mol %	0.074 ± 0.002	
3	JM 5147	0.00175 mmol	5 mol %	0.082 ± 0.002	

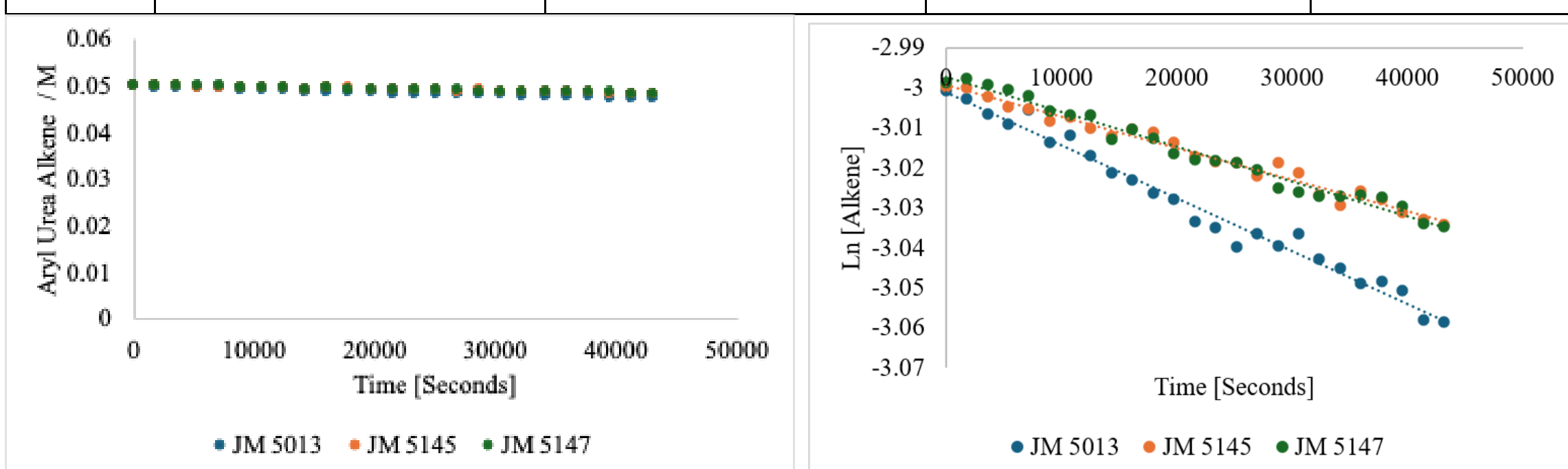

Figure S7. Raw and corresponding first order plots for the decay of **7b** (0.05 M) in 0.7 mL of DCM-d_2 with JPhosAuNTf$_2$ (5 mol %). First order plots were monitored and linear up to 7% conversion.

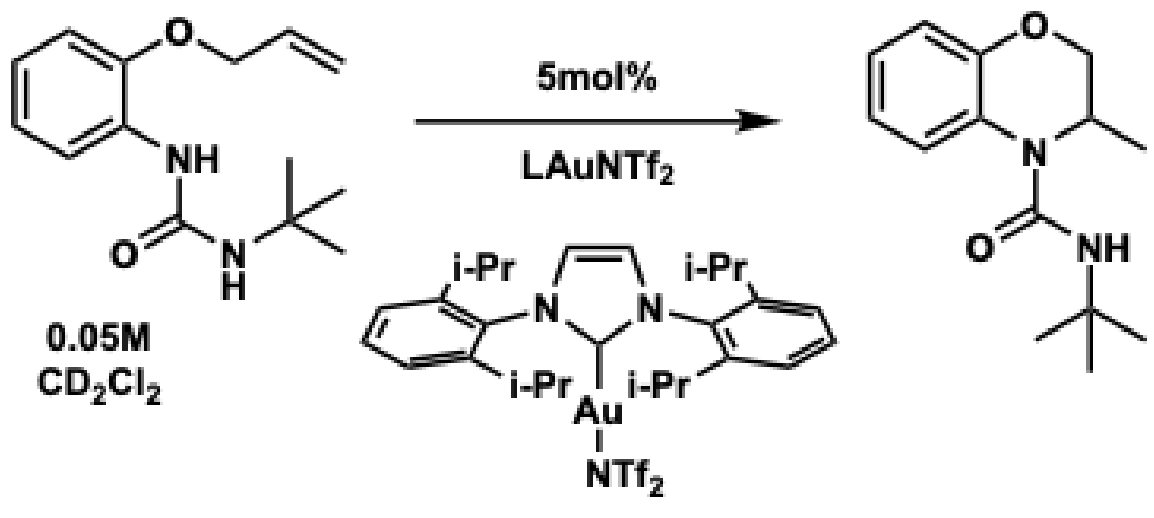

Entry	experiment code	catalyst amount	catalyst loading	$10^5 \times k_{obs}$ [s^{-1}]	average
1	JM 5065	0.00175 mmol	5 mol %	0.052 ± 0.002	0.06 ± 0.01
2	JM 5069	0.00175 mmol	5 mol %	0.072 ± 0.004	
3	JM 5071	0.00175 mmol	5 mol %	0.058 ± 0.002	

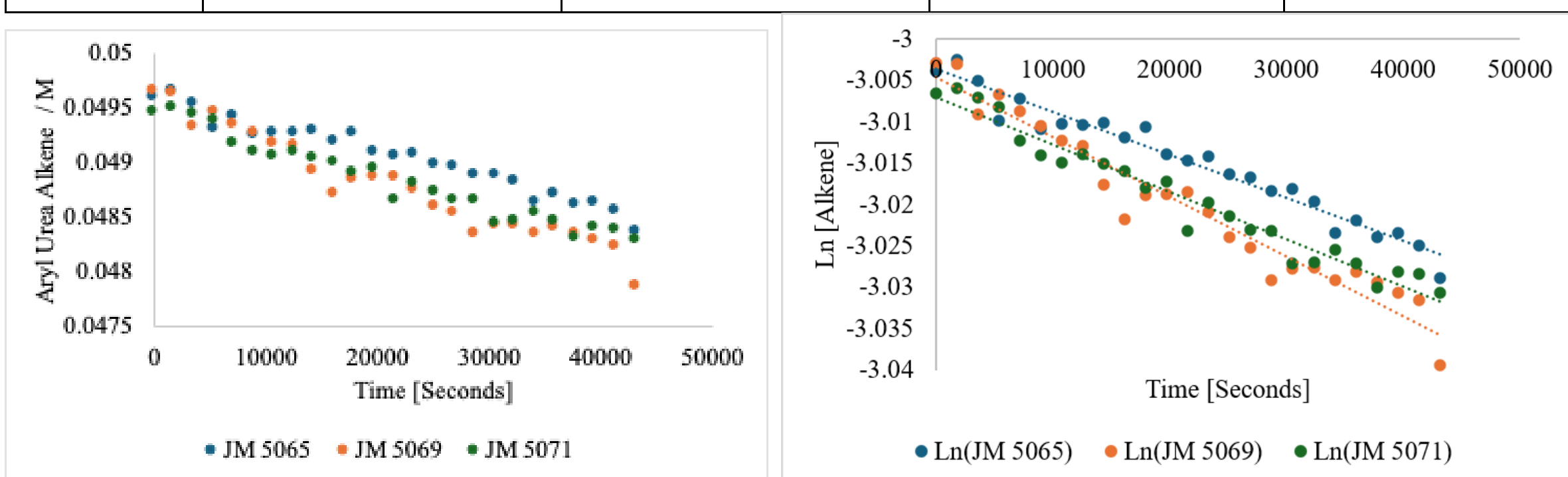

Figure S8. Raw and corresponding first order plots for the decay of **7b** (0.05 M) in 0.7 mL of DCM-d_2 with IPrAuNTf$_2$. First order plots were monitored and linear to 3% conversion.

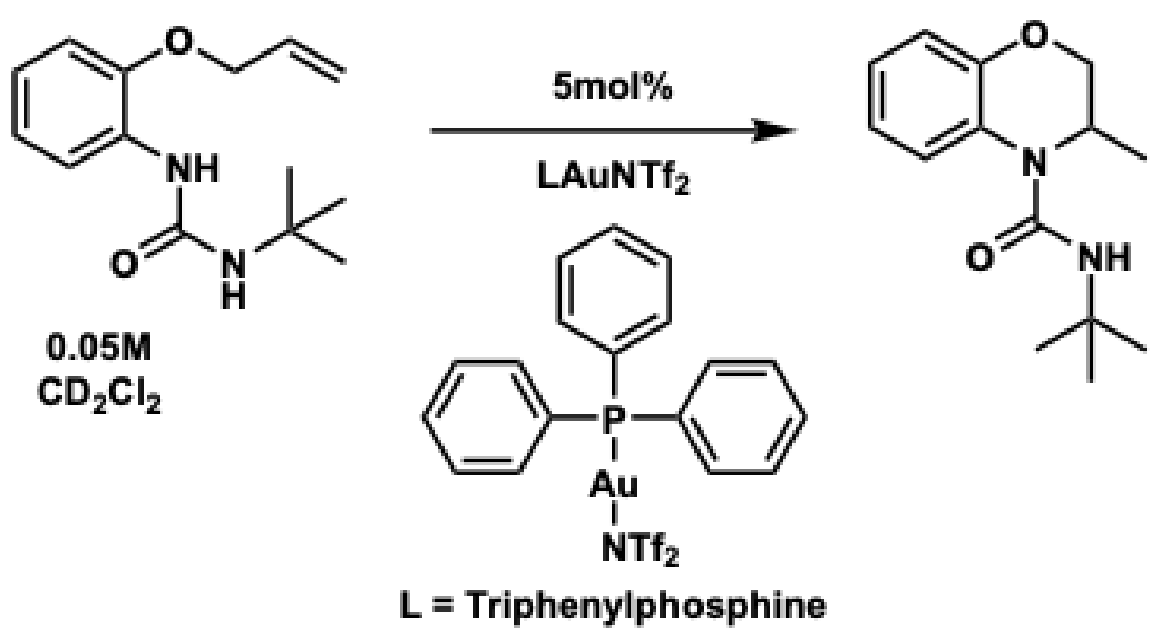

Entry	experiment code	catalyst amount	catalyst loading	$10^5 \times k_{obs}$ [s^{-1}]	average
1	JM 3077	0.00175 mmol	5 mol %	0.025 ± 0.001	0.043 ± 0.016
2	JM 6013	0.00175 mmol	5 mol %	0.050 ± 0.001	
3	JM 6015	0.00175 mmol	5 mol %	0.056 ± 0.001	

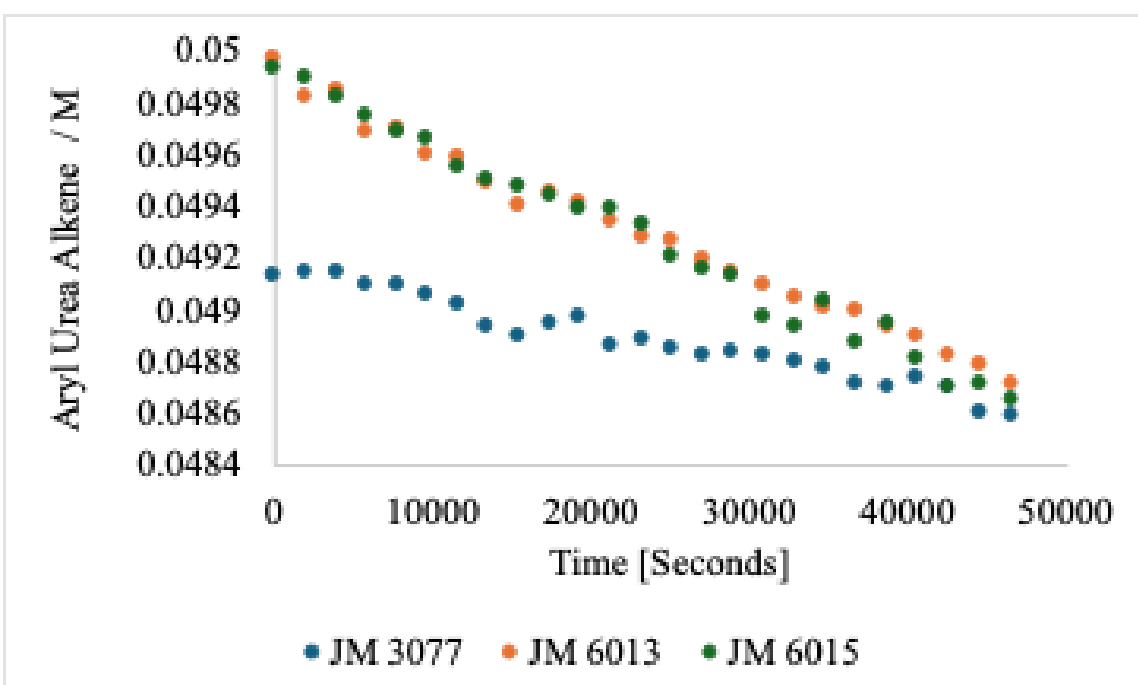

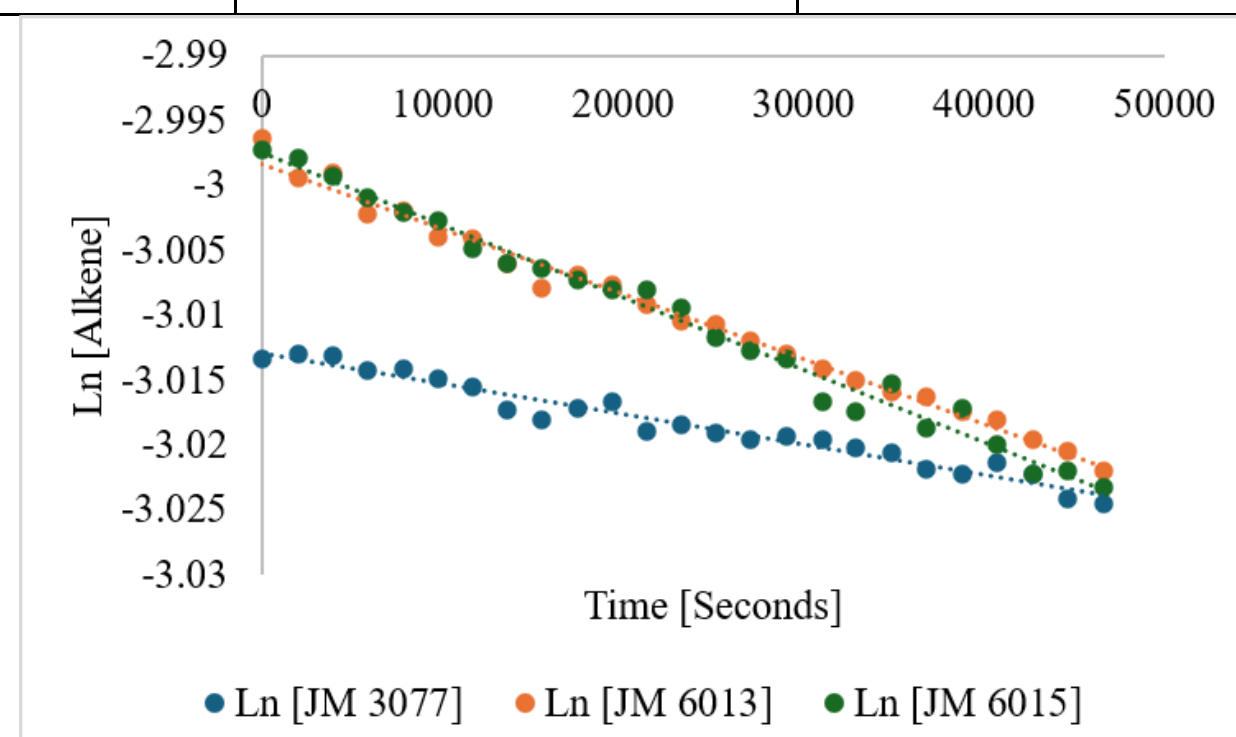

Figure S9. Raw and corresponding first order plots for the decay of **7b** (0.05 M) in 0.7 mL of CD_2Cl_2 with $Ph_3PAuNTf_2$·1/2toluene. First order plots were monitored and linear to 3% conversion.

Ligand effect summary.

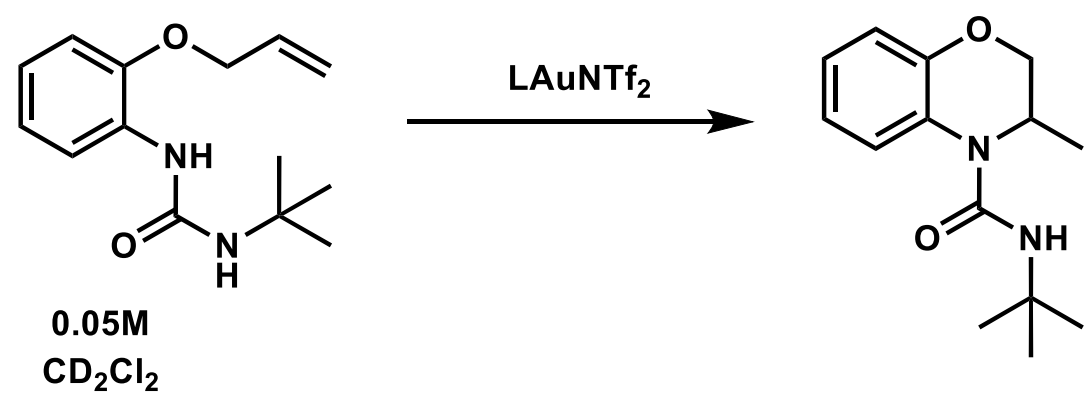

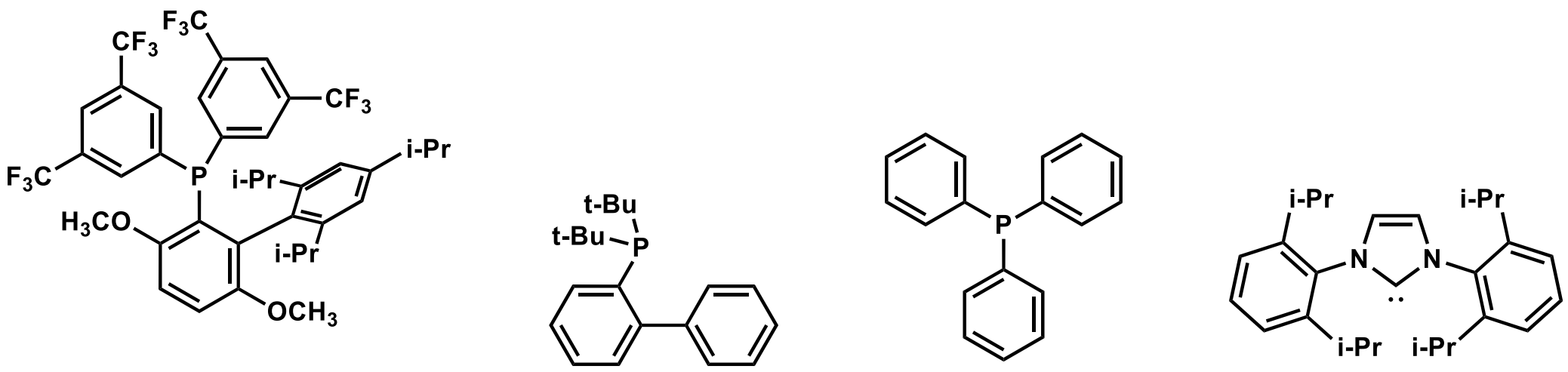

JackiePhos JohnPhos Triphenylphosphine IPr

**Ligand**	**catalyst amount**	**catalyst loading**	**$10^5 \times k_{obs}$ [s^{-1}]**
Triphenylphosphine	0.00175 mmol	5 mol %	0.043 ± 0.016
IPr	0.00175 mmol	5 mol %	0.06 ± 0.03
JohnPhos	0.00175 mmol	5 mol %	0.095 ± 0.03
JackiePhos	0.00175 mmol	5 mol %	3 ± 0.2

Figure S10. Compiled rate constants (each average of 3 trials).

Kinetics with 1-(*tert*-butyl)-3-(pent-4-en-1-yl)urea (**12**)

Ligand effect

Same representative procedure as that with 1-(2-(allyloxy)phenyl)-3-(*tert*-butyl)urea (**7b**).

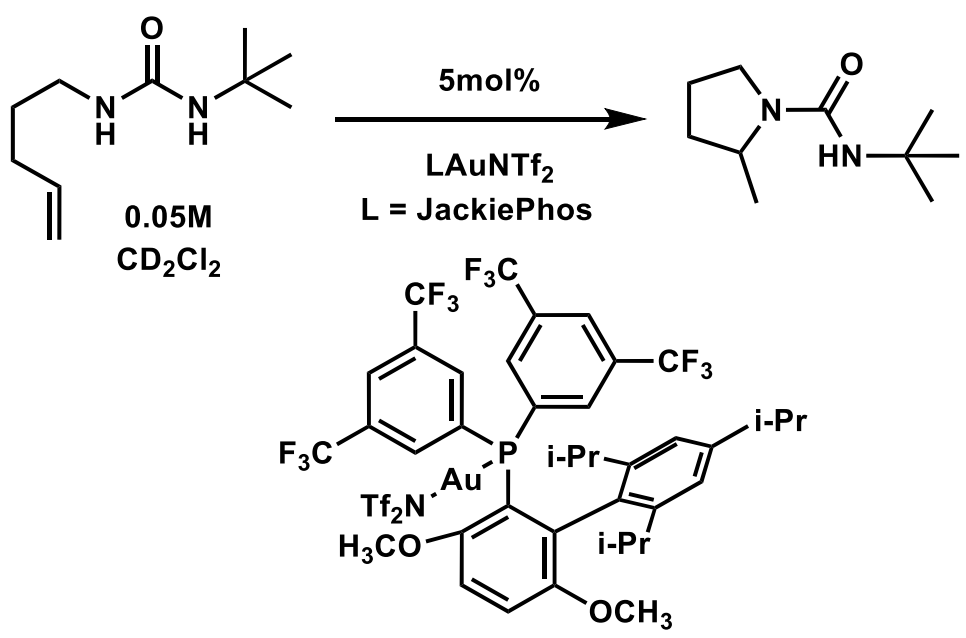

Entry	experiment code	catalyst amount	catalyst loading	CD_2Cl_2	$10^5 \times k_{obs}$ [s^{-1}]	
1	JM 5073	0.00175 mmol	5 mol %	0.7 mL	44 ± 1.3	Average:
2	JM 5087	0.00175 mmol	5 mol %	0.7 mL	34 ± 3.1	39 ± 5
3	JM 5089	0.00175 mmol	5 mol %	0.7 mL	38 ± 0.97	

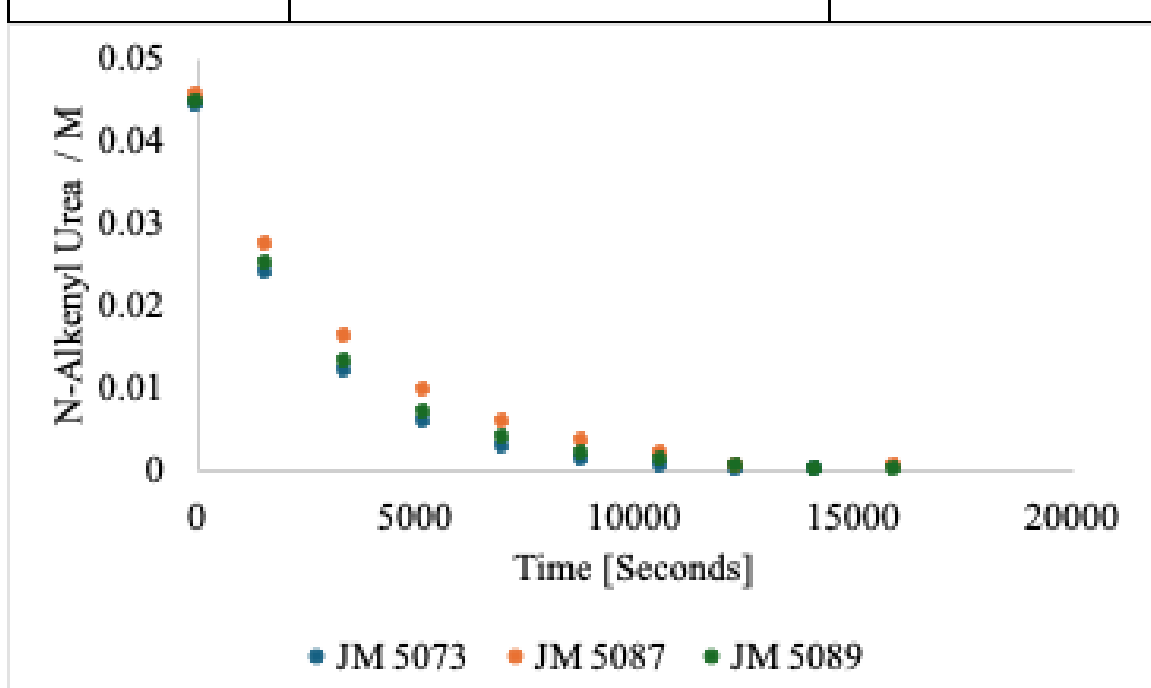

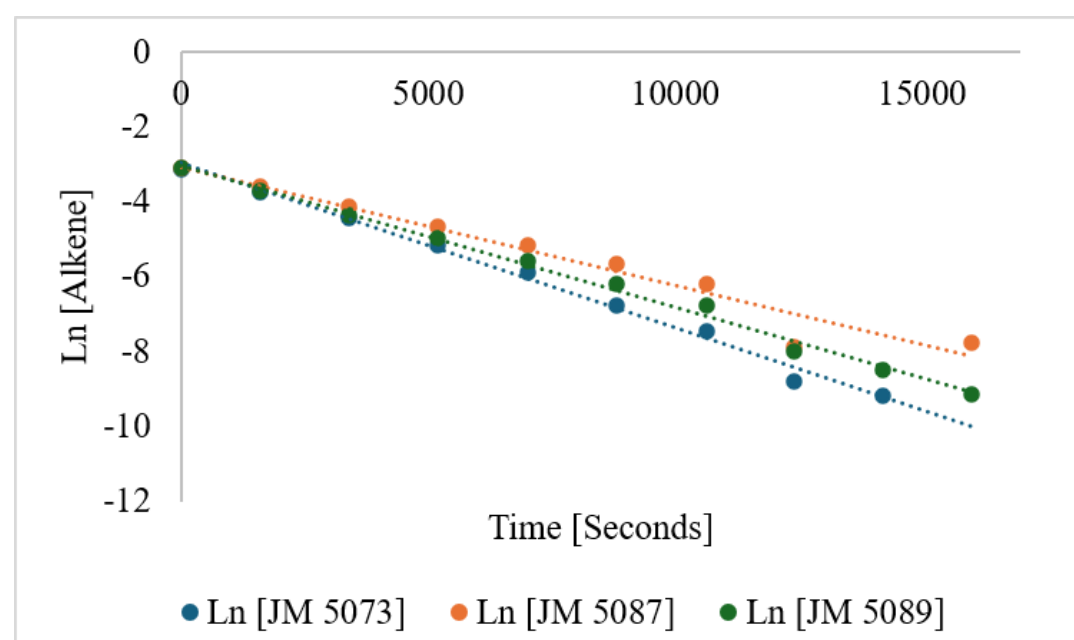

Figure S11. Raw and corresponding first order plots for the decay of **12** (0.05 M) in 0.7 mL of DCM-d_2 with 0.00175 mmol (5 mol %) cat. **A**. First order plots were linear to completion.

Entry	experiment code	catalyst amount	catalyst loading	CD_2Cl_2	$10^5 \times k_{obs}$ [s^{-1}]
1	JM 5103	0.00035 mmol	1.0 mol %	0.7 mL	6 ± 0.12
2	JM 5097	0.000875 mmol	2.5 mol %	0.7 mL	16 ± 0.14

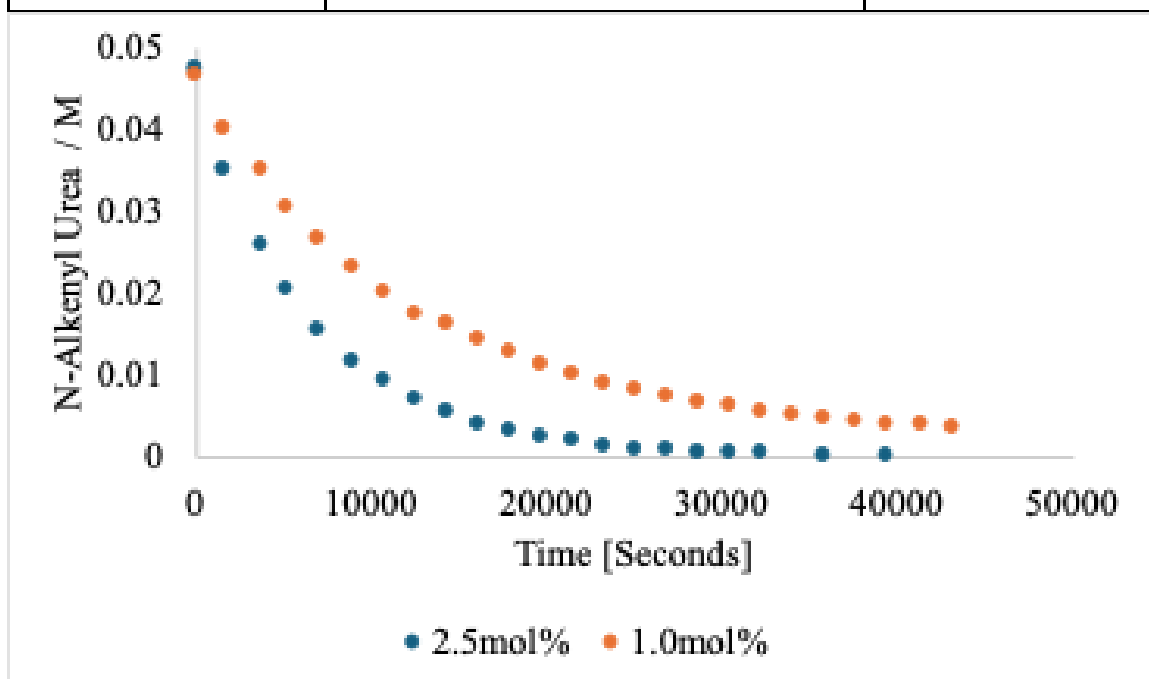

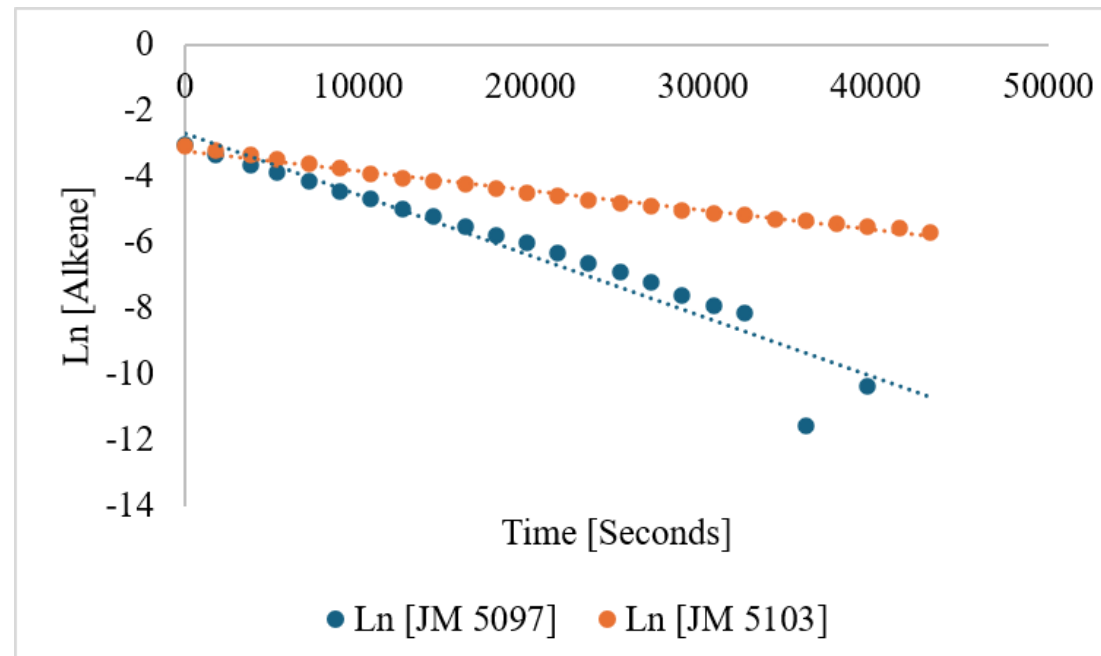

Figure S12. Raw and corresponding first order plots for the decay of **12** (0.05 M) in 0.7 mL of DCM-d_2 with 1.0 mol % and 2.5 mol % cat. **A**. First order plots were linear to 96% conversion.

Entry	Experiment Code	catalyst amount	catalyst loading	CD_2Cl_2	$10^5 \times k_{obs}$ [s^{-1}]
1	JM 5103	0.00035 mmol	1.0 mol %	0.7 mL	6 ± 0.12
2	JM 5097	0.000875 mmol	2.5 mol %	0.7 mL	16 ± 0.14
3	JM 5089	0.00175 mmol	5 mol %	0.7 mL	39 ± 5*

*The rate is reported as an average of 3 trials (see above).

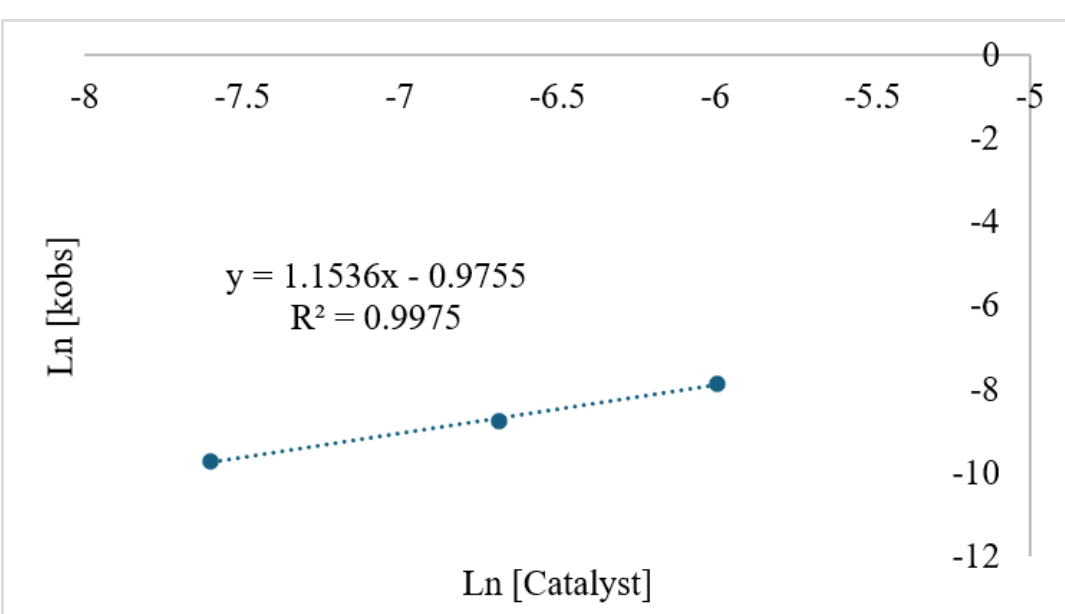

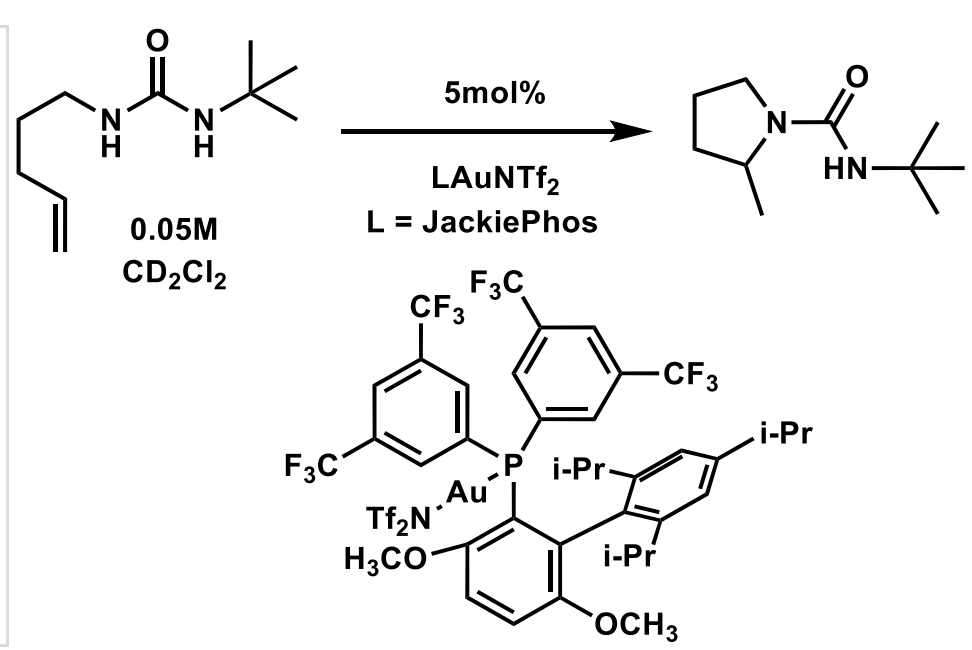

Figure S13. Ln[cat. **A**] vs. ln[k_{obs}] gave a slope of 1.2.

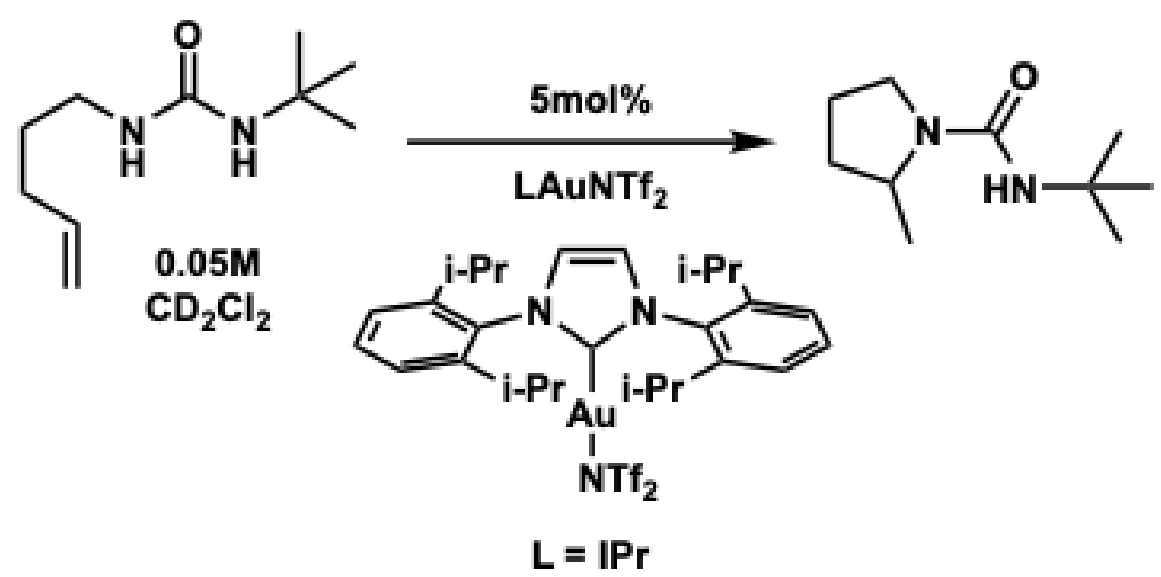

Entry	experiment code	catalyst amount	catalyst loading	CD_2Cl_2	$10^5 \times k_{obs}$ [s^{-1}]	
1	JM 5075	0.00175 mmol	5 mol %	0.7 mL	1.91 ± 0.02	Average:
2	JM 5077	0.00175 mmol	5 mol %	0.7 mL	1.95 ± 0.02	1.95 ±
3	JM 5079	0.00175 mmol	5 mol %	0.7 mL	1.98 ± 0.02	0.04

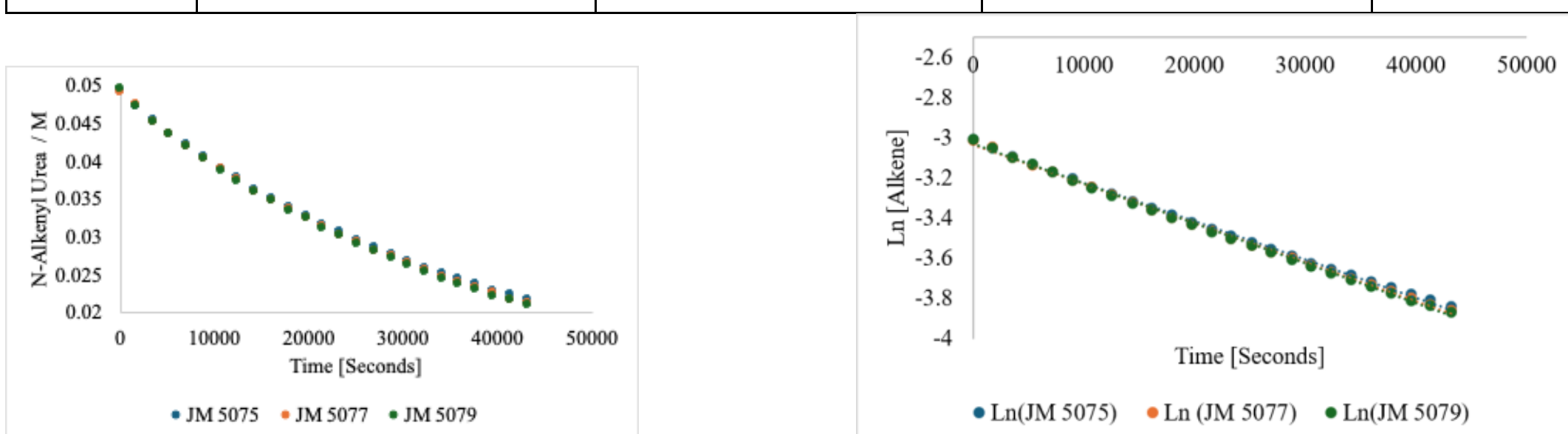

Figure S14. Raw and corresponding first order plots for the decay of **12** (0.05 M) in 0.7 mL of DCM-d_2 with 0.00175 mmol (5 mol %) $IPrAuNTf_2$. First order plots were linear to 58% conversion.

Entry	experiment code	catalyst amount	catalyst loading	CD_2Cl_2	$10^5 \times k_{obs}$ [s^{-1}]
1	JM 5101	0.00035 mmol	1.0 mol %	0.7 mL	0.5 ± 0.02
2	JM 5099	0.000875 mmol	2.5 mol %	0.7 mL	1.4 ± 0.01

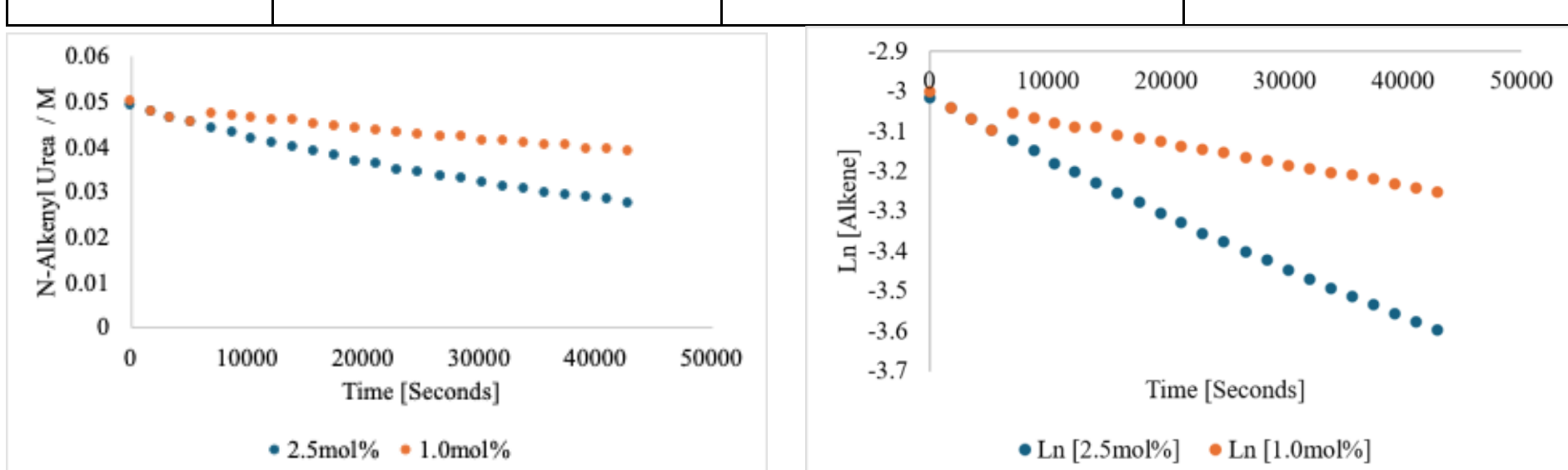

Figure S15. Raw and corresponding first order plots for the decay of **12** (0.05 M) in 0.7 mL of DCM-d_2 with 0.000875 mmol and 0.00035 mmol (2.5 mol % / 1.0 mol %) $IPrAuNTf_2$. First order plots were linear to 22% conversion.

Entry	experiment code	catalyst amount	catalyst loading	CD_2Cl_2	$10^5 \times k_{obs}$ [s^{-1}]
1	JM 5101	0.00035 mmol	1.0 mol %	0.7 mL	0.5 ± 0.02
2	JM 5099	0.000875 mmol	2.5 mol %	0.7 mL	1.4 ± 0.01
3	JM 5079	0.00175 mmol	5.0 mol %	0.7 mL	1.95 ± 0.04*

*The rate is reported as an average of 3 trials (see above).

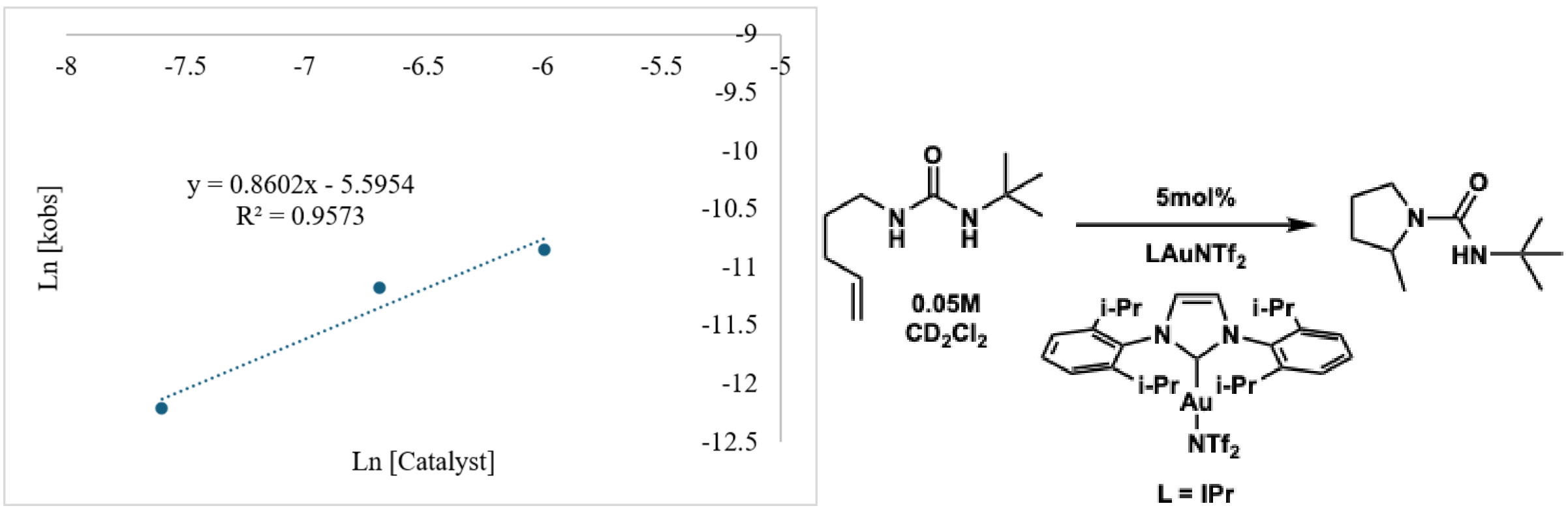

Figure S16. Ln[$IPrAuNTf_2$] vs. Ln[k_{obs}] gave a slope of 0.9.

Control studies

Vial Reaction Screen

Reactivity screens were performed with 4-methyl-*N*-(2-((2-methylallyl)oxy)phenyl)benzenesulfonamide (**4a**) or 1-(2-(allyloxy)phenyl)-3-(*tert*-butyl)urea (**7b**) under a variety of catalyst conditions. The substrate (0.10 mmol, 1 equiv) was weighed into a scintillation vial. The catalyst (0.005 mmol, 5 mol %) was subsequently weighed into the same vial and 1.0 mL of 1,2-dichloroethane was added. The vial was sealed with a screw cap wrapped with parafilm, and placed in a heated reaction block at 75 °C and stirred overnight. DCE was removed under reduced pressure. The reaction mixture was redissolved in $CDCl_3$ for analysis by NMR spectroscopy.

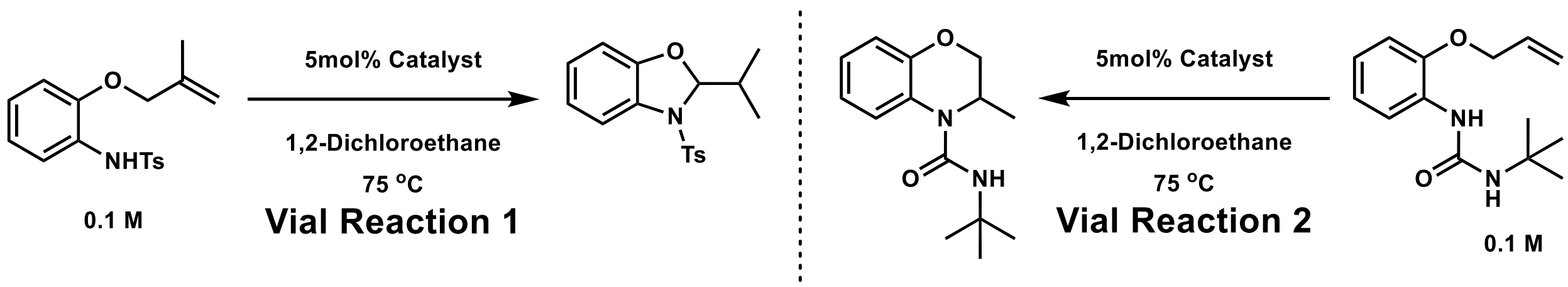

L1=JackiePhos L2=JPhos L3=Ph_3P L4=IPr

Table S1. Reactivity screen for 4-methyl-*N*-(2-((2-methylallyl)oxy)phenyl) benzenesulfonamide (**4a**) and 1-(2-(allyloxy)phenyl)-3-(*tert*-butyl)urea (**7b**)

**Entry**	**Catalyst**	**Vial Reaction 1**[1]			**Vial Reaction 2**[1]		
		Page #	Reactivity[2]/*t*	^{31}P (δ / ppm)	Page #	Reactivity[2]/*t*	^{31}P (δ / ppm)
1	Cat. **A**	JM 2103	I, 20 h	18	JM 2127	Y, 24 h	18
2	L1AuOTf	JM 4077	N, 24 h	18	JM 4143	Y, 24 h	18
3	L2AuNTf$_2$	JM 5149	M, 24 h	57	JM 5139	Y, 24 h	60/58
4	L3AuNTf$_2$	JM 3107	Y, 36 h	45	JM 5155	M, 24 h	30
5	Cat. **B**	JM 2061	N, 22 h	NA	JM 2011	Y, 18 h	60/58
6	L4AuNTf$_2$	JM 5151	N, 24 h	NA	JM 5153	M, 24 h	NA
7	L1AuCl	JM 3059	N, 67 h	18	JM 6007	N, 24 h	18
8	L1AuCl + HOTf	JM 5109	Y, 24 h	18	JM 6009	N, 24 h	18
9	HOTf	JM 4079	Y, 24 h	NA	JM 4081	N, 24 h	NA
10	L1AuCl generated from	JM 5093	M, 24 h	18	JM 5095	N, 24 h	18

	L1AuOTf in DCE at 75 °C[3]						
11	AgOTf	JM 2063	Y, 20 h	NA	JM 6011	N, 24 h	NA

[1]For reaction 1, unreacted starting material and/or cleaved allyl ether remain. For reaction 2, unreacted starting material remains. [2]Reactivity defined as yes (Y) = >10% conversion, marginal (M)= <10% conversion, Intermittent (I) = results difficult to reproduce, No (N) = No conversion to desired product. [3]L1AuOTf was heated in DCE at 75 °C for 24 h. Substrate was then added in one portion and allowed to react for 24 hours.

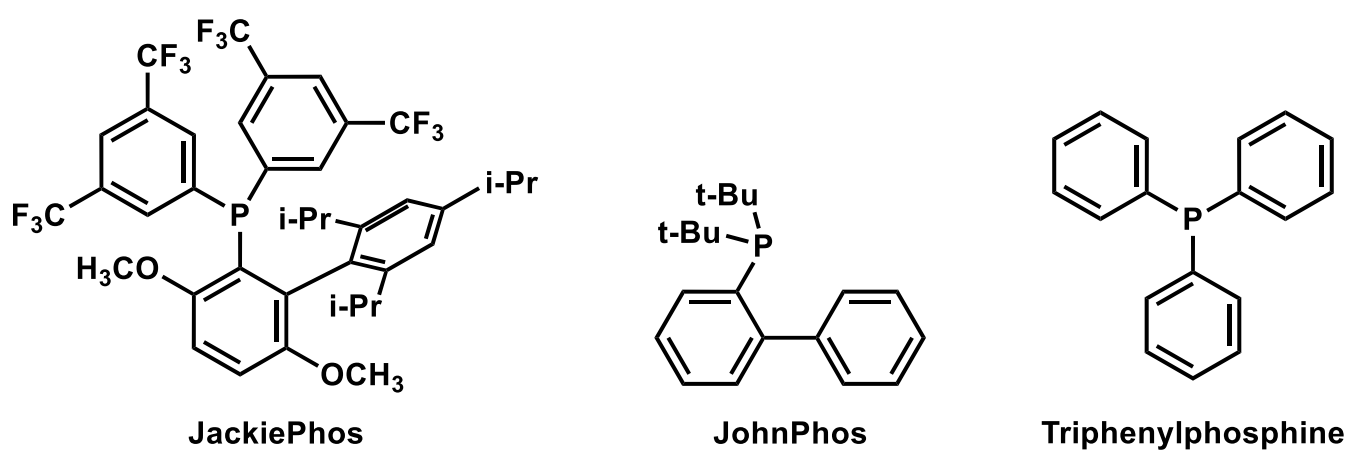

Table S2 Summary of ^{31}P NMR chemical shifts for species observed during vial reactions.

Catalyst	Catalyst	Decay observed
cat. **A**	14 ppm	18 ppm (JackiePhosAuCl)
JackiePhosAuOTf	12 ppm	18 ppm (JackiePhosAuCl)
JPhosAuNTf$_2$	58 ppm	60 ppm (JPhosAuCl)
Ph$_3$PAuNTf$_2$	30 ppm	45 ppm (Ph$_3$P)$_2$Au$^+$
cat. **B**	58 ppm	60 ppm (JphosAuCl)

Generation of JackiePhosAuCl during reaction kinetics

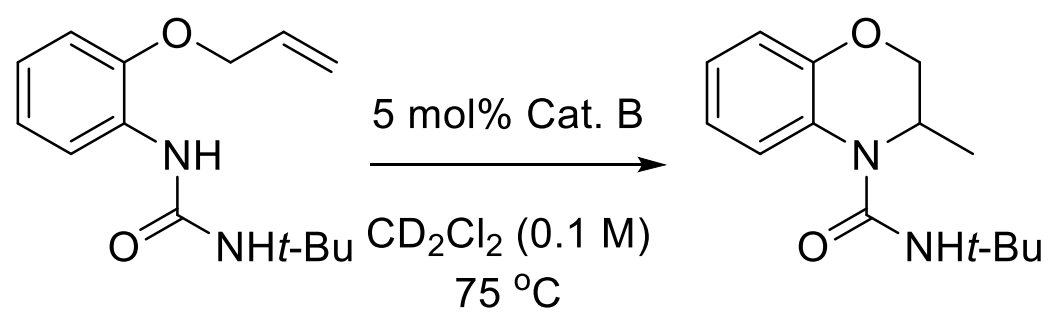

30% Conversion at t=21 Hours

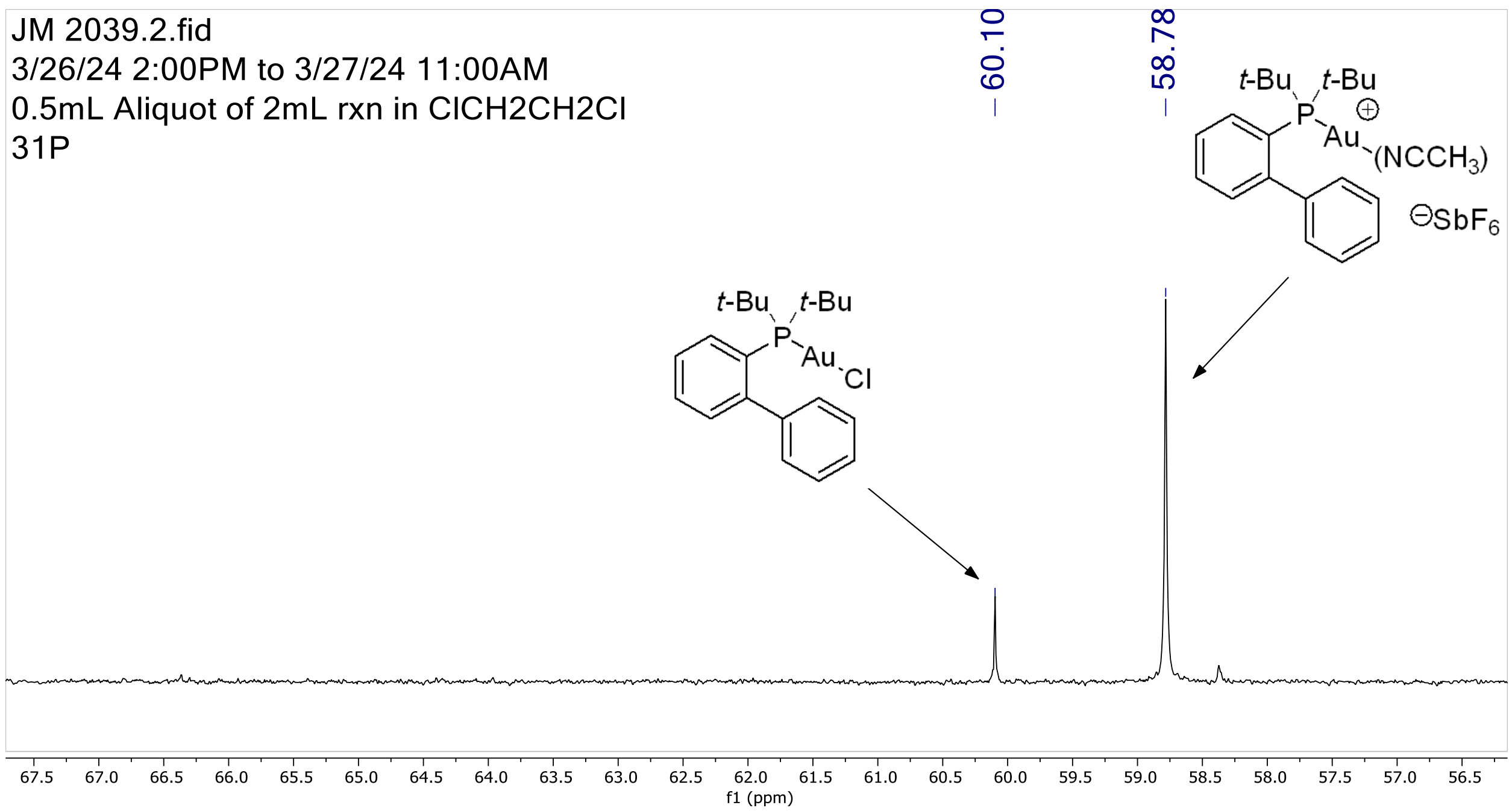

Figure S17. Spectra of catalyst during hydroamination of 1-(2-(allyloxy)phenyl)-3-(*tert*-butyl)urea (**7b**) showing significant amount of remaining JPhosAu($NCCH_3$)SbF_6. (cat. **B**) (*t* = 21 hours).

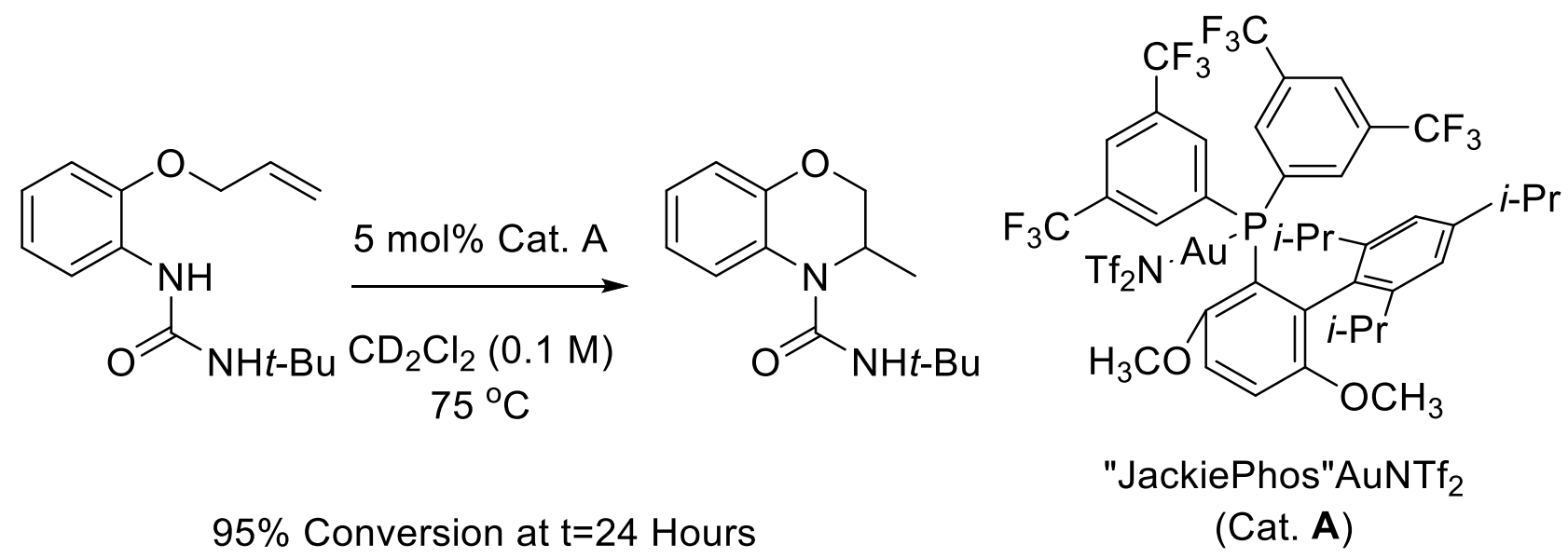

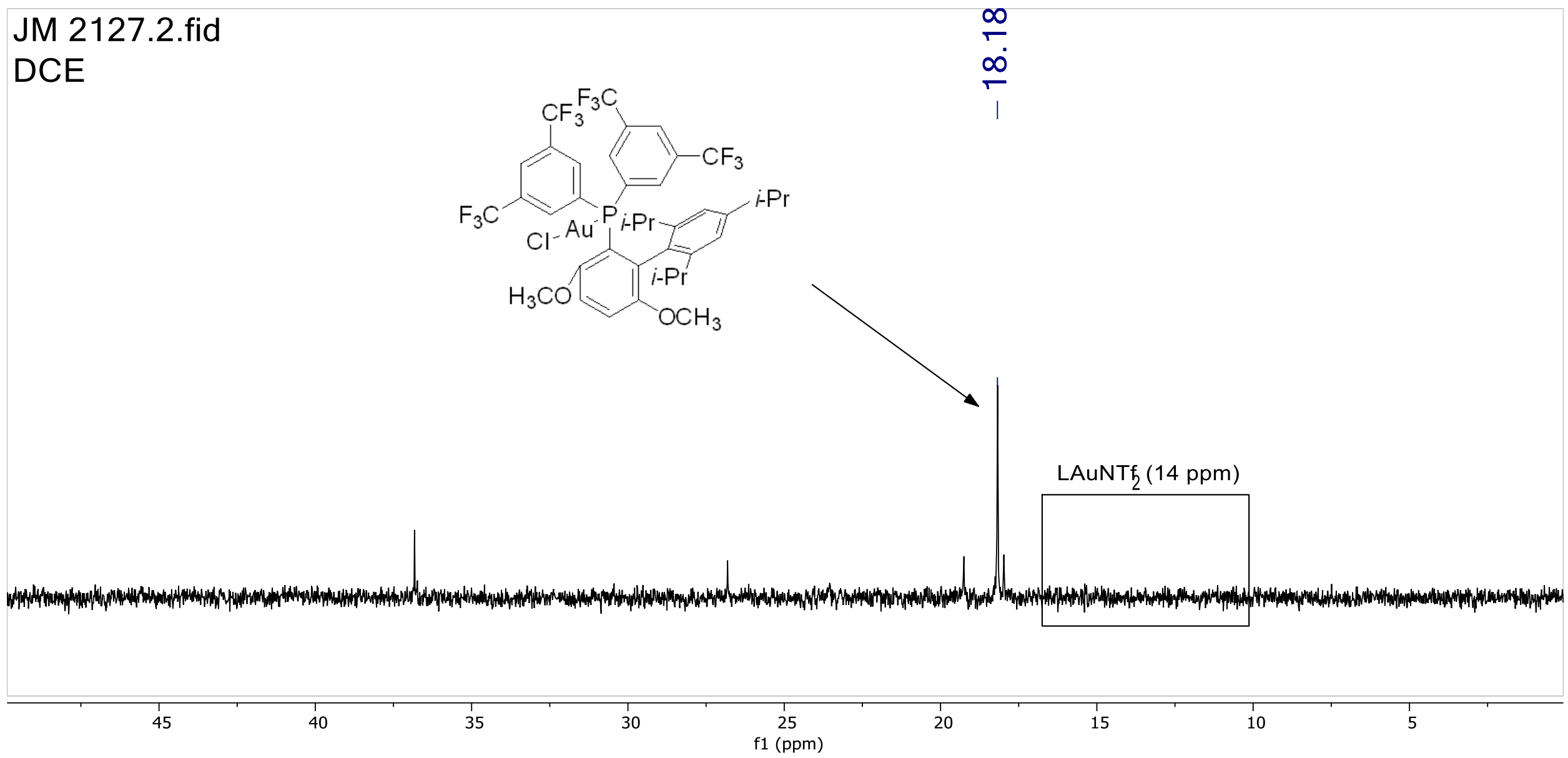

Figure S18. Spectra of catalyst during hydroamination of 1-(2-(allyloxy)phenyl)-3-(*tert*-butyl)urea (**7b**) showing significant degradation to JackiePhosAuCl. (minimal remaining JackiePhosAuNTf$_2$ (cat. **A**) (t = 24 hours).

Regeneration of JackiePhosAuCl from JackiePhosAuX in DCE at 75 °C:

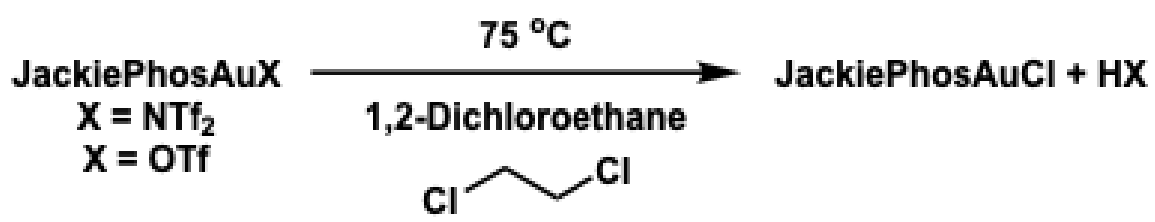

JackiePhosAuX (0.005 mmol, 1 equiv) was added to a scintillation vial at typical catalyst loading for vial reactions. The catalyst was dissolved in DCE (1 mL, 0.1 M) and allowed to heat at 75 °C. ^{1}H, ^{31}P, and ^{19}F NMR spectra were collected at 3, 24, and 48 hours to examine formation of JackiePhosAuCl and HX.

Table S3. ^{31}P NMR chemical shifts for solutions of JackiePhosAuX in DCE after heating at 75 °C at t = 3, 24, 48 hours.

**Entry**	**experiment code**	**X**	**3 hours**	**24 hours**	**48 hours**
1	JM 3127	NTf_2	12 ppm	18 ppm	18 ppm
2	JM 5047	OTf	18 ppm	18 ppm	18 ppm

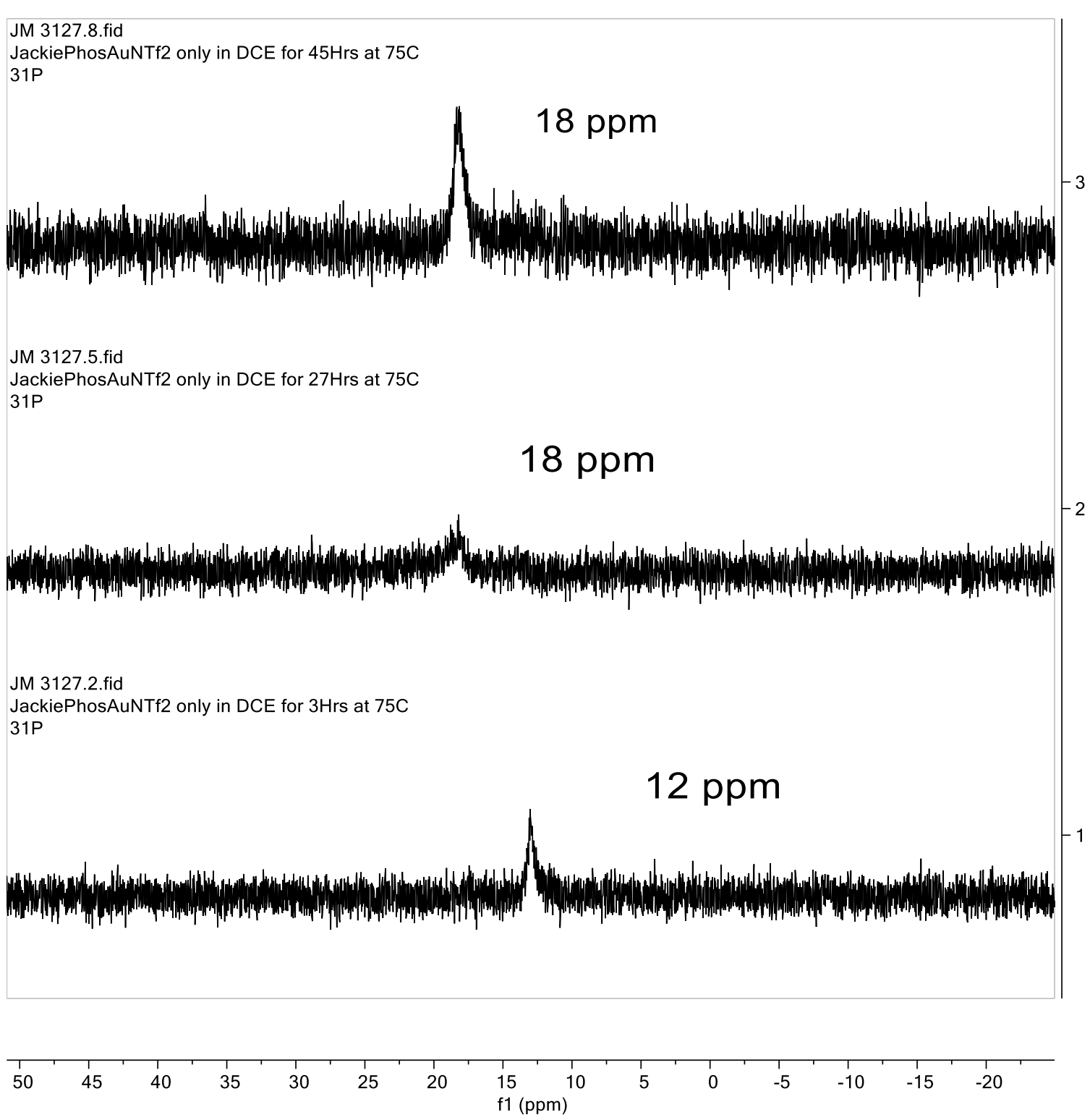

Figure S19. ^{31}P NMR spectra showing formation over JackiePhosAuCl over time from JackiePhosAuNTf_2 (cat. **A**) and DCE.

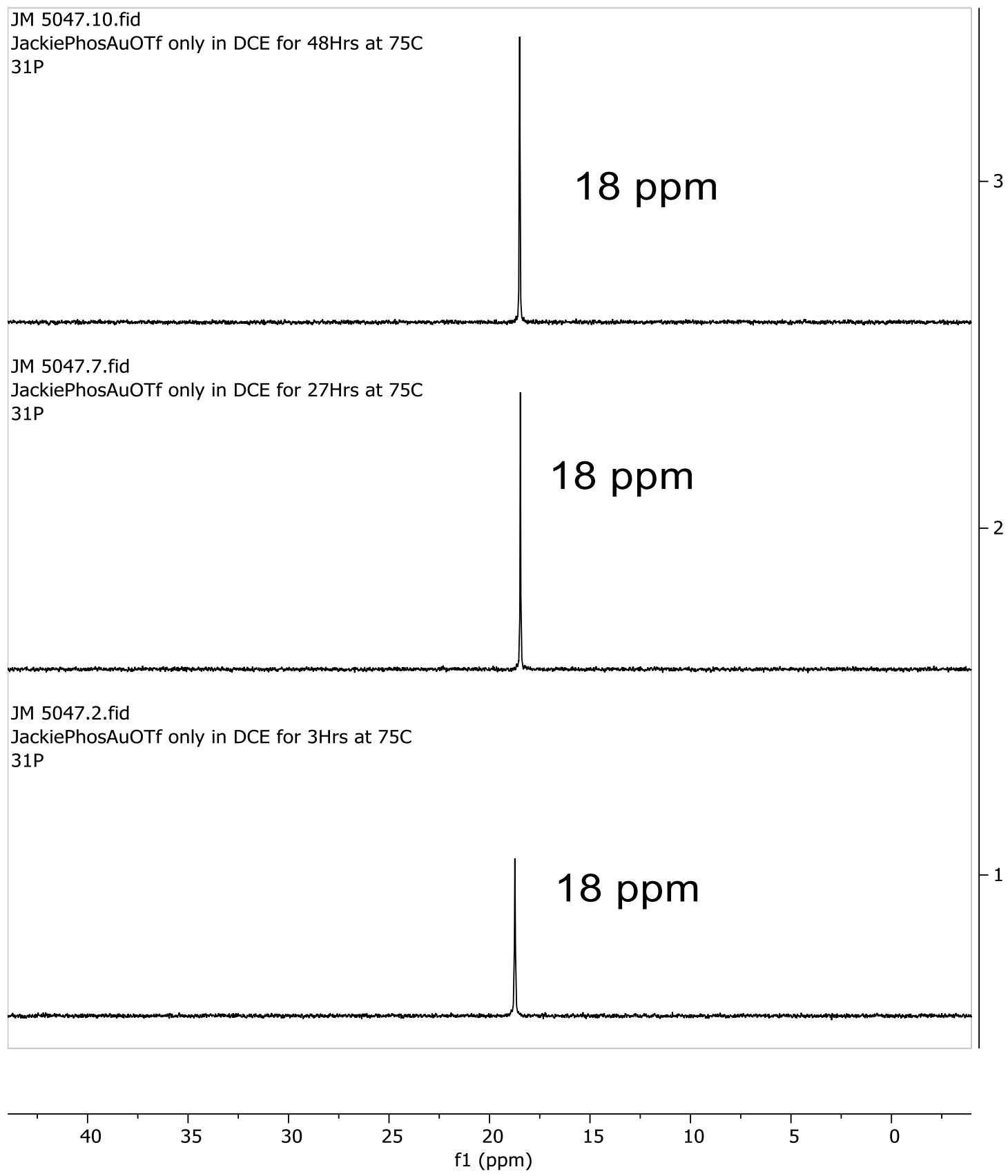

Figure S20. ^{31}P NMR Spectra showing formation of JackiePhosAuCl from JackiePhosAuOTf and DCE.

Thiourea reactivity.

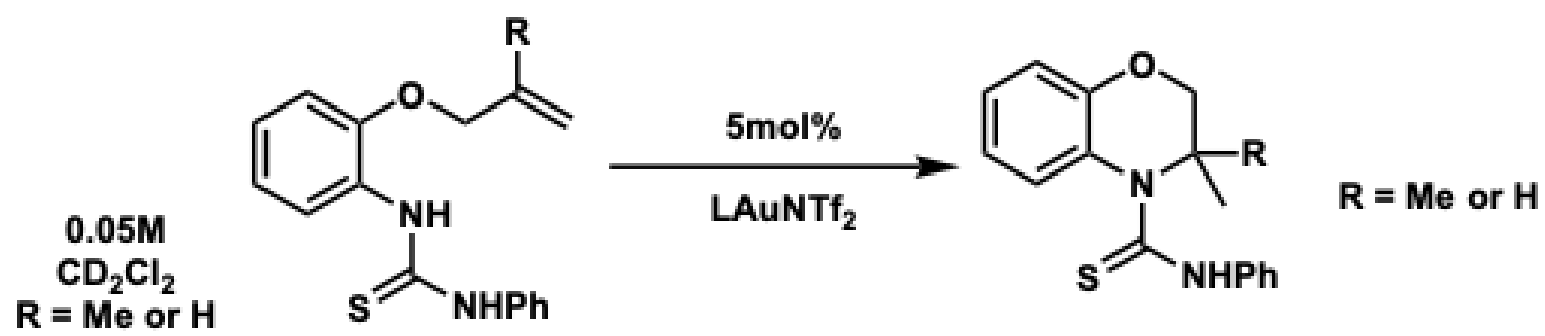

Entry	experiment code	substrate	ligand	catalyst amount	catalyst loading	outcome
1	JM 5027	R = CH_3	JackiePhos	0.00175 mmol	5 mol %	No Reaction
2	JM 5029	R = CH_3	JPhos	0.00175 mmol	5 mol %	No Reaction
3	JM 5031	R = H	JackiePhos	0.00175 mmol	5 mol %	No Reaction
4	JM 5033	R = H	JPhos	0.00175 mmol	5 mol %	No Reaction

Gold coordination to phenol thiourea.

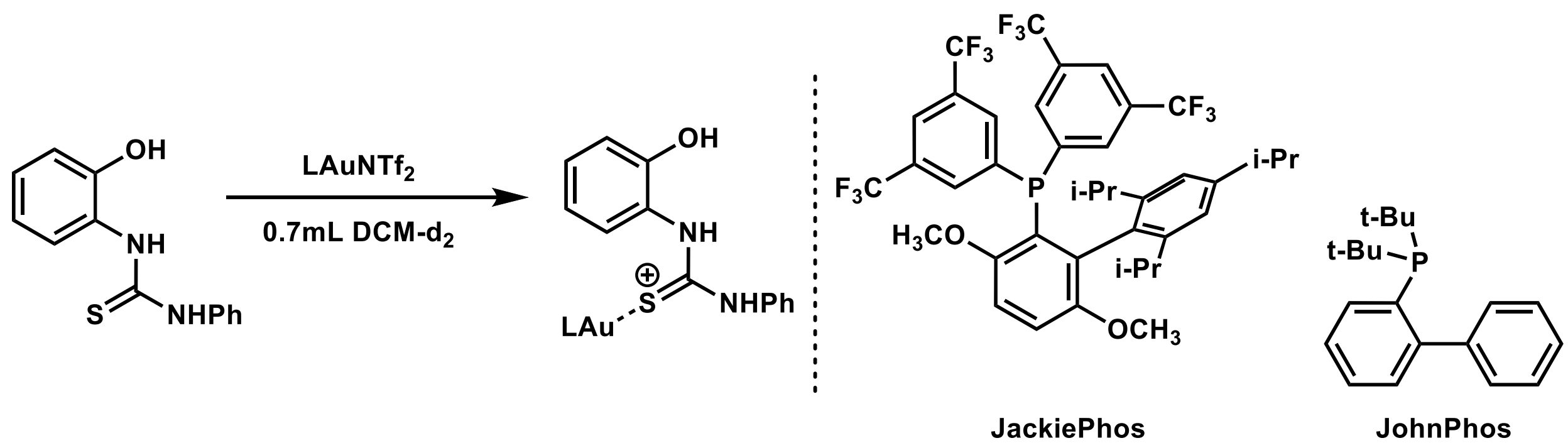

The phenol thiourea, (0.0041 mmol, 1 equiv) was weighed into a scintillation vial. The appropriate catalyst (0.0041 mmol, 1 equiv) was weighed into the same vial and dissolved in DCM-d_2 and transferred into an NMR tube.

Table S4. ^{31}P NMR chemical shifts for solutions of $LAuNTf_2$ coordinated to 1-(2-hydroxyphenyl)-3-phenylthiourea in DCM-d_2.

Entry	experiment code	ligand	^{31}P NMR
1	JM 5037	JackiePhos	23.5 ppm
2	JM 5037	JPhos	64.7 ppm

Impact of urea on acid reactivity

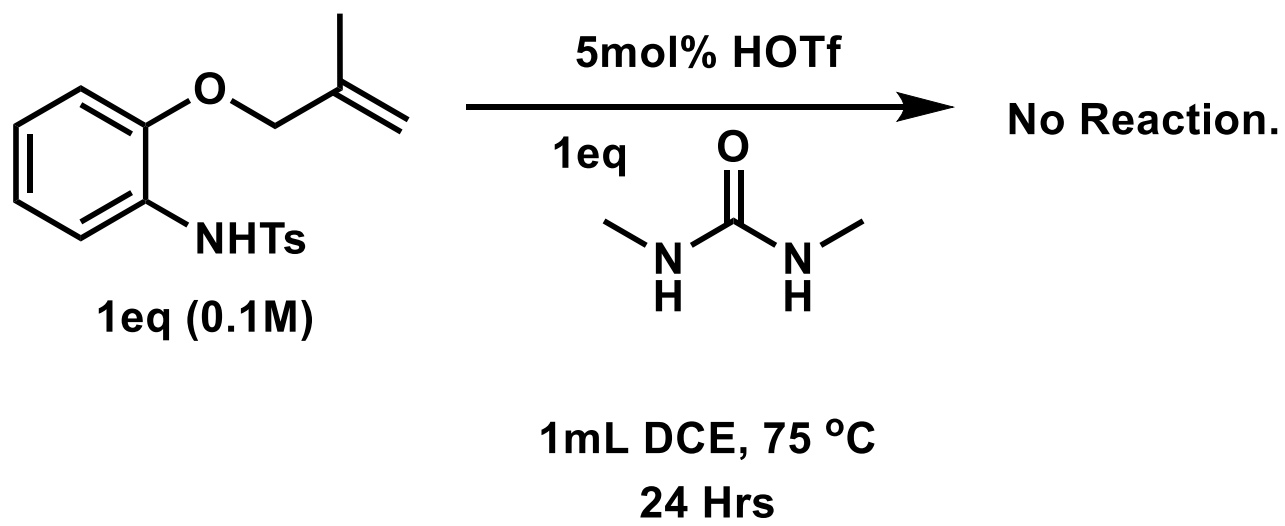

To a scintillation vial were added sulfonamide **4a** (0.1 mmol, 1 equiv) and dimethylurea (0.1 mmol, 1 equiv). The substrates were then dissolved in 1,2-dichloroethane (1 mL, 0.1 M) and HOTf (0.005 mmol, 5 mol %) was subsequently added. The vial was capped and parafilmed and allowed to heat at 75 °C for 24 hours. Upon completion, DCE was removed by vacuum, and an NMR was taken in $CDCl_3$. Only starting material was observed. (JM5111)

Reactivity of alkenyl amines with allylic tethers with JackiePhosAuNTf2 (cat. **A**)

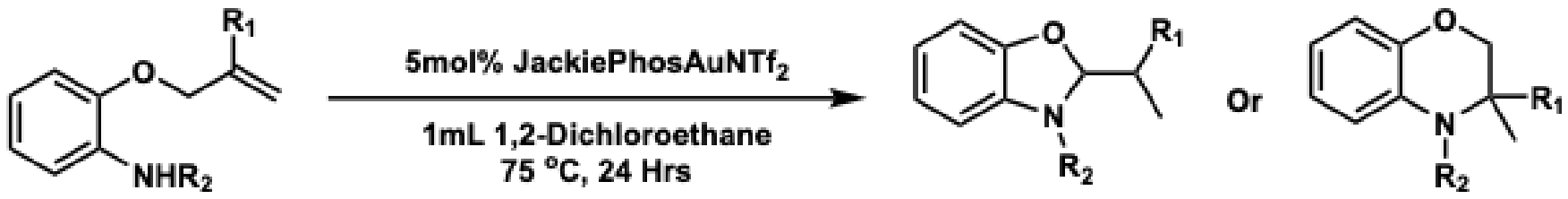

**Substrate**	**R1**	**R2**	**5-membered**	**6-membered**
Urea (H) (**7b**)	H	C(O)NH*t*-Bu	Not observed	Yes
Urea (Me)	CH_3	C(O)NH*t*-Bu	Not observed	No
Tosylamide (H)	H	Ts	No	Not observed
Tosylamide (Me) (**4a**)	CH_3	Ts	Yes	Not observed
Thiourea (H)	H	C(S)NHPh	Not observed	Not observed
Thiourea (Me)	CH_3	C(S)NHPh	Not observed	Not observed

Substrate (0.1 mmol, 1 equiv), catalyst (0.005 mmol, 5 mol %) DCE (1 mL, 0.1 M)

Additional control experiments for 1-allyl-1,3-diphenylurea (**1**)

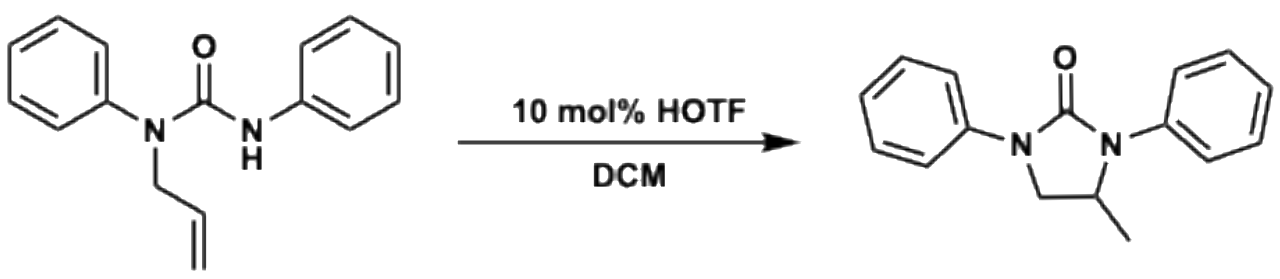

**Entry**	**experiment code**	**acid loading**	**outcome**
1	AYQ2035	10 mol %	No Rxn

Substrate (0.14 mmol, 1 equiv), Acid (0.014 mmol, 10 mol %) DCM (0.7 mL, 0.2 M)

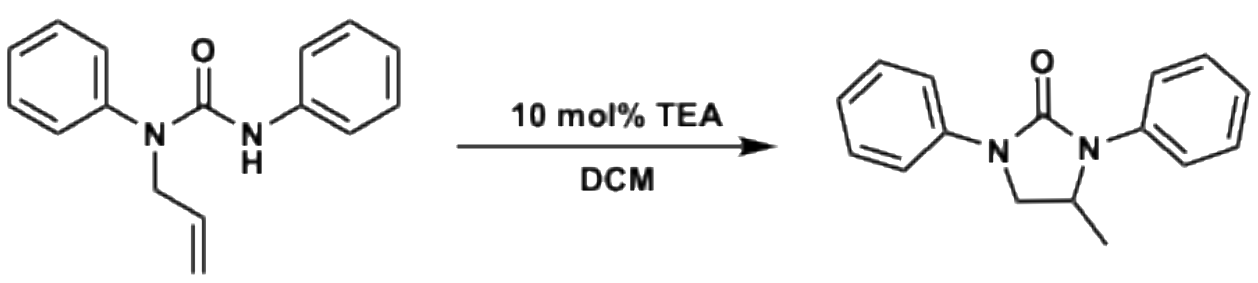

**Entry**	**experiment code**	**base loading**	**outcome**
1	AYQ2053	10 mol %	No Rxn

Substrate (0.14 mmol, 1 equiv), Base (0.014 mmol, 10 mol %) DCM (0.7 mL, 0.2 M)

Characterization of alkylgold from reactivity of 1-allyl-1,3-diphenylurea (**1**)

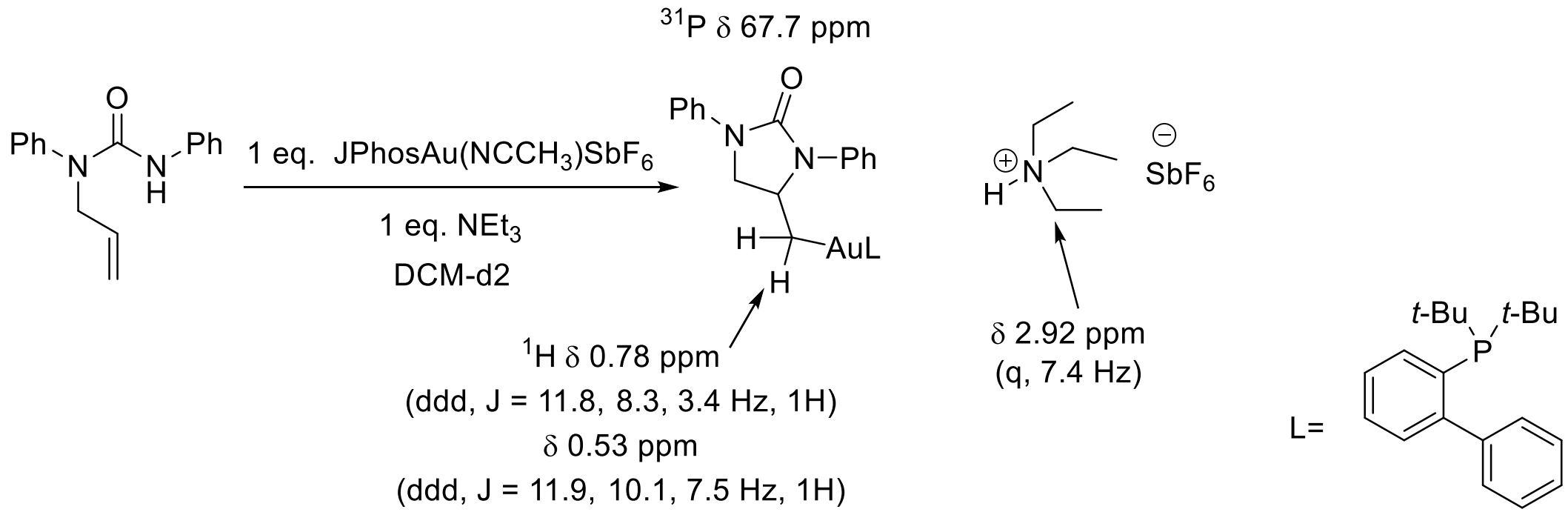

Selected Alkylgold Peaks: ^{1}H NMR (400 MHz, Methylene Chloride-d_2) δ 4.58 – 4.47 (m, 1H), 3.97 (t, J = 8.7 Hz, 1H), 3.41 (dd, J = 8.6, 7.6 Hz, 1H), 0.78 (ddd, J = 11.8, 8.3, 3.4 Hz, 1H), 0.53 (ddd, J = 11.9, 10.1, 7.5 Hz, 1H).

2.92 (q, J = 7.4 Hz) $[HNEt_3]^+$ (Cosy CrossPeak with broad singlet at 7.8 ppm)

2.83 (qd, J_{HH}= 7.2 Hz, J_{HP}=2.4 Hz) $[LAuNEt_3]^+$

^{31}P: 67.7 ppm

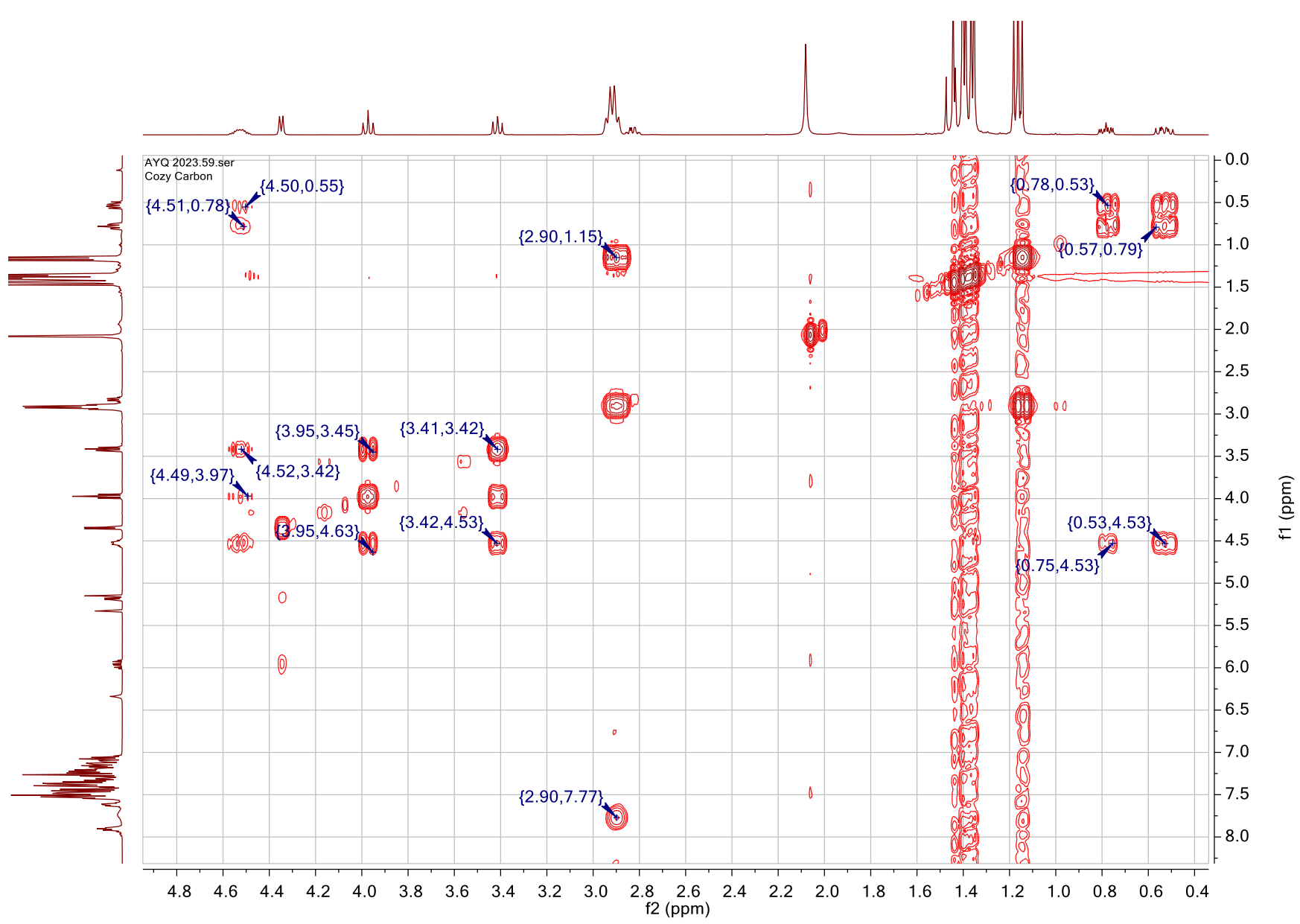

Figure S21. COSY spectrum for alkylgold with JPhos (cat. **B**).

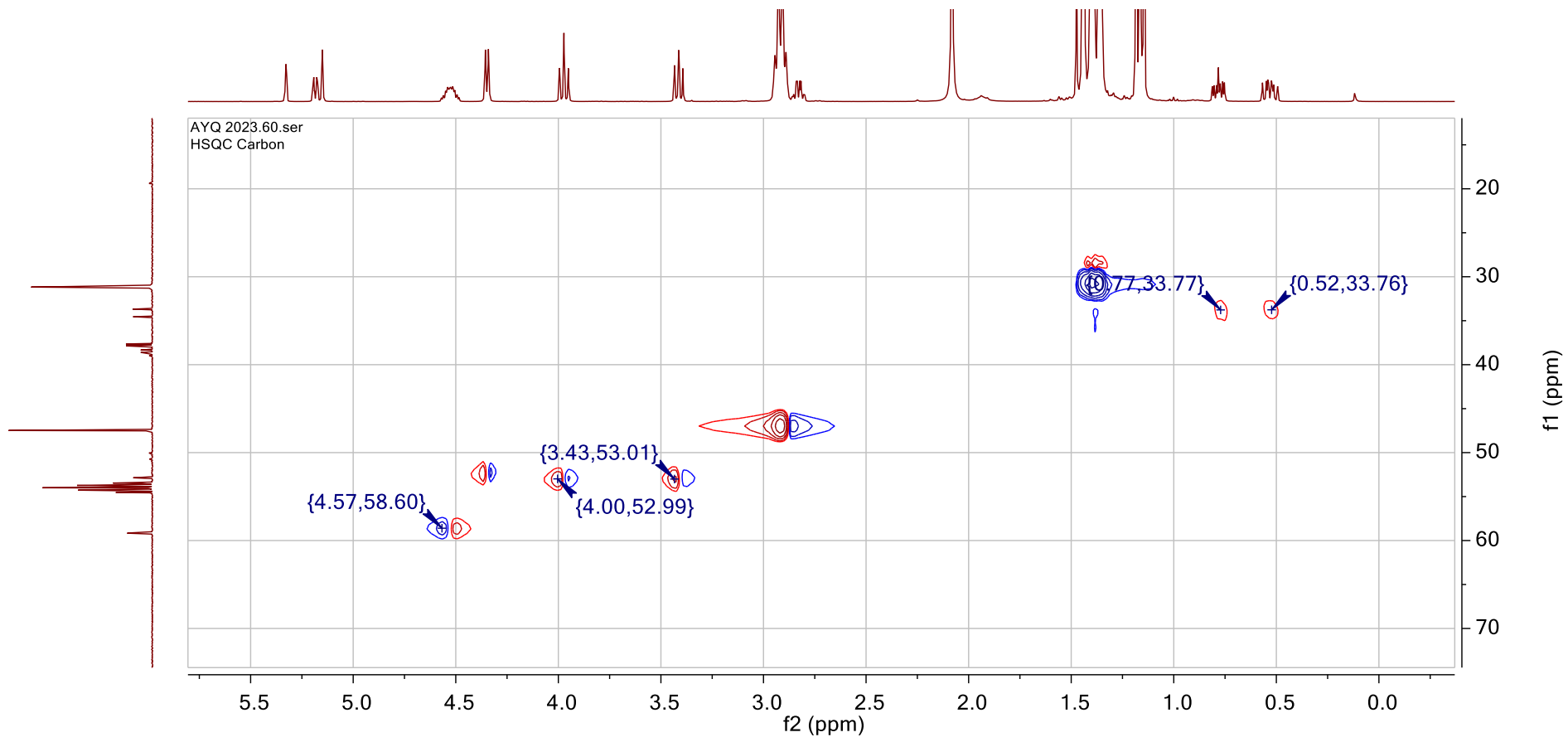

Figure S22. HSQC spectrum for alkylgold with JPhos (cat. **B**).

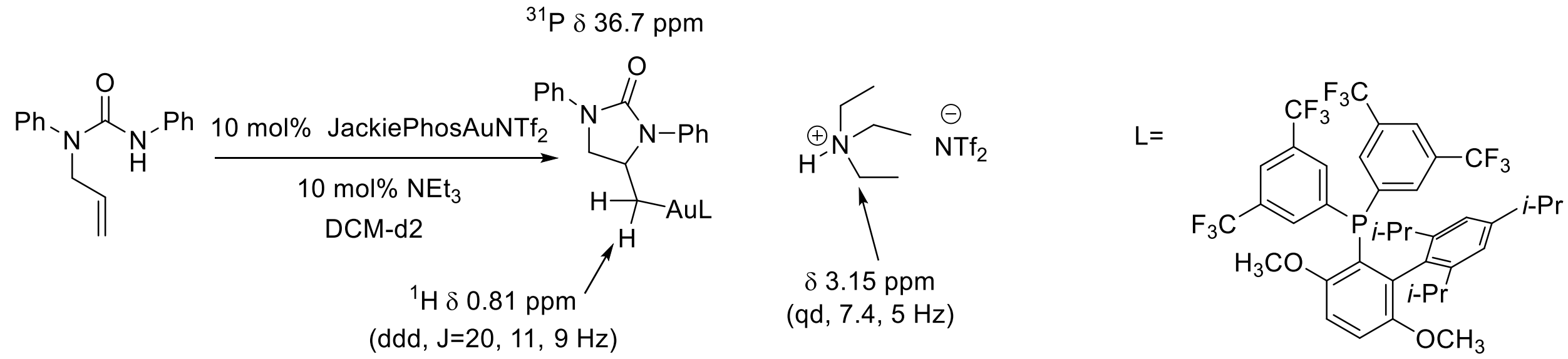

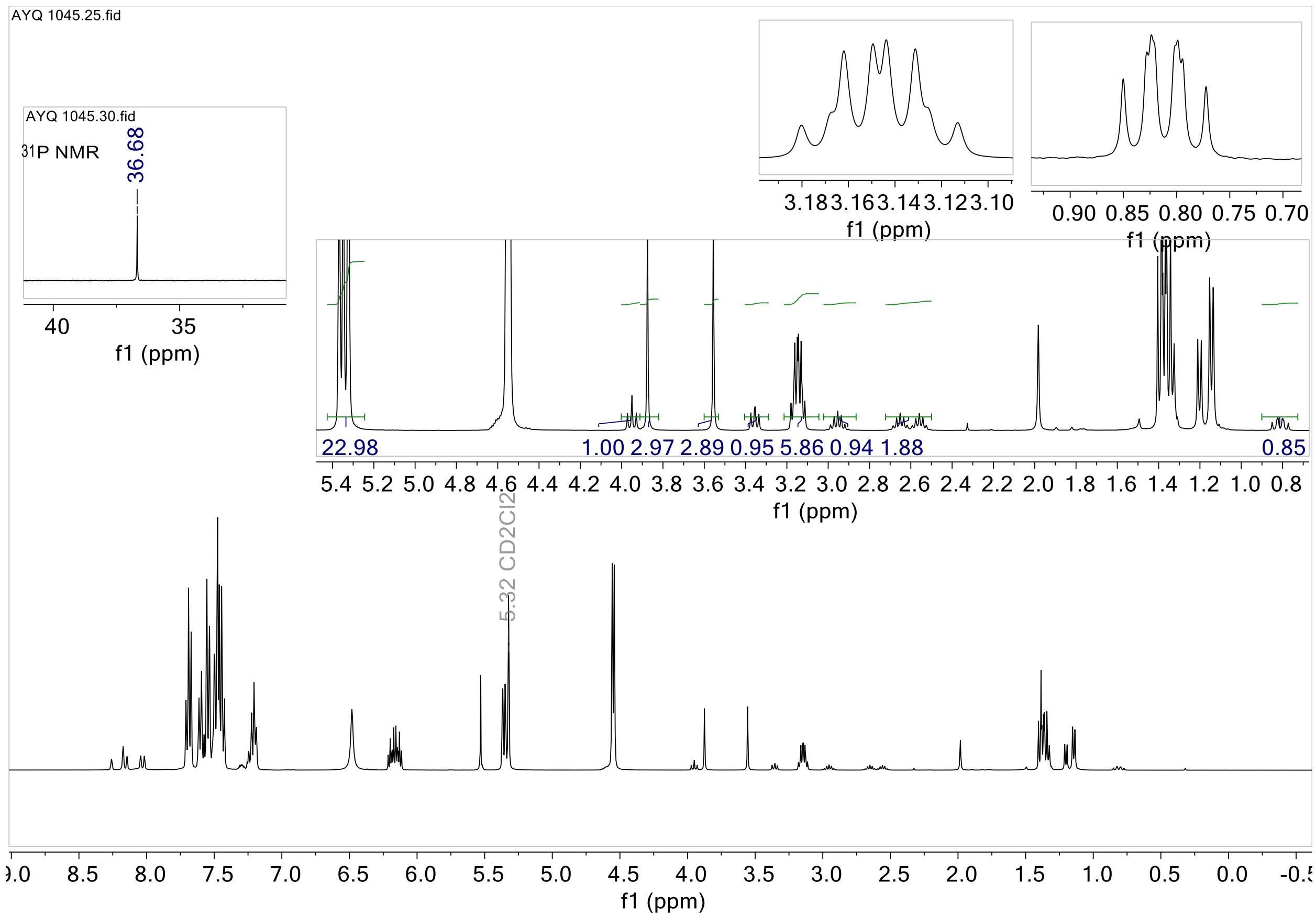

Figure S23. ^{1}H and ^{31}P NMR spectra showing alkylgold formation from reaction of urea with $LAuNTf_2/NEt_3$ at = 24 h.

Identification of alkylgold from reactivity of 1-(*tert*-butyl)-3-(pent-4-en-1-yl)urea (**12**)

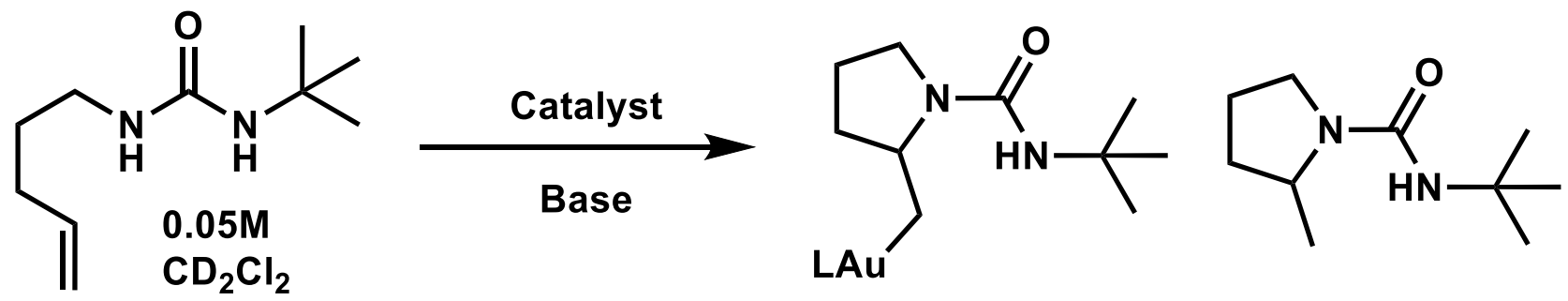

Entry	experiment code	catalyst	catalyst loading	base	base loading	alkylgold	$10^5 \times k_{obs}$ [s^{-1}]
1	JM 6019	$JPhosAu(NCCH_3)SbF_6$ (cat. **B**)	20 mol %	NEt_3	20 mol %	No	0.008
2	JM 6029	$JackiePhosAuNTf_2$ (cat. **A**)	20 mol %	NEt_3	20 mol %	Yes	0.06

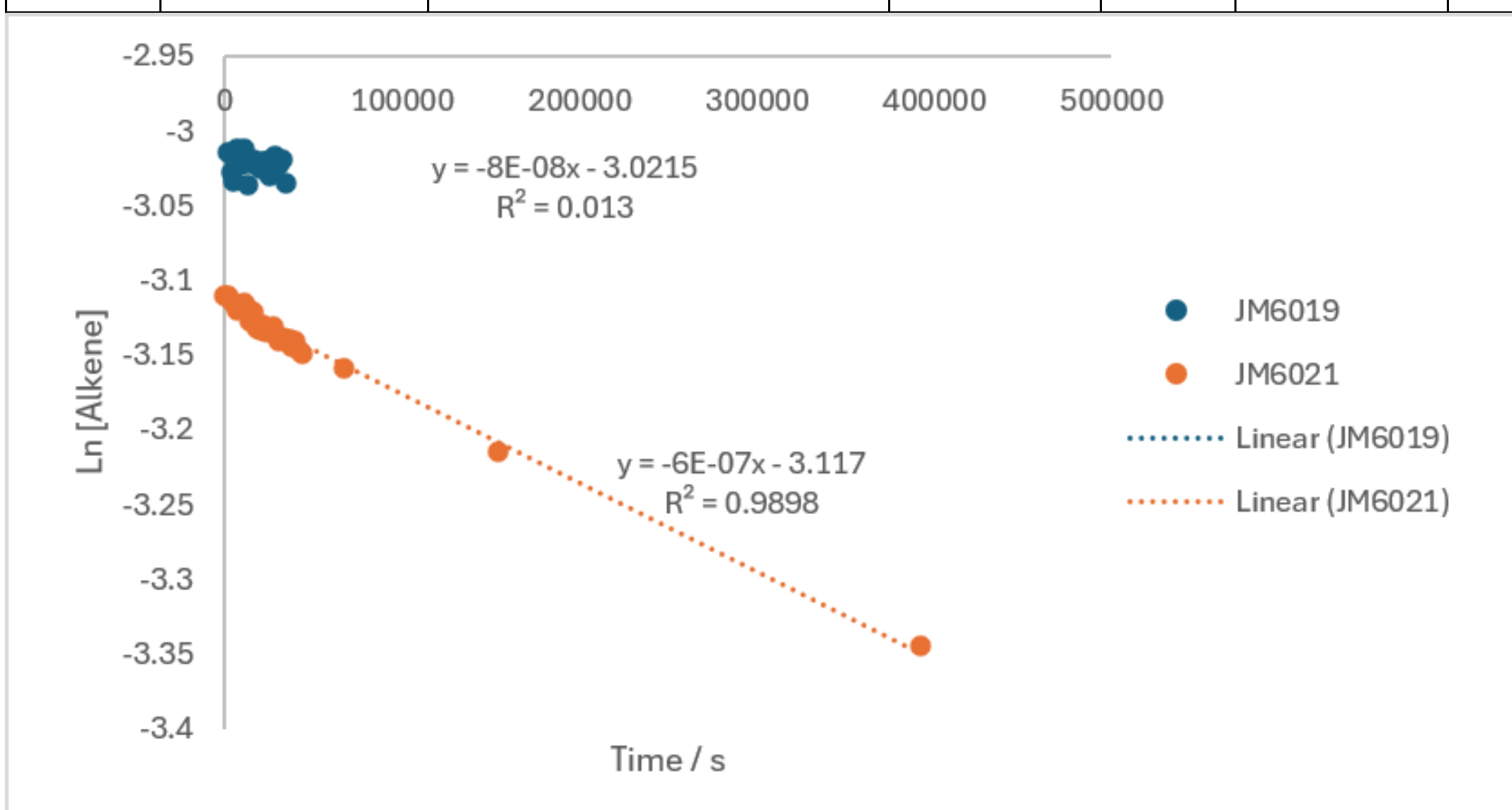

Figure S24. First order plots for disappearance of alkene with with cat. **A** and **B** and triethylamine.

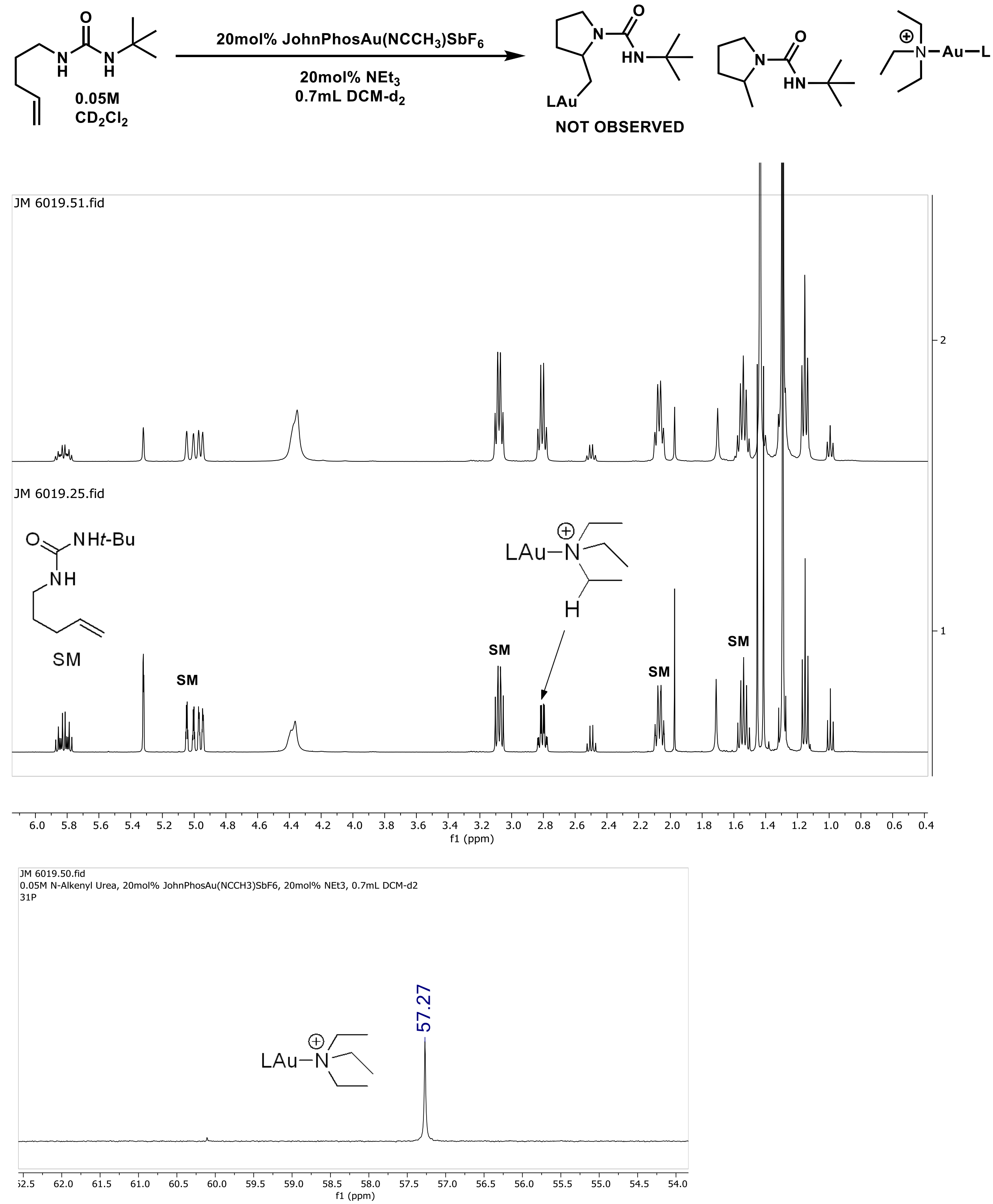

Figure S25. ^{1}H and ^{31}P NMR spectra showing absence of alkylgold formation from reaction of urea with [JPhosAuNET$_3$]SbF$_6$.

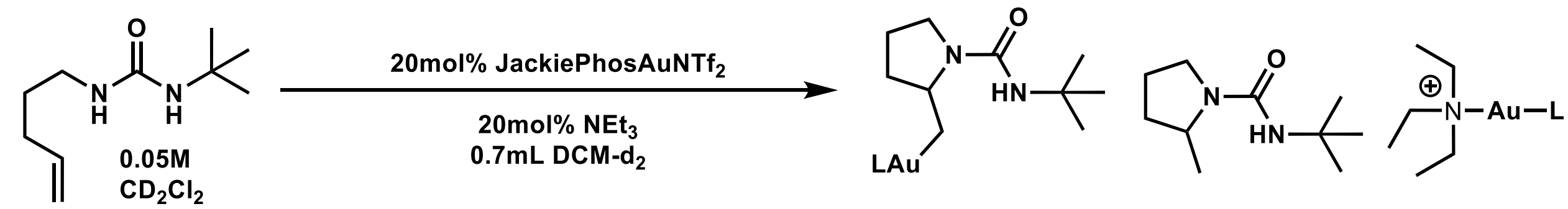

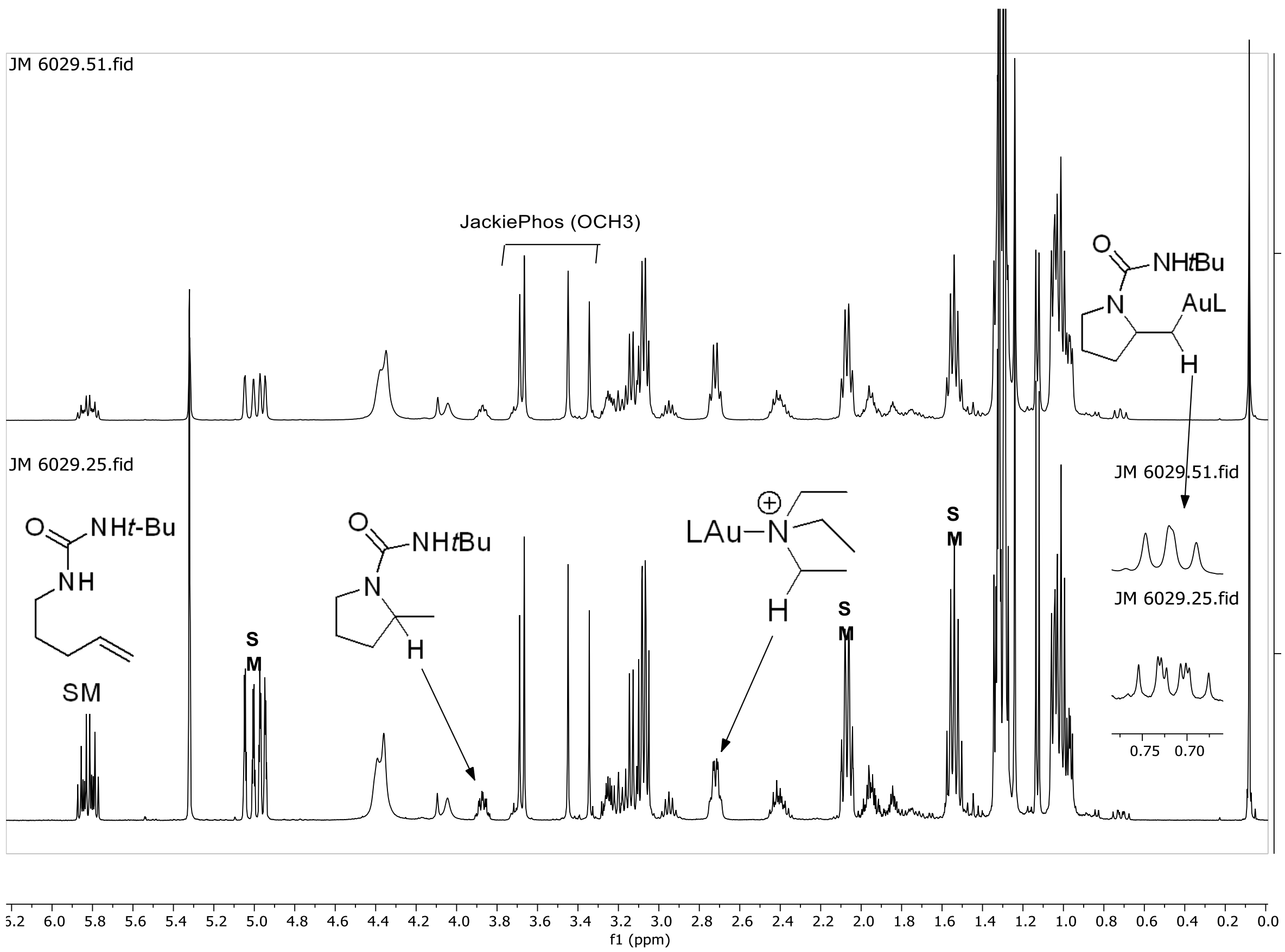

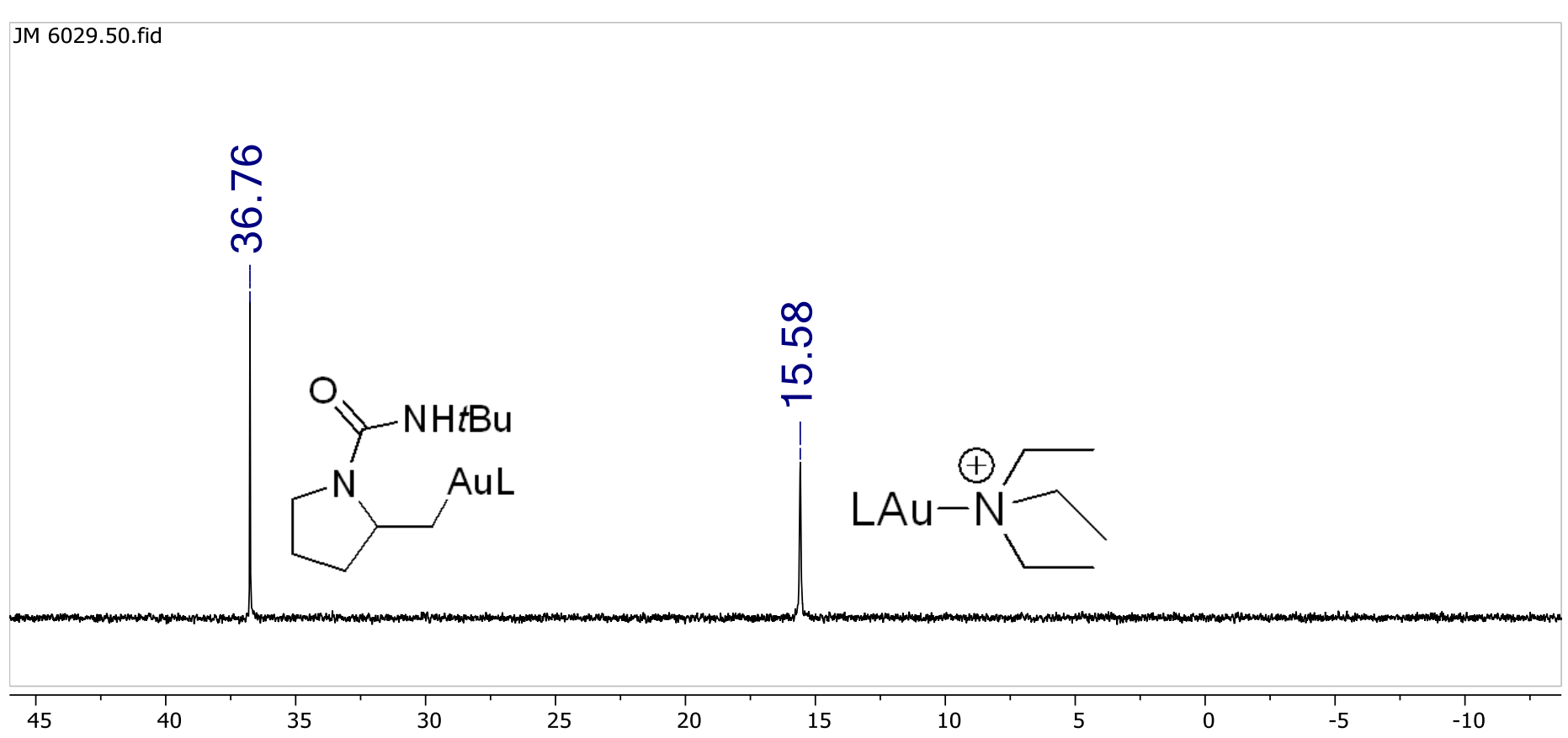

Figure S26. ^{1}H and ^{31}P NMR spectra showing alkylgold formation from reaction of **12** with cat. **A**/NET3.

Gold interactions with carbamate, thiocarbamate, thiourea

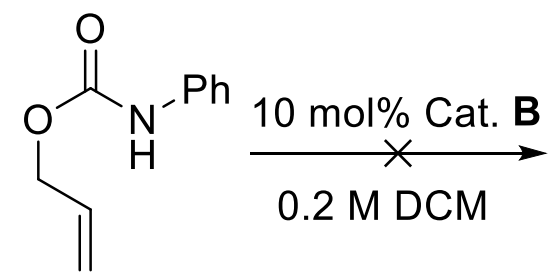

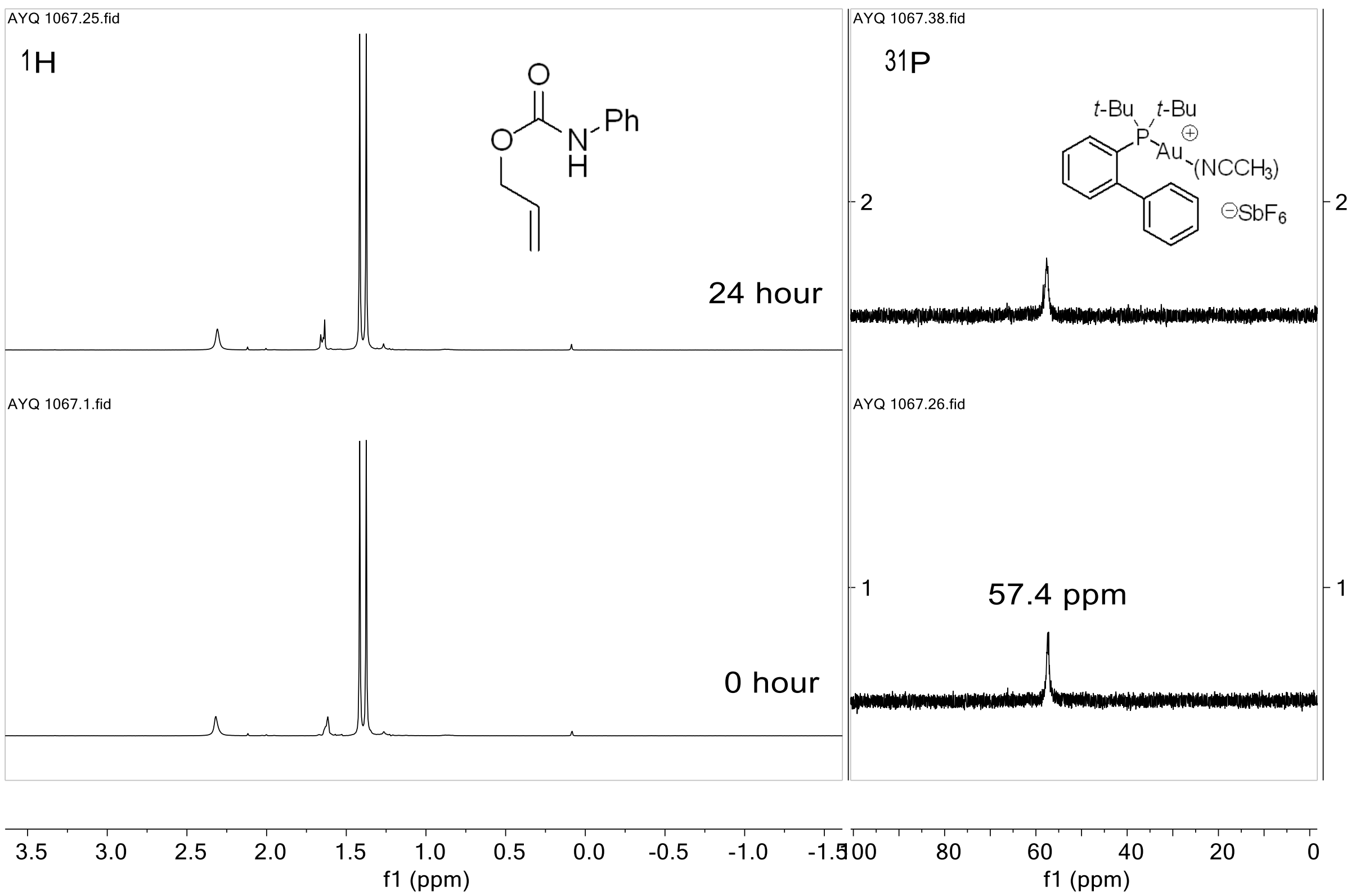

Figure S27. ^{1}H NMR and ^{31}P NMR spectra of **15** with cat. **B** at *t* = 0 and *t* = 24 hours. Only starting material and catalyst are seen.

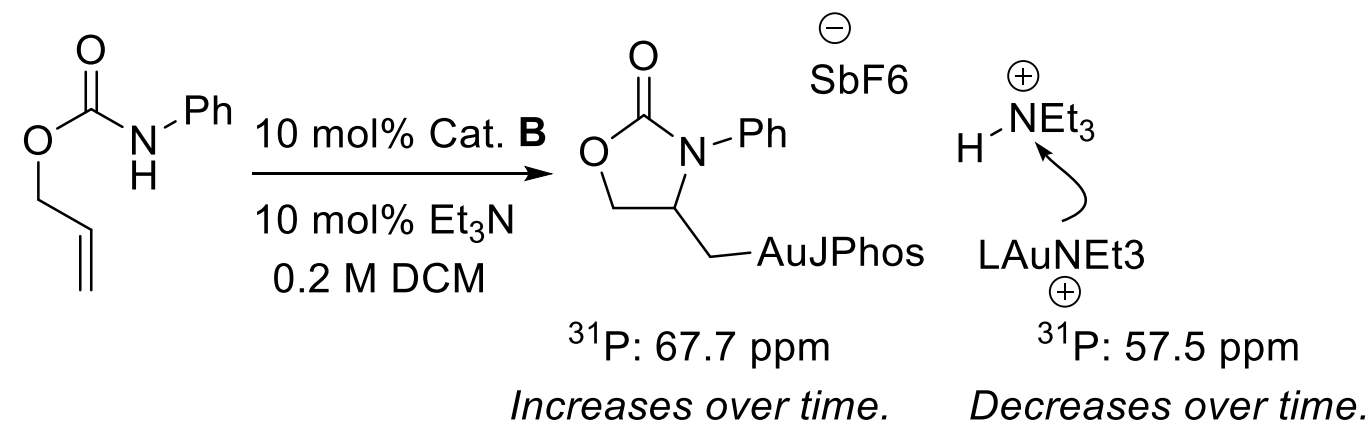

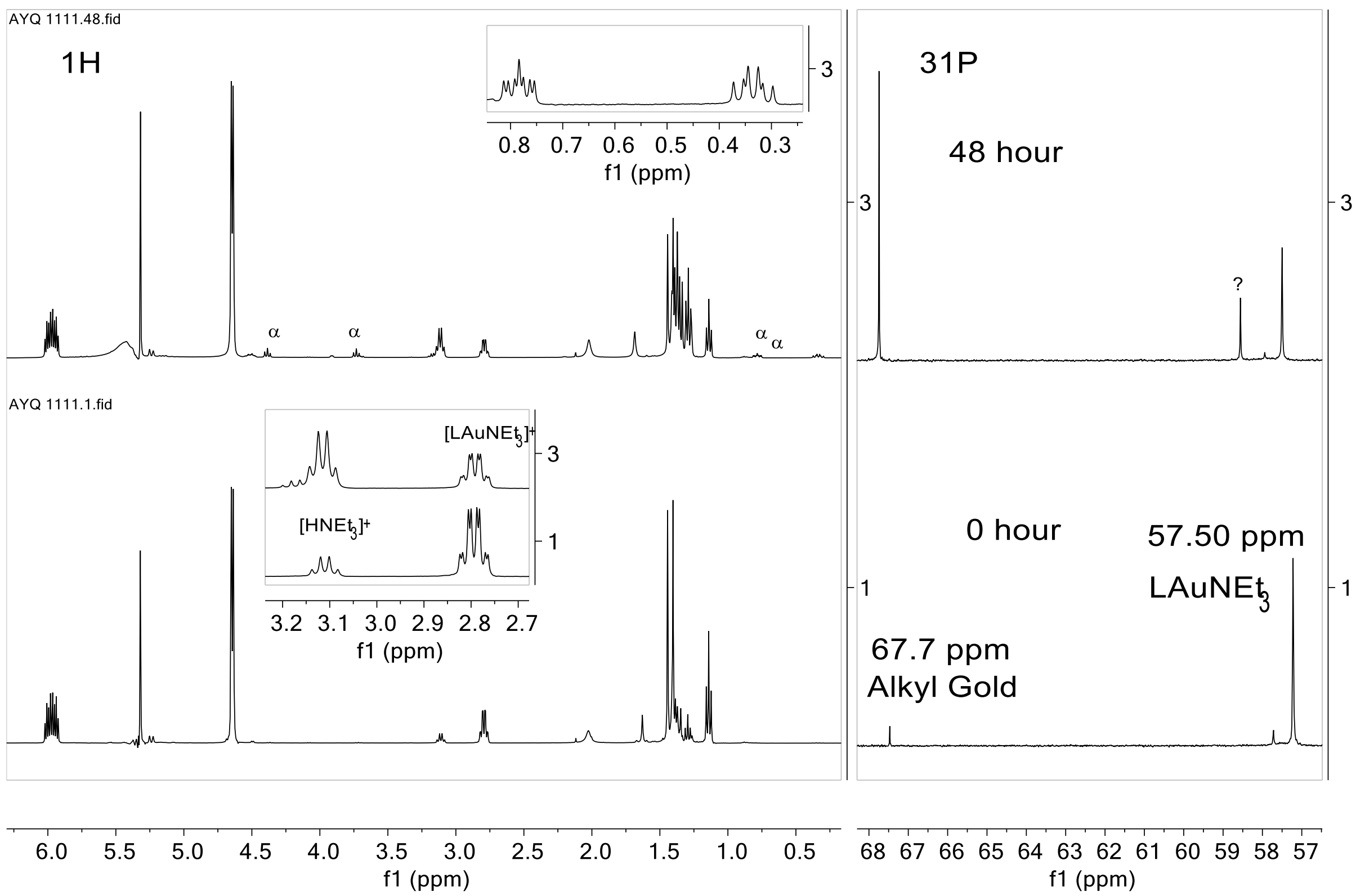

Figure S28. ^{1}H NMR and ^{31}P NMR spectra of 15 with cat. **B** and Et_3N at *t* = 0 and *t* = 48 hours. $[JPhosAuNEt_3]SbF_6$ is observed initially as indicated by the δ 2.8 ppm quartet of doublets in the ^{1}H NMR spectrum and the dominant signal at δ 57.5 ppm in the ^{31}P NMR. As gold bound amine diminishes $HNEt_3$ increases alongside alkyl gold.

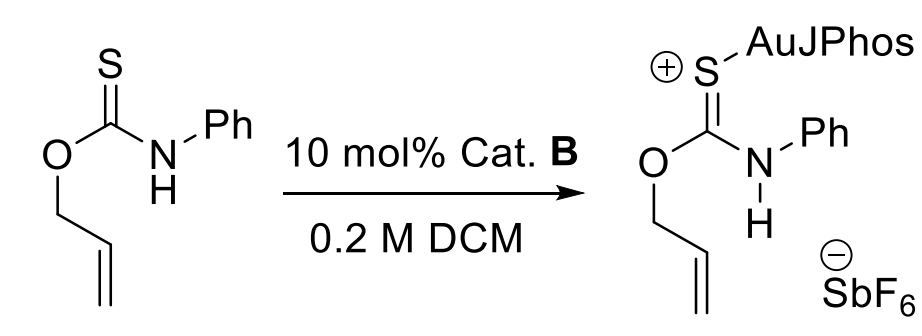

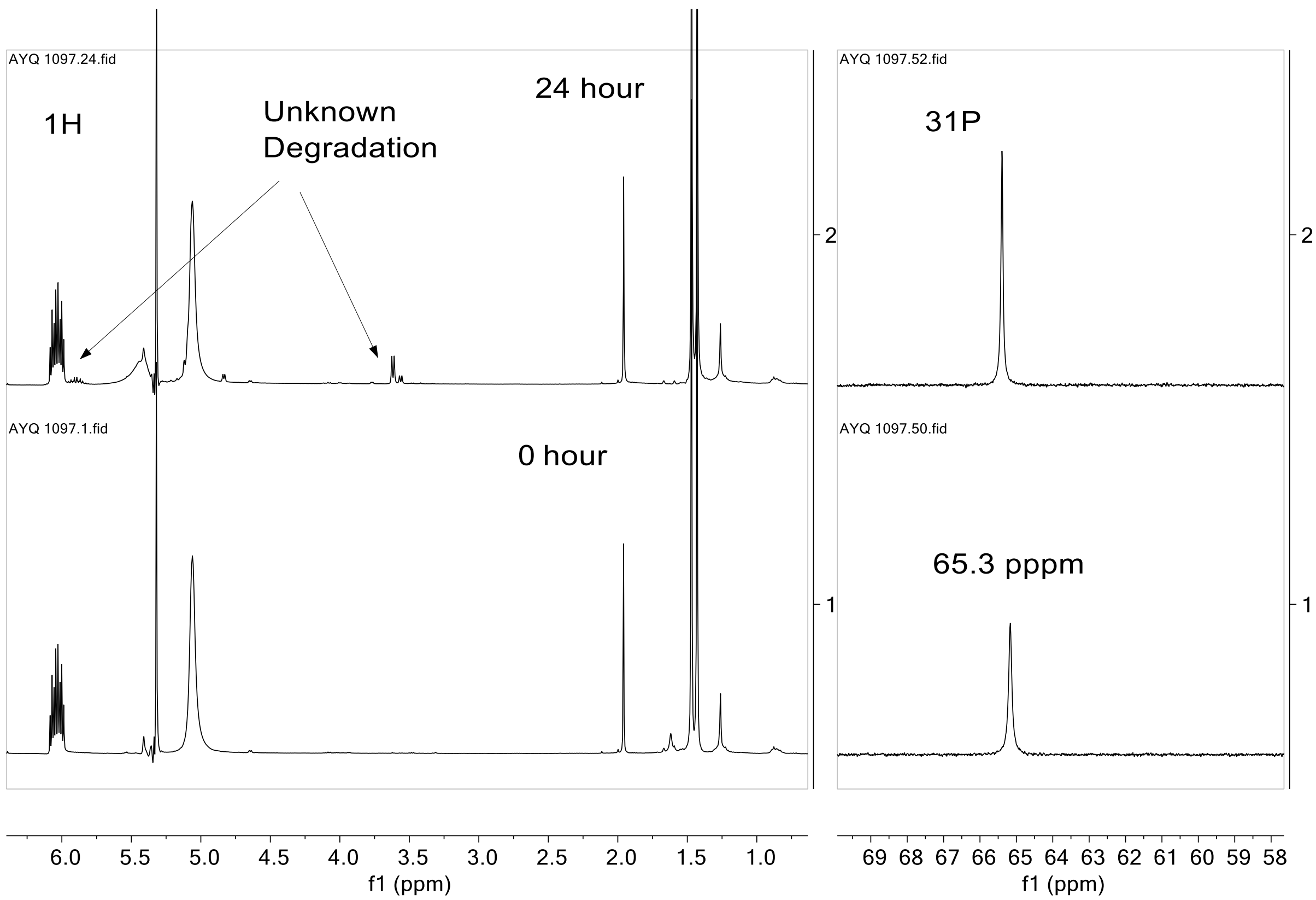

Figure S29. ^{1}H NMR and ^{31}P NMR spectra of **16** with cat. **B** at *t* = 0 and *t* = 24 hours. Only starting material and carbamate bound gold are seen with some unknown degradation.

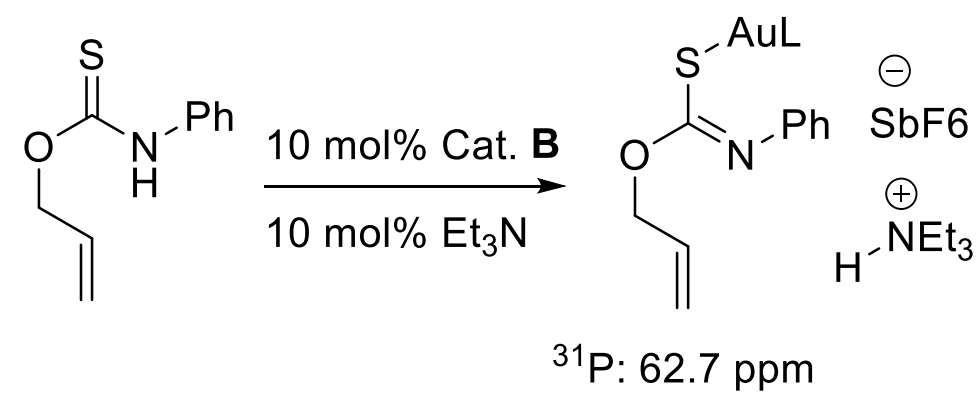

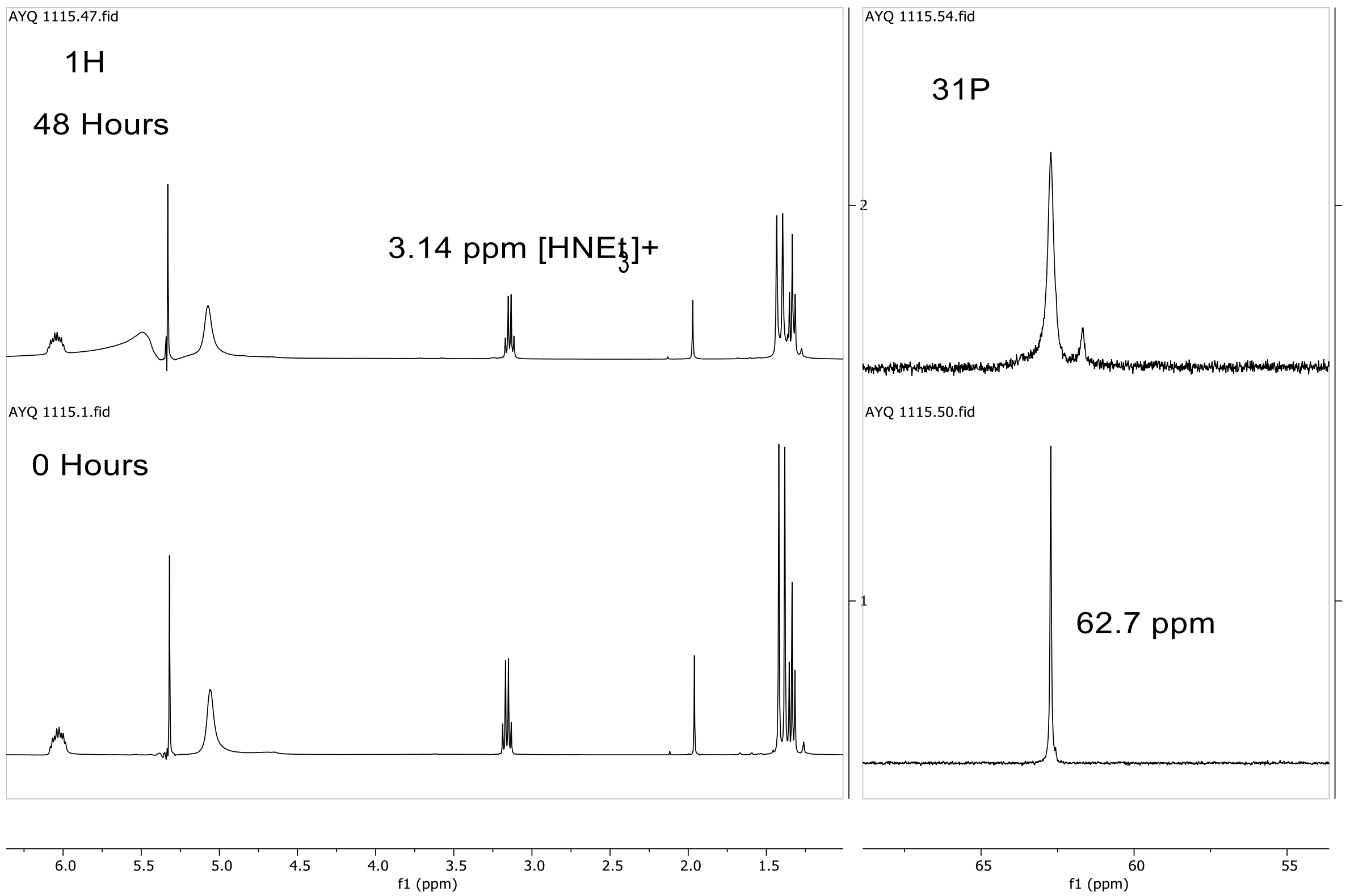

Figure S30. ^{1}H NMR and ^{31}P NMR spectra of **16** with cat. **B** and triethylamine at t = 0 and t = 48 hours.

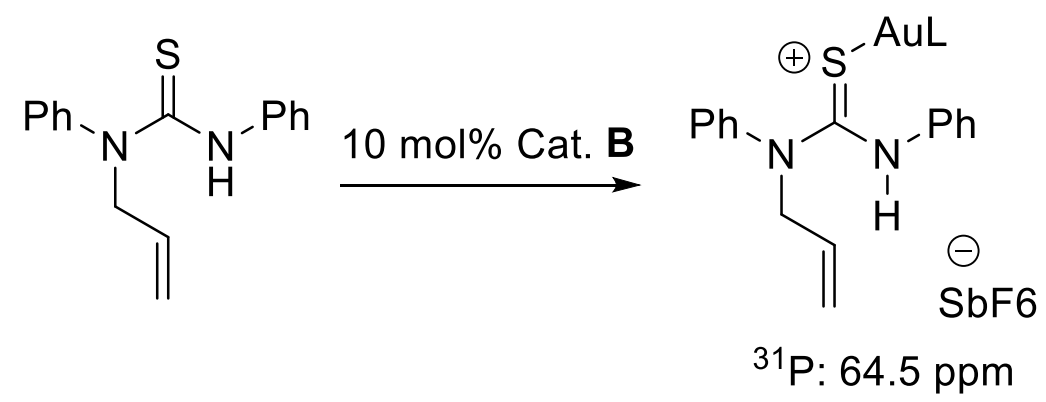

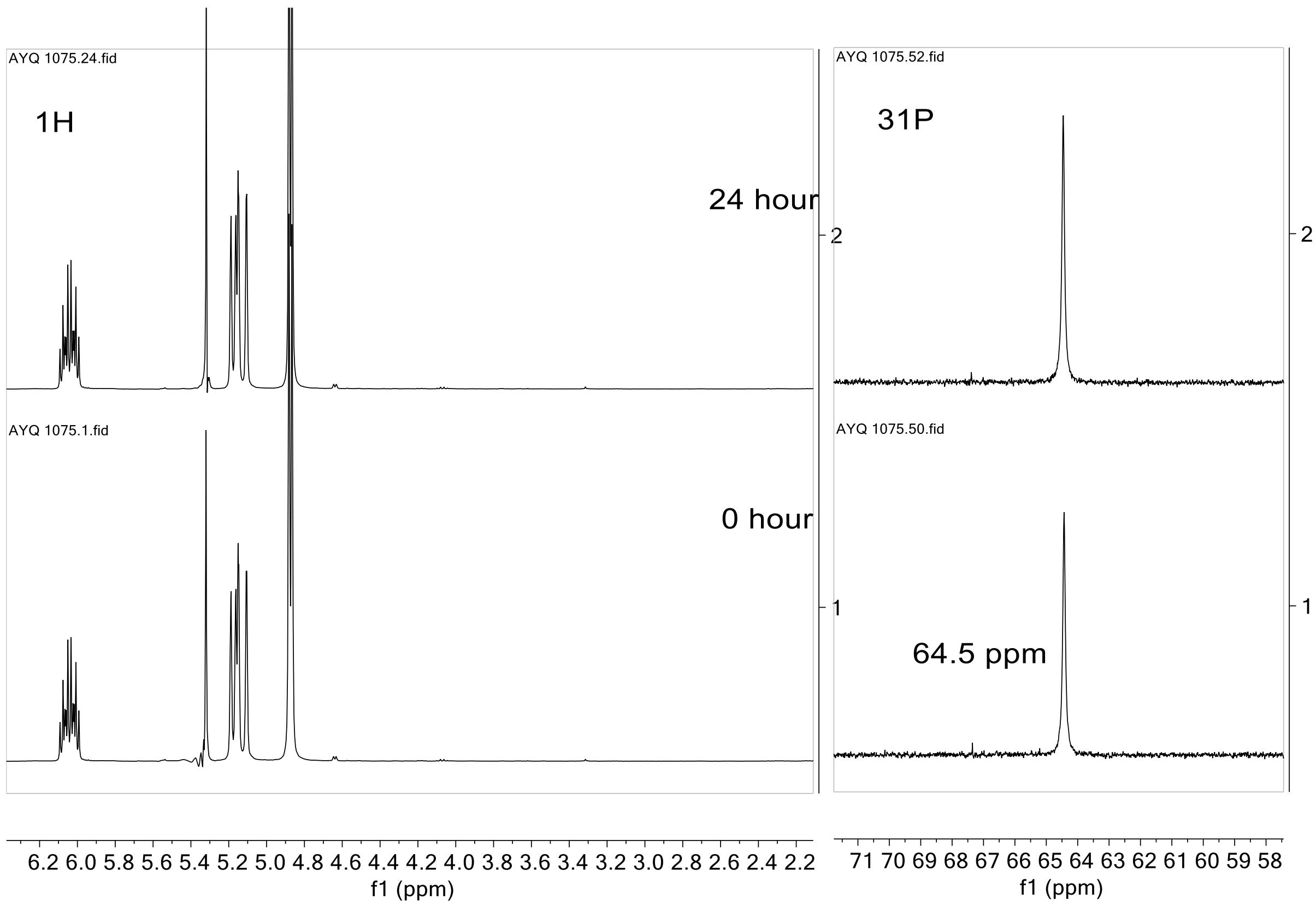

Figure S31. ^{1}H NMR and ^{31}P NMR spectra of **17** with cat. **B** at t = 0 and *t* = 24 hours. Only starting material and **17** bound gold are seen.

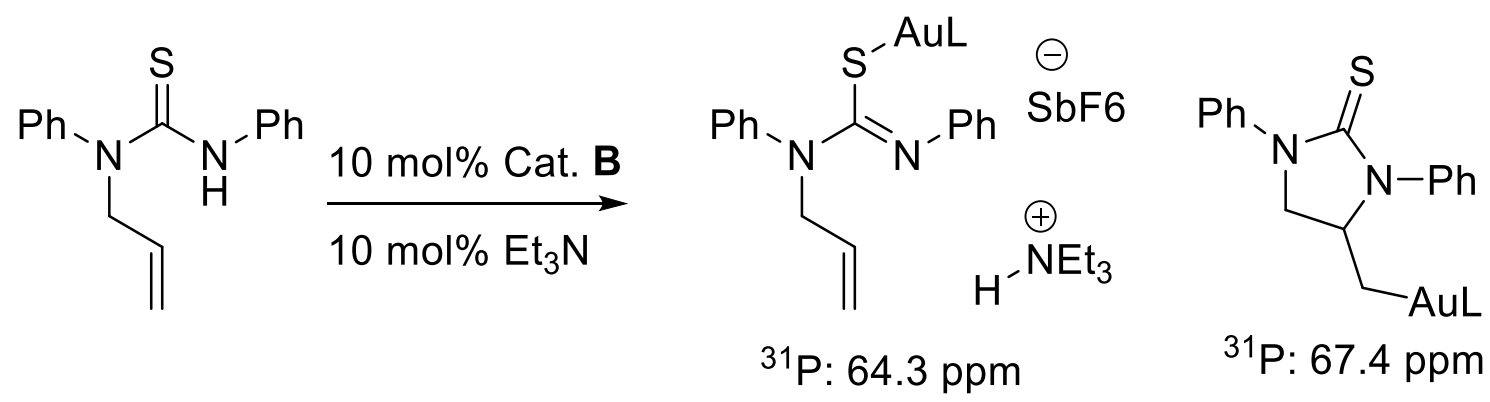

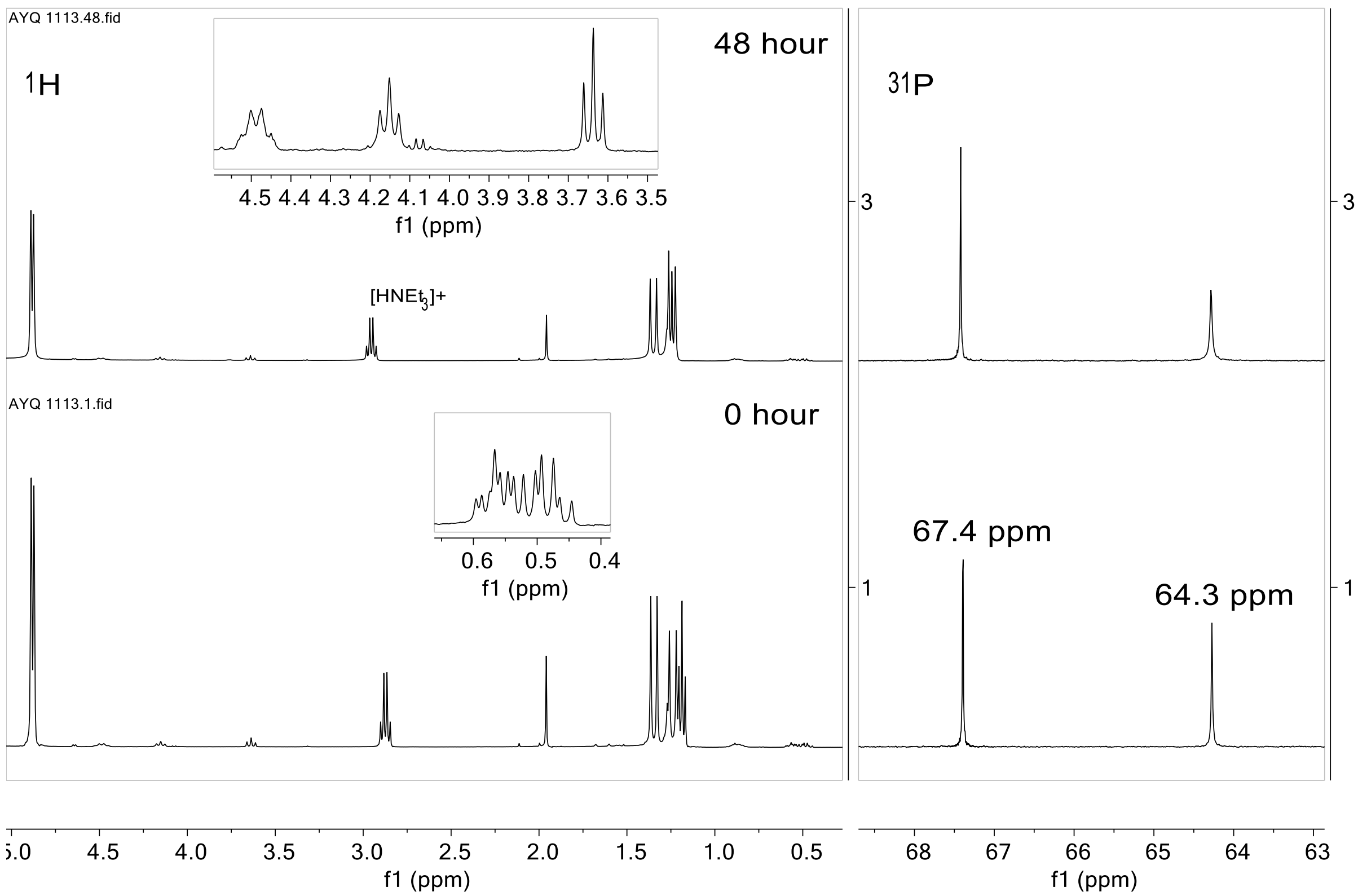

Figure S32. ^{1}H NMR and ^{31}P NMR spectra of **17** with cat. **B** and triethylamine at t = 0 and t = 48 hours. Alkylgold is seen clearly in the ^{1}H NMR while ^{31}P NMR indicates presence of **17** bound gold.

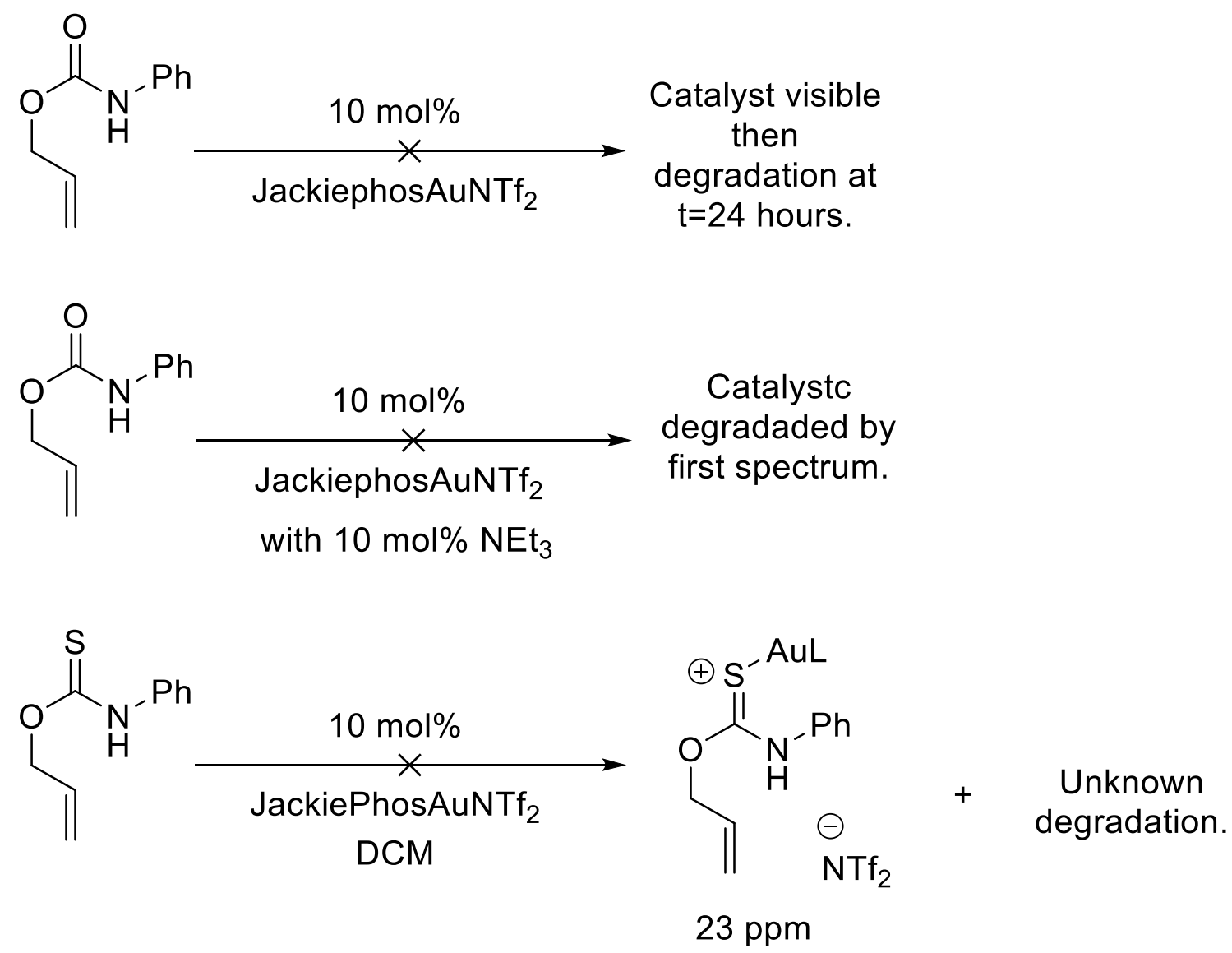

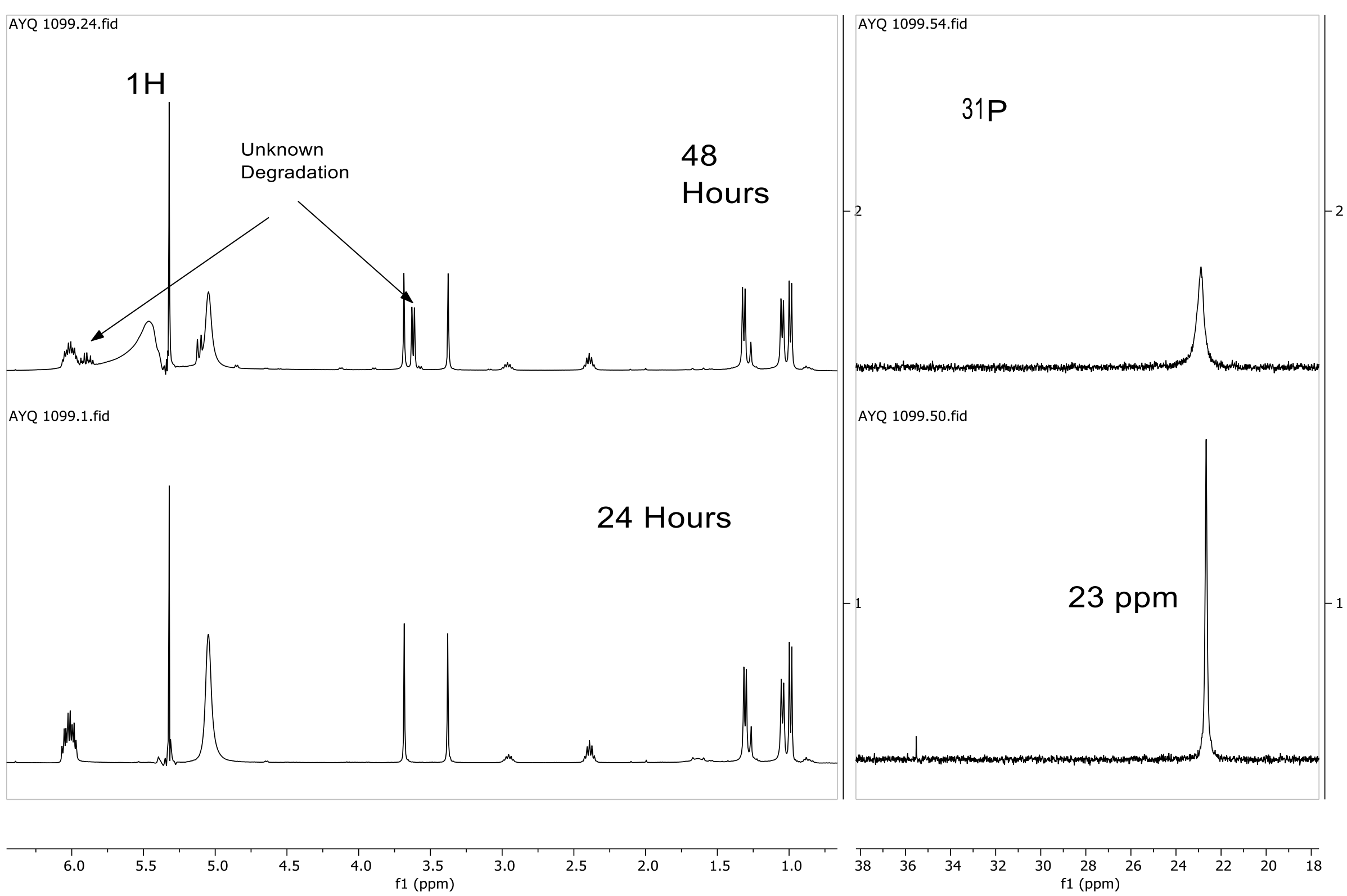

Figure S33. ^{1}H NMR and ^{31}P NMR spectra of thiocarbamate with JackiePhosAuNTf$_2$ (cat. **A**) at *t* = 0 and *t* = 48 hours showing gold bound thiocarbamate.

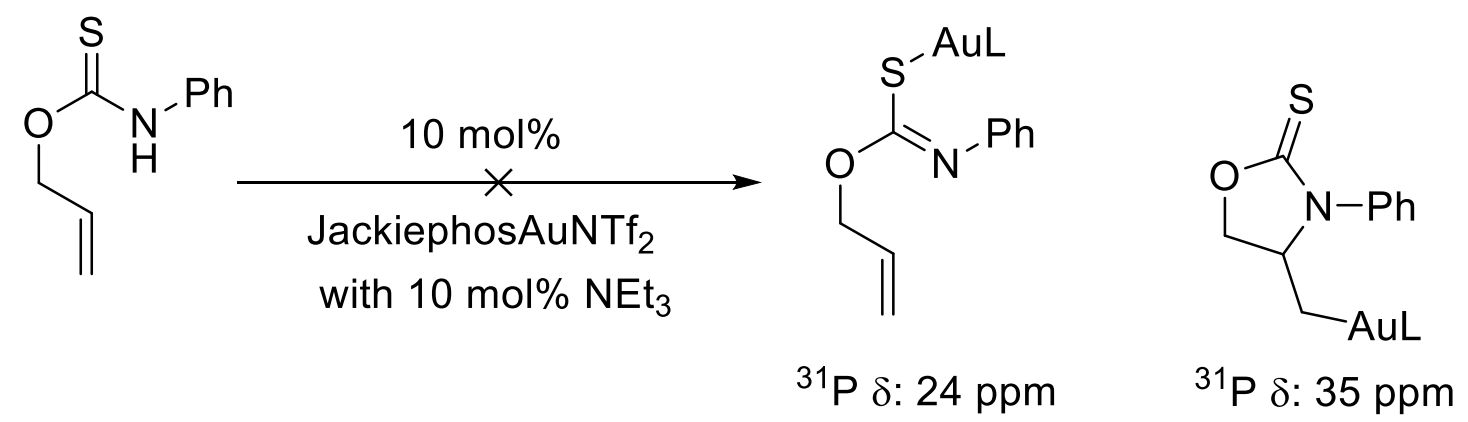

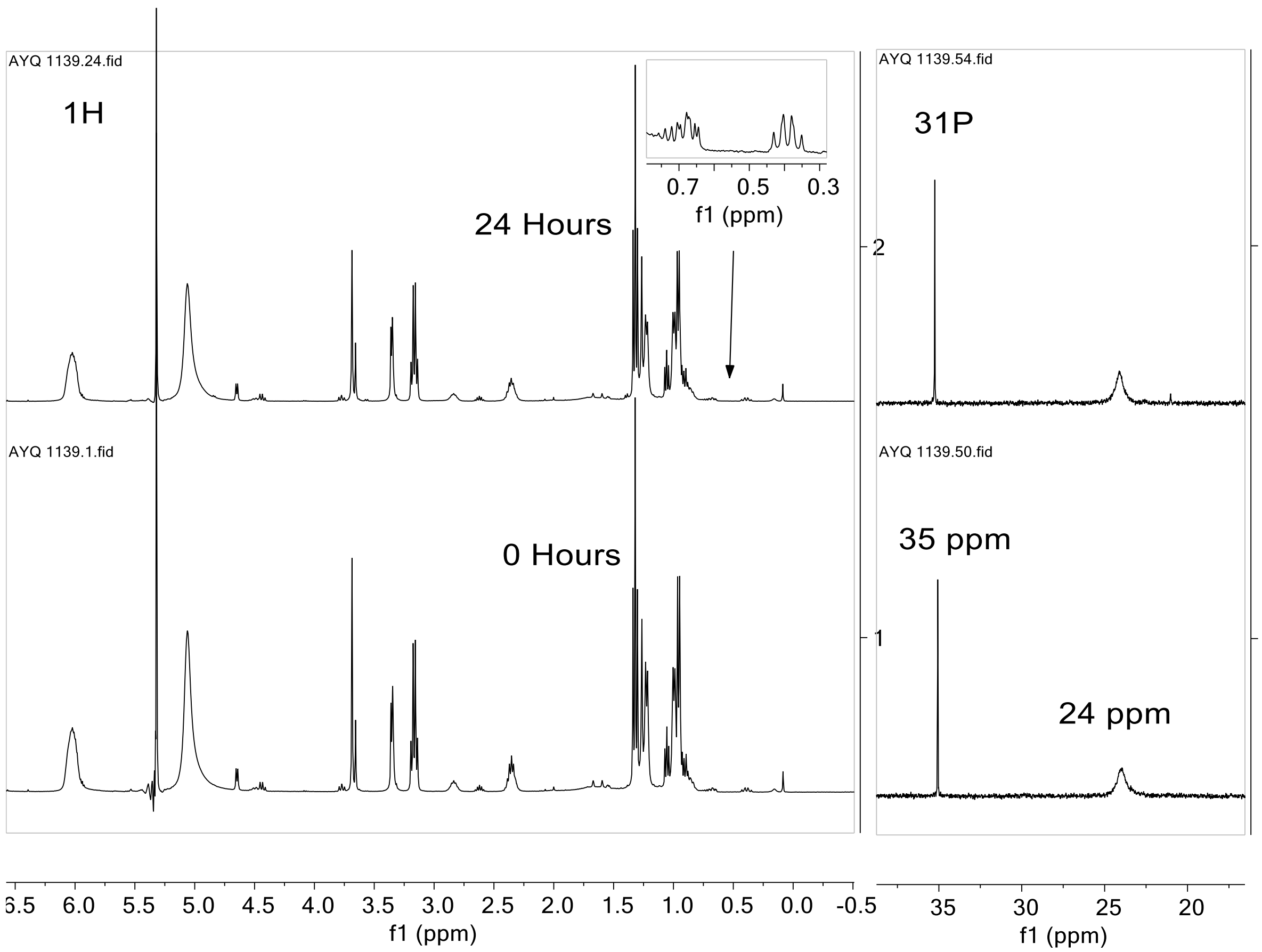

Figure S34. ^{1}H and ^{31}P NMR spectra of **16** with JackiePhosAuNTf$_2$ (cat. **A**) and triethylamine at *t* = 0 and *t* = 24 hours.

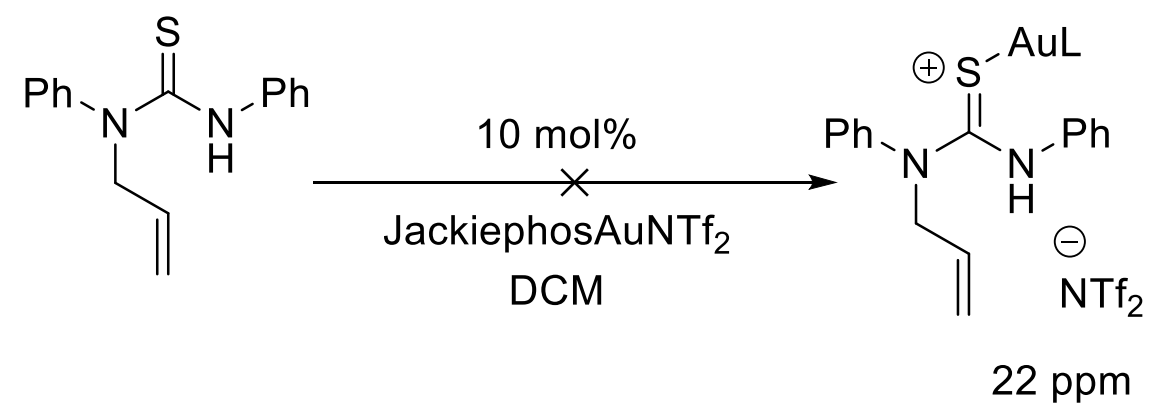

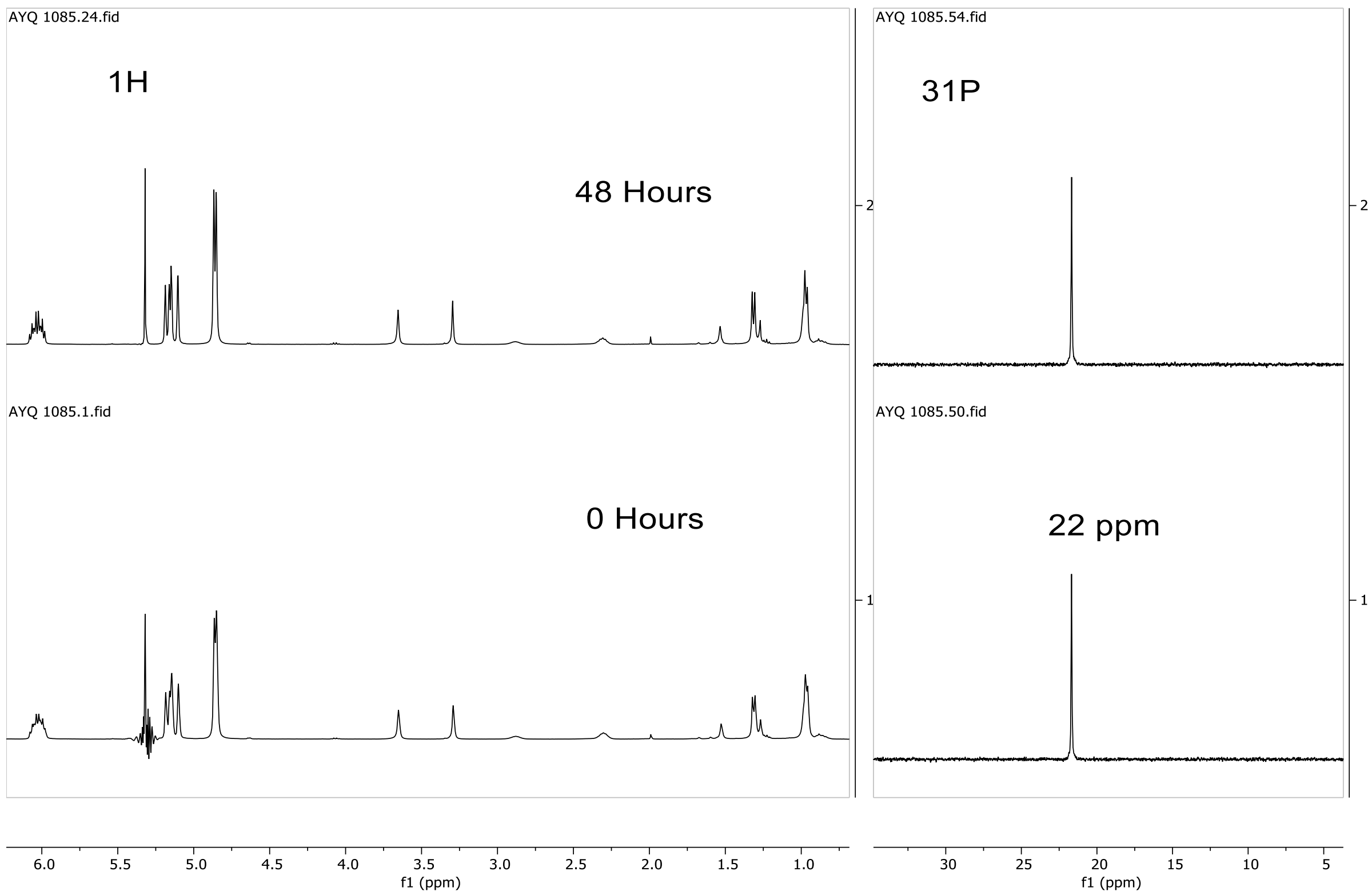

Figure S35. ^{1}H NMR and ^{31}P NMR spectra of **17** with JackiePhosAuNTf$_2$ (cat. **A**) at t = 0 and t = 48 hours.

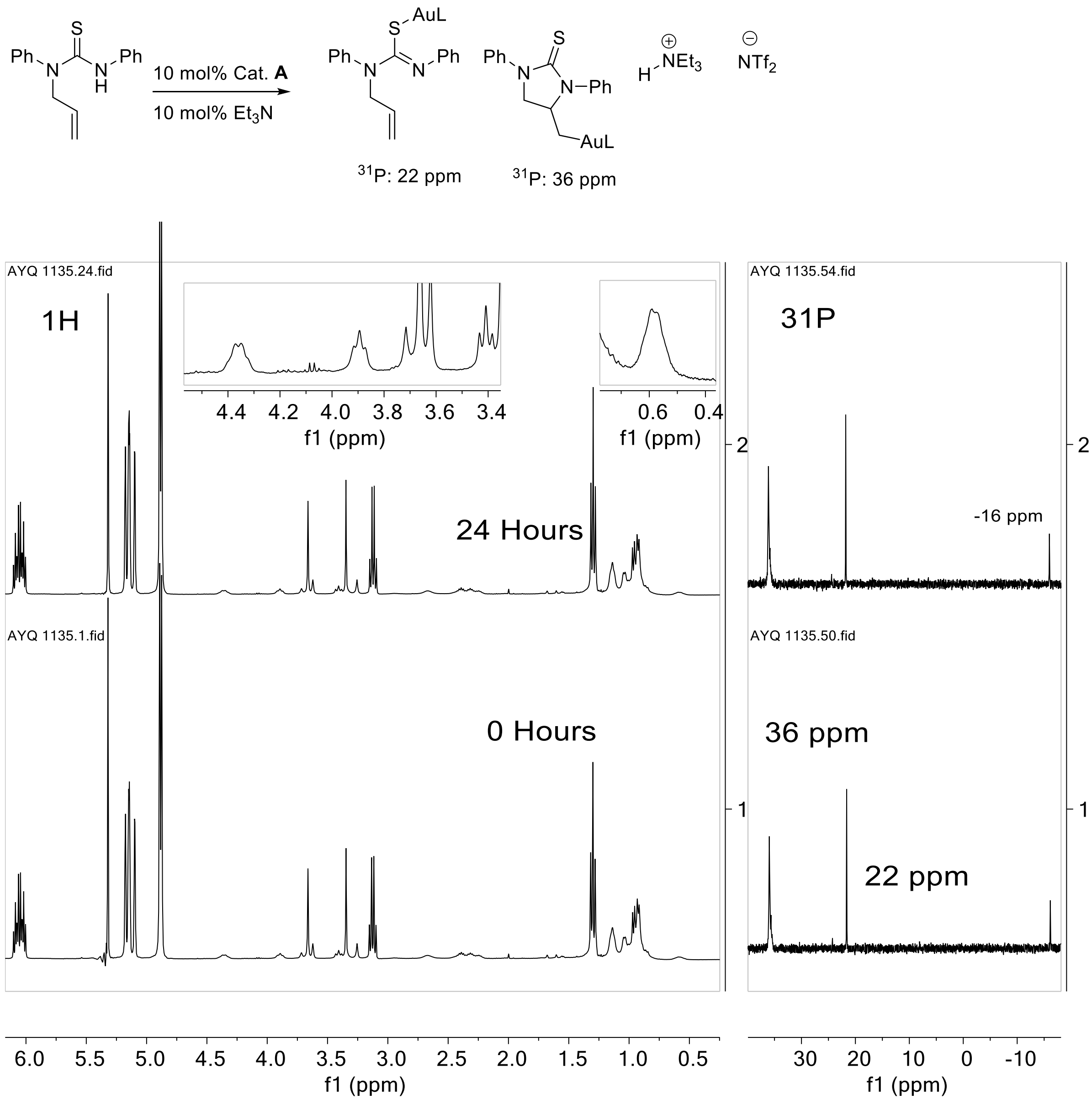

Figure S36. ^{1}H NMR and ^{31}P NMR spectra of **17** with JackiePhosAuNTf$_2$ (cat. **A**) and triethylamine at t = 0 and t = 24 hours.

NMR spectra of pure materials

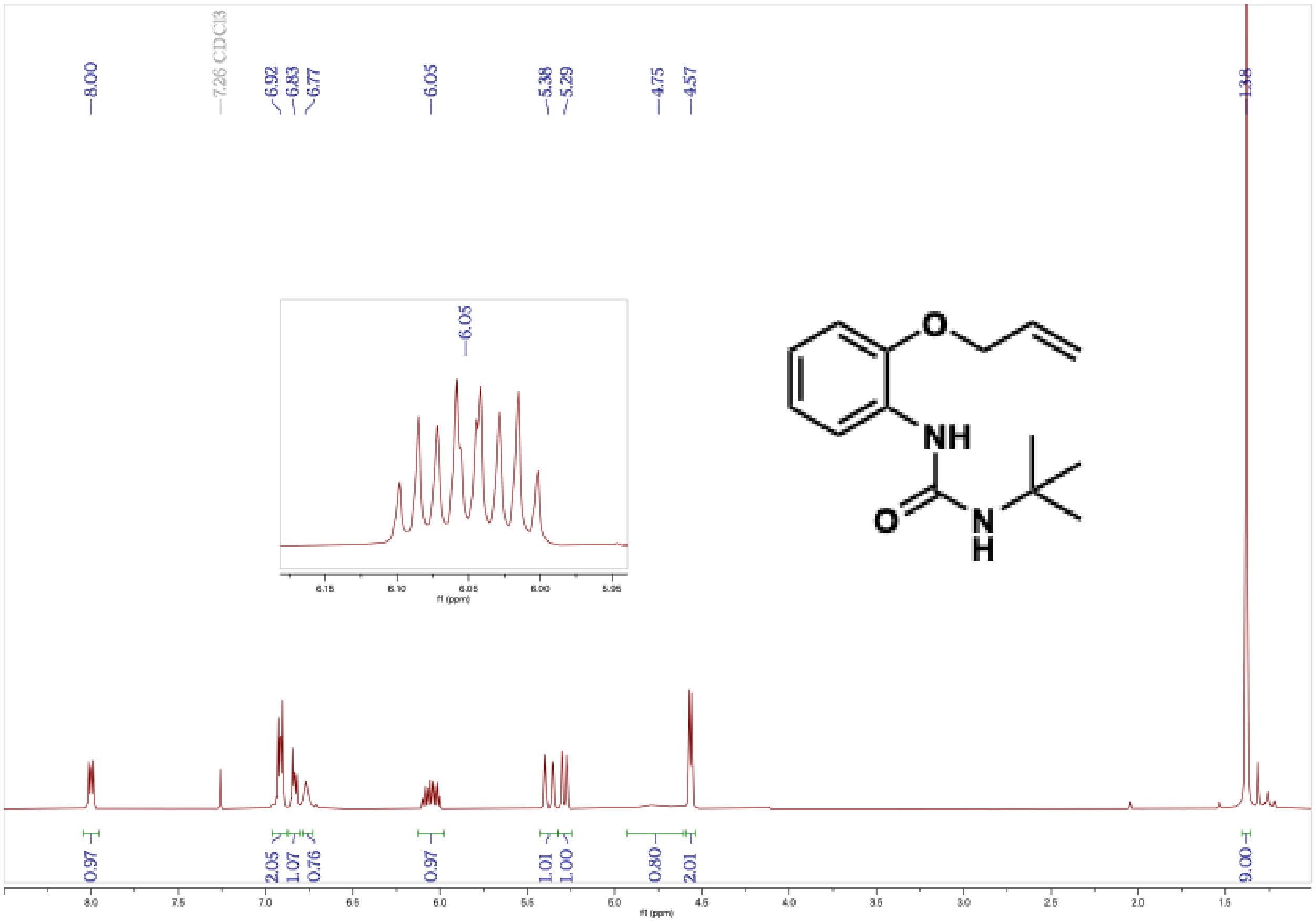

Figure S37. ^1H NMR spectrum (400 MHz, $CDCl_3$) of **7b**.

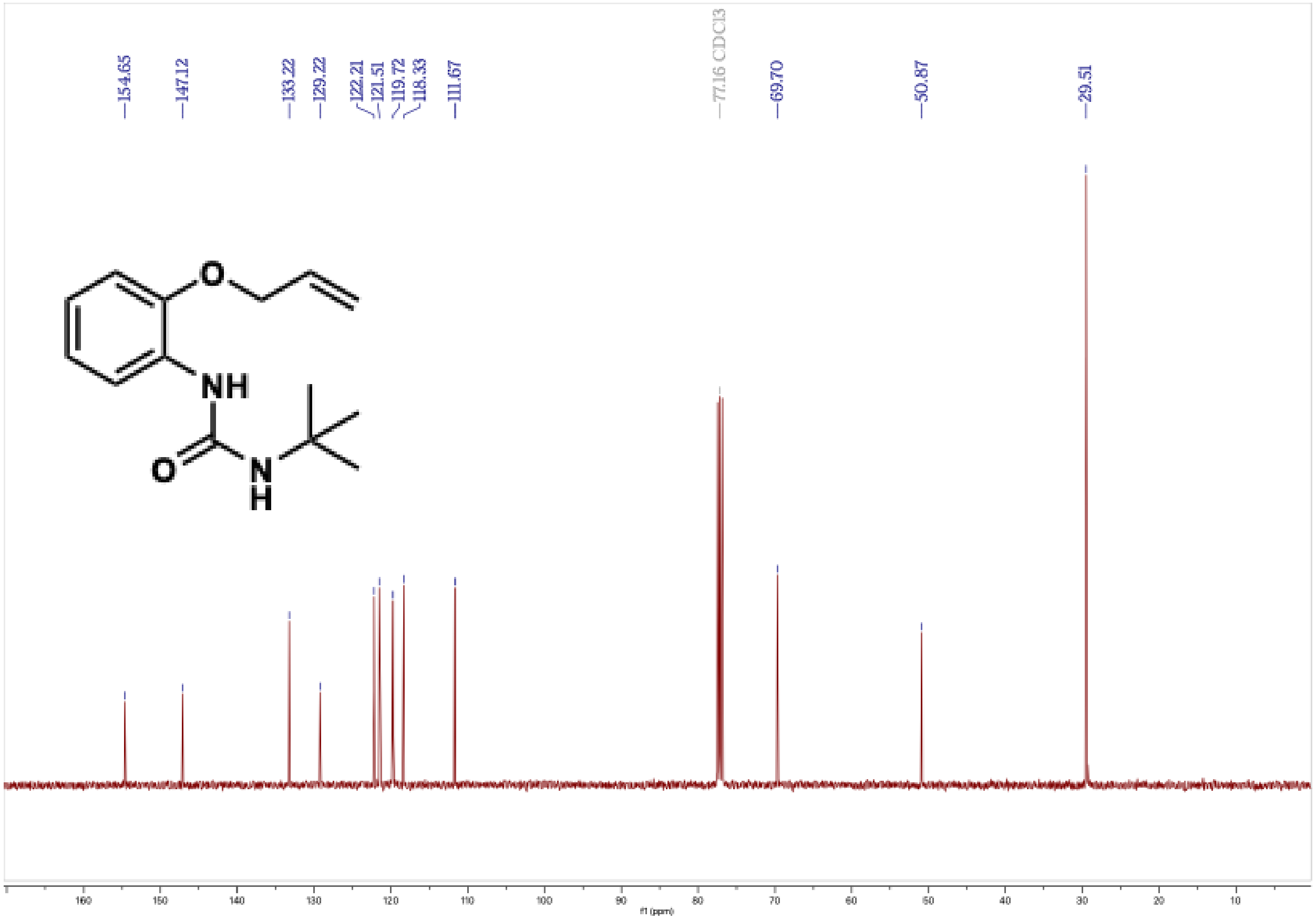

Figure S38. ^{13}C NMR spectrum (101 MHz, $CDCl_3$) of **7b**.

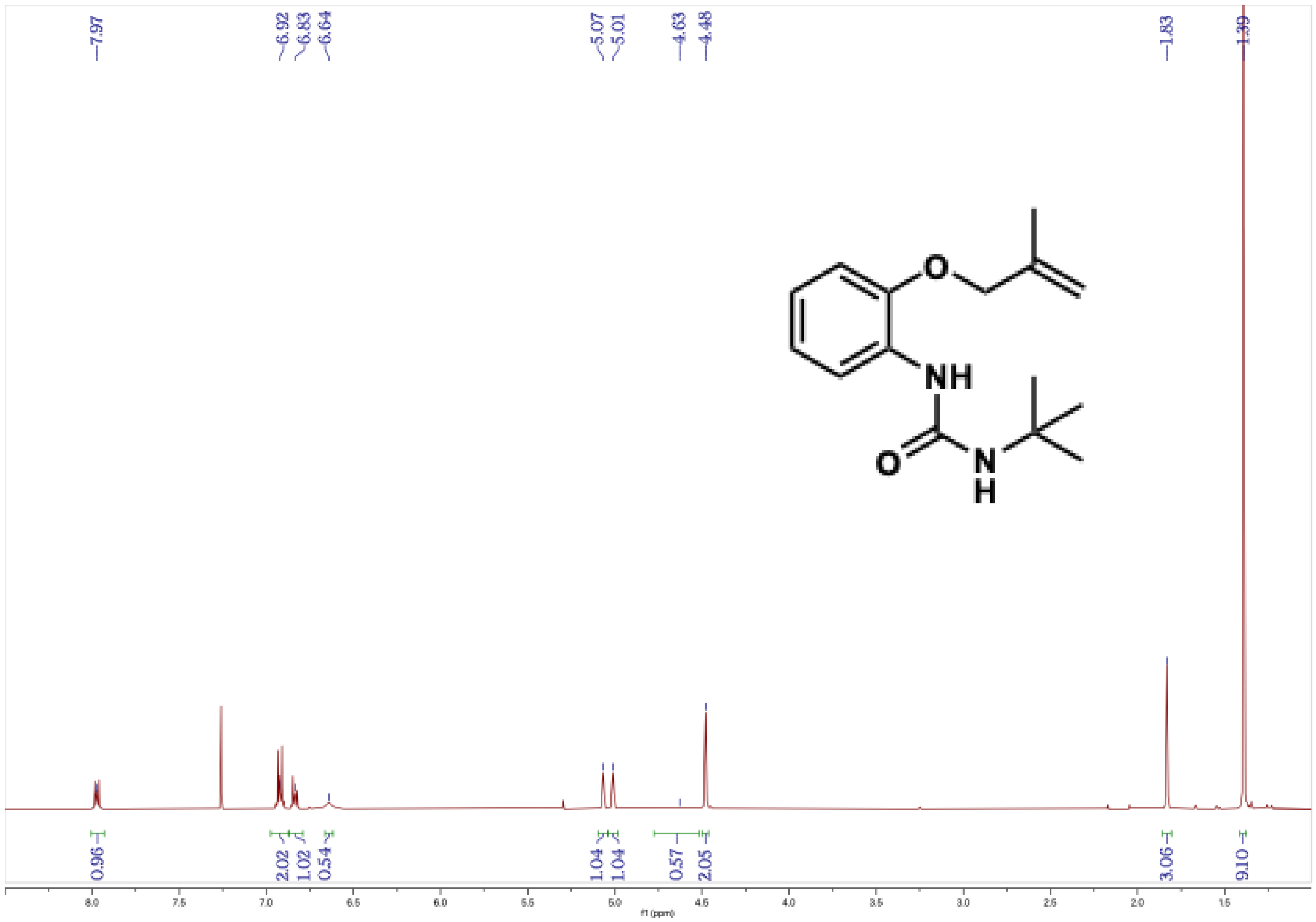

Figure S39. ^{1}H NMR spectrum (400 MHz, $CDCl_3$) of **7a**.

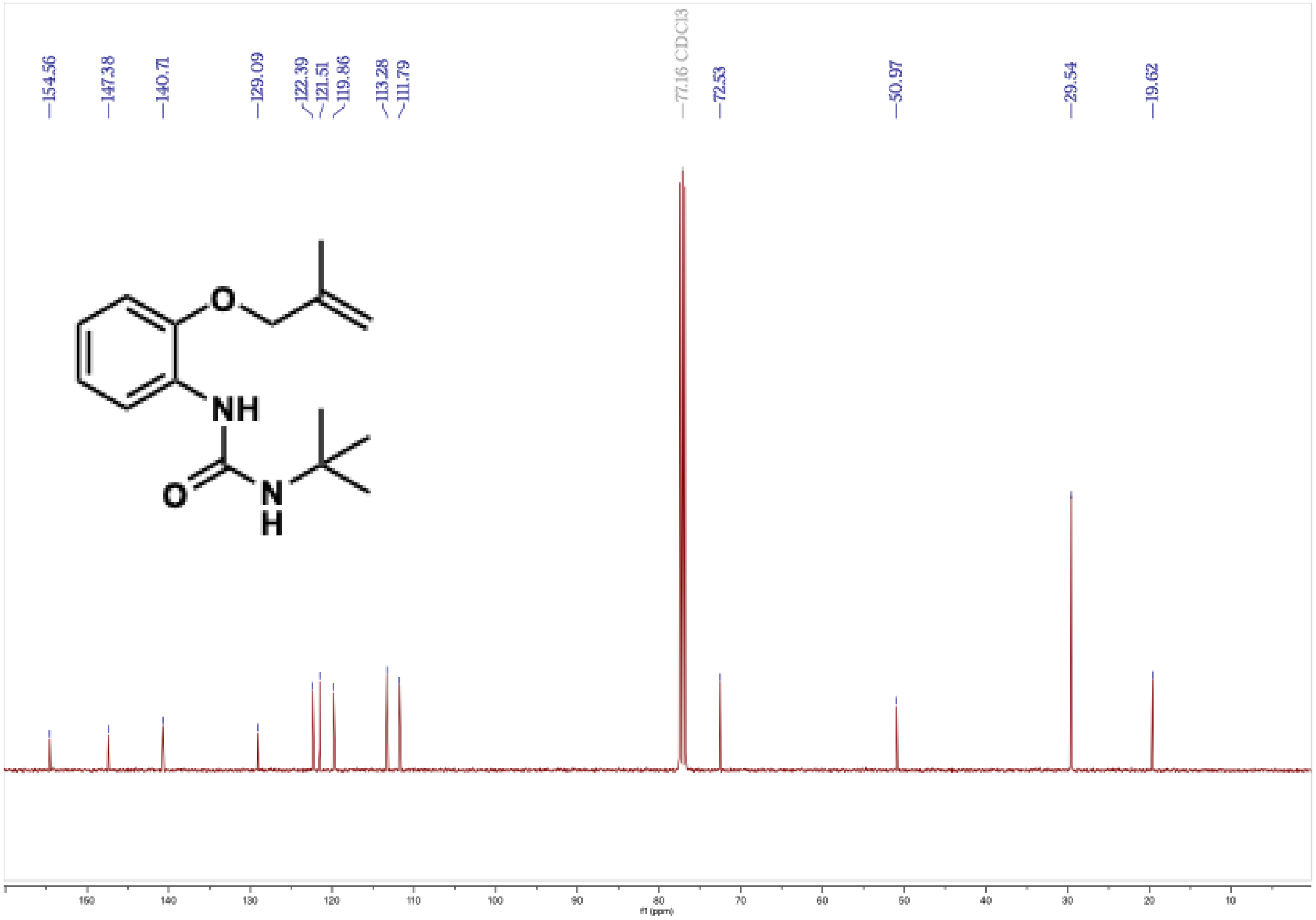

Figure S40. ^{13}C NMR spectrum (101 MHz, $CDCl_3$) of **7a**.

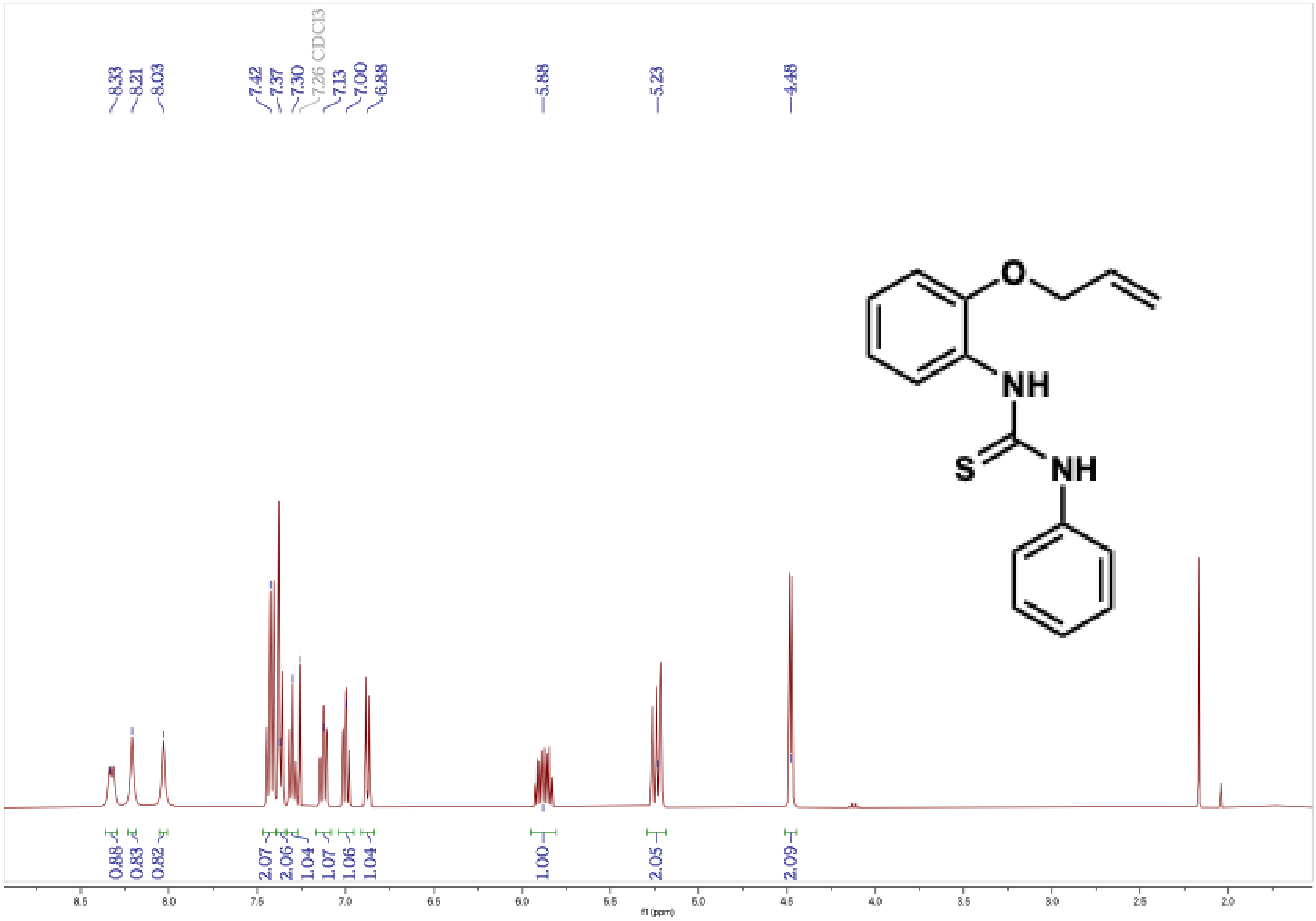

Figure S41. ^{1}H NMR spectrum (400 MHz, $CDCl_3$) of 1-(2-(allyloxy)phenyl)-3-phenylthiourea.

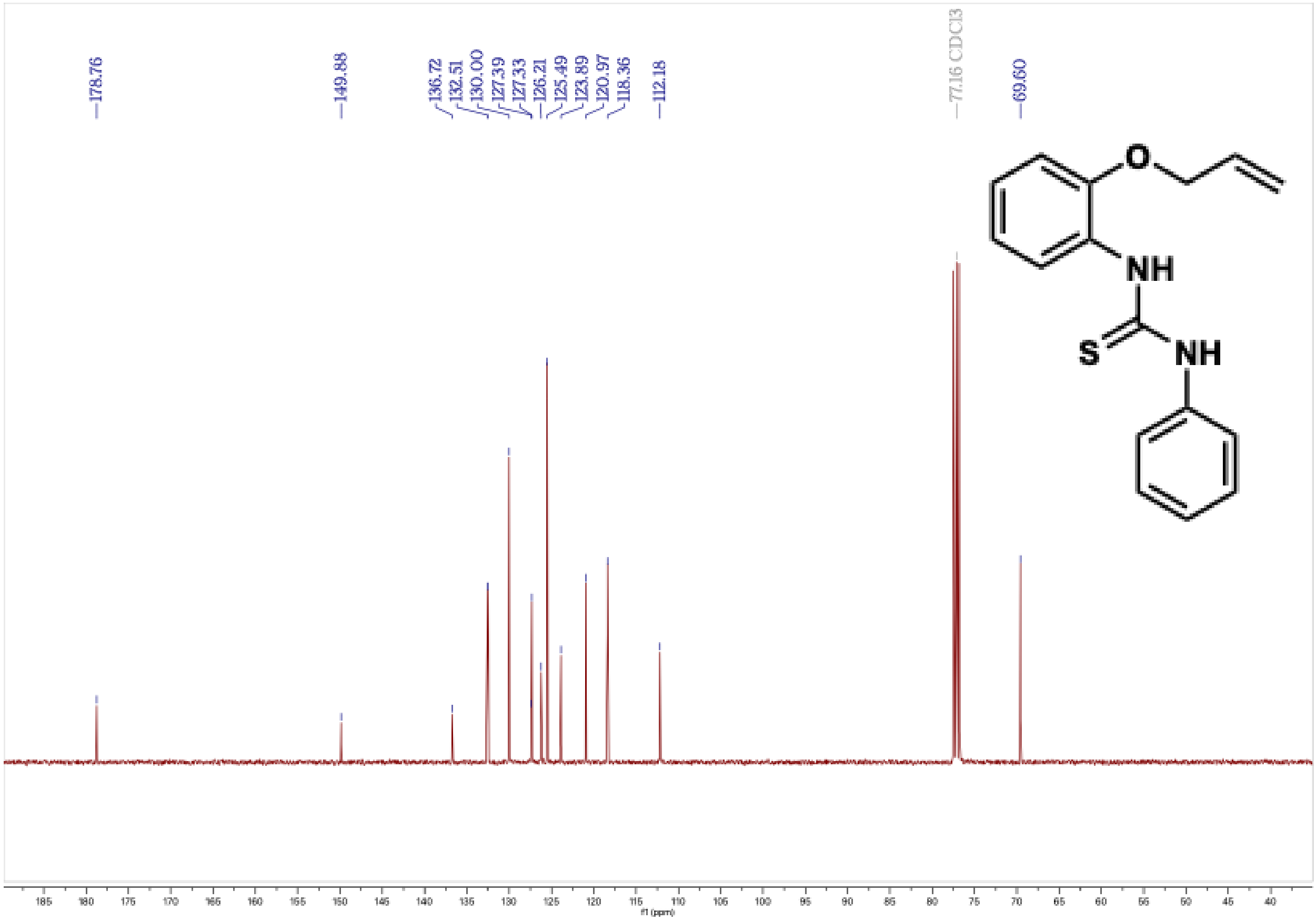

Figure S42. ^{13}C NMR spectrum (101 MHz, $CDCl_3$) of 1-(2-(allyloxy)phenyl)-3-phenylthiourea.

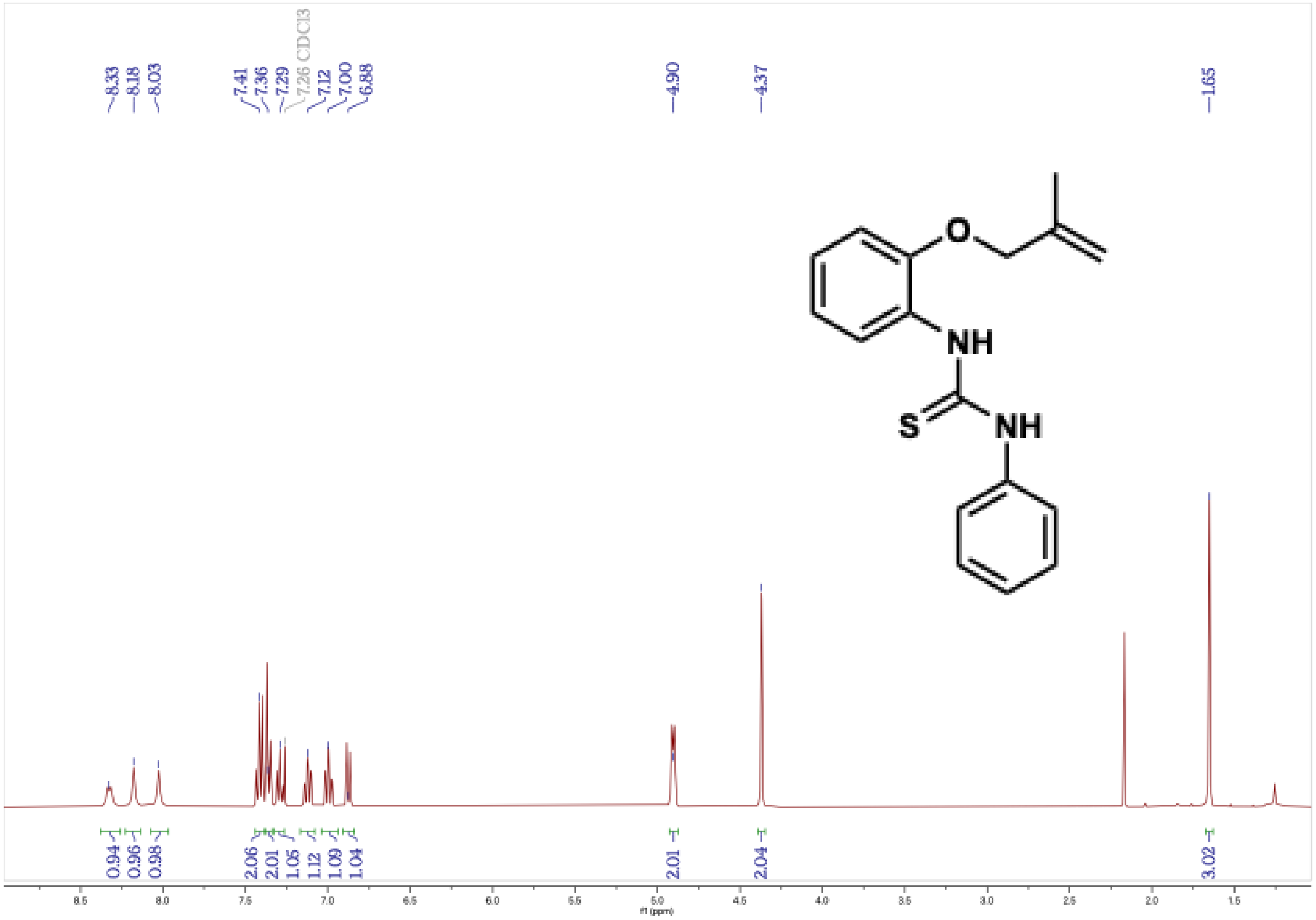

Figure S43. ^{1}H NMR spectrum (400 MHz, $CDCl_3$) 1-phenyl-3-(2-((2-methylallyl)oxy)phenyl)thiourea.

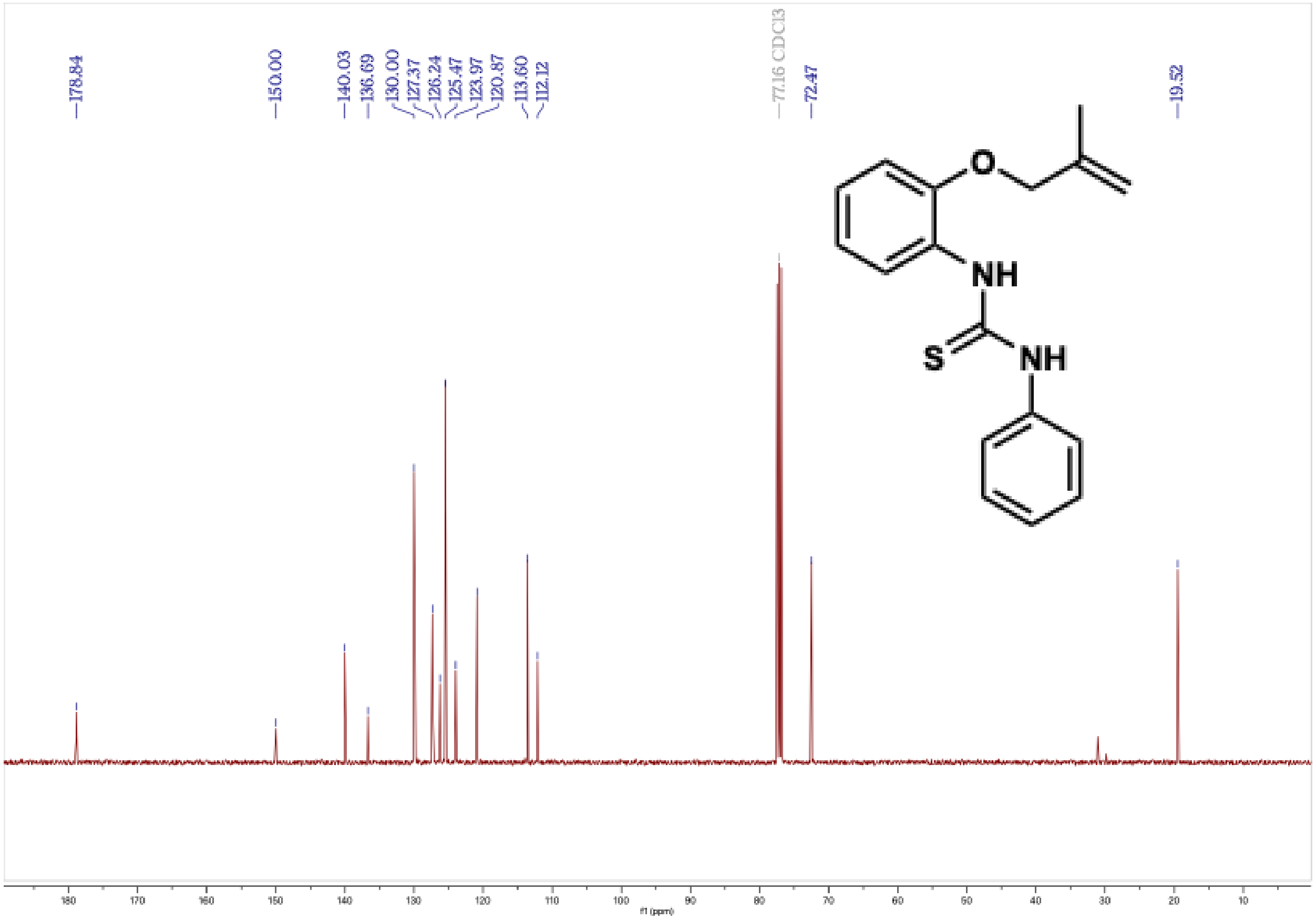

Figure S44. ^{13}C NMR spectrum (101 MHz, $CDCl_3$) 1-phenyl-3-(2-((2-methylallyl)oxy)phenyl)thiourea.

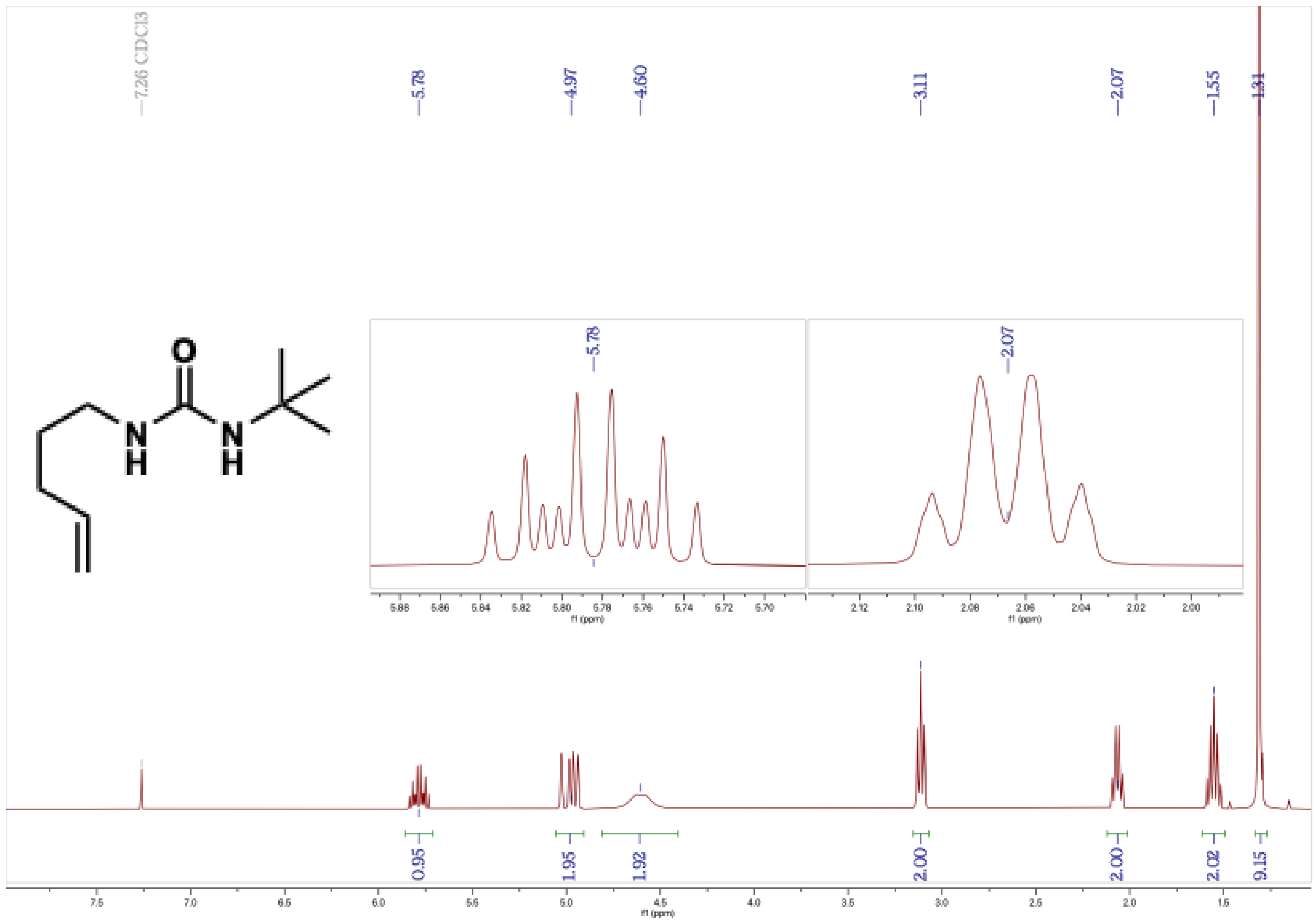

Figure S45. ^{1}H NMR spectrum (400 MHz, $CDCl_3$) of **12**.

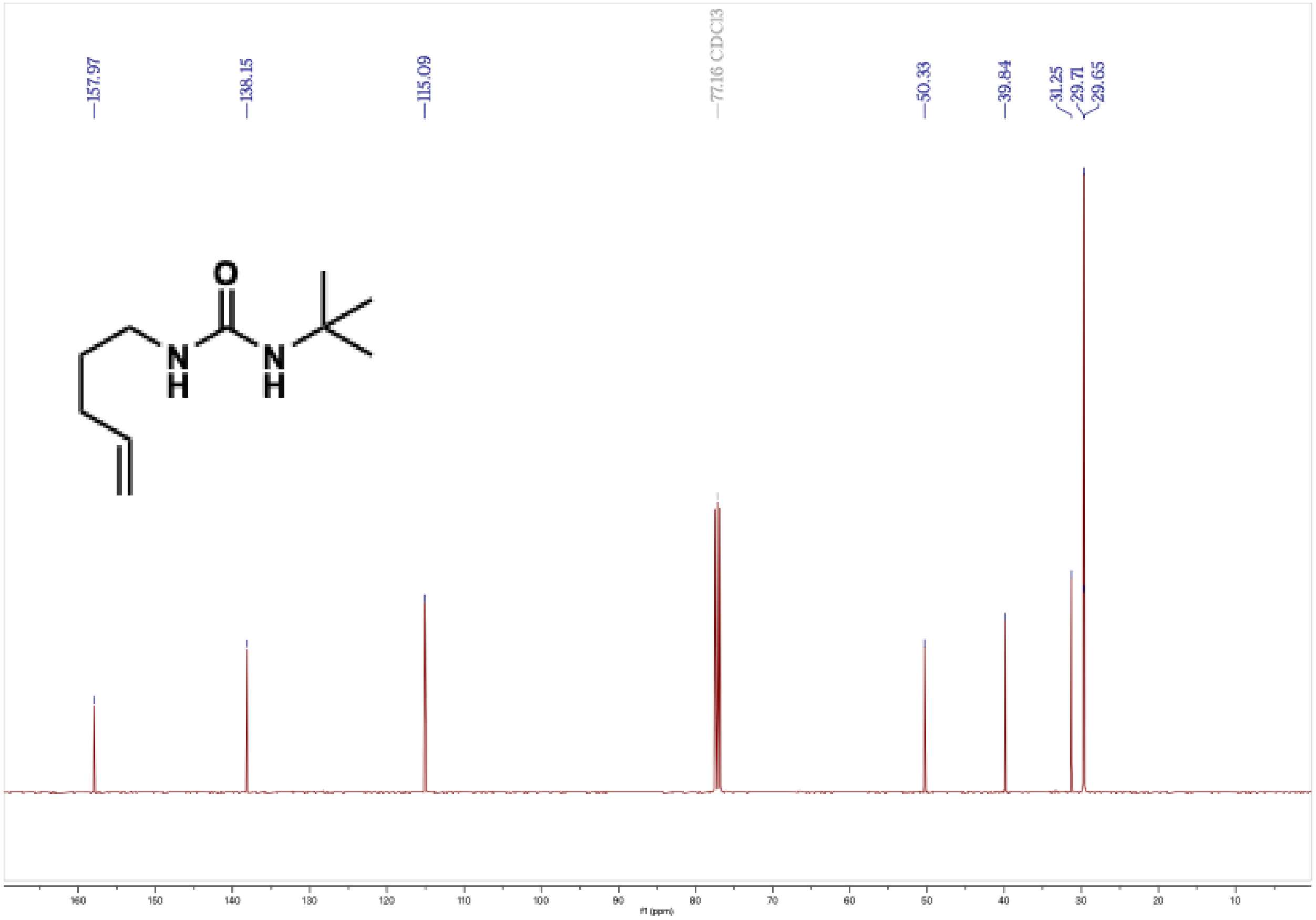

Figure S46. ^{13}C NMR spectrum (101 MHz, $CDCl_3$) of **12**.

Structure characterization – catalyst

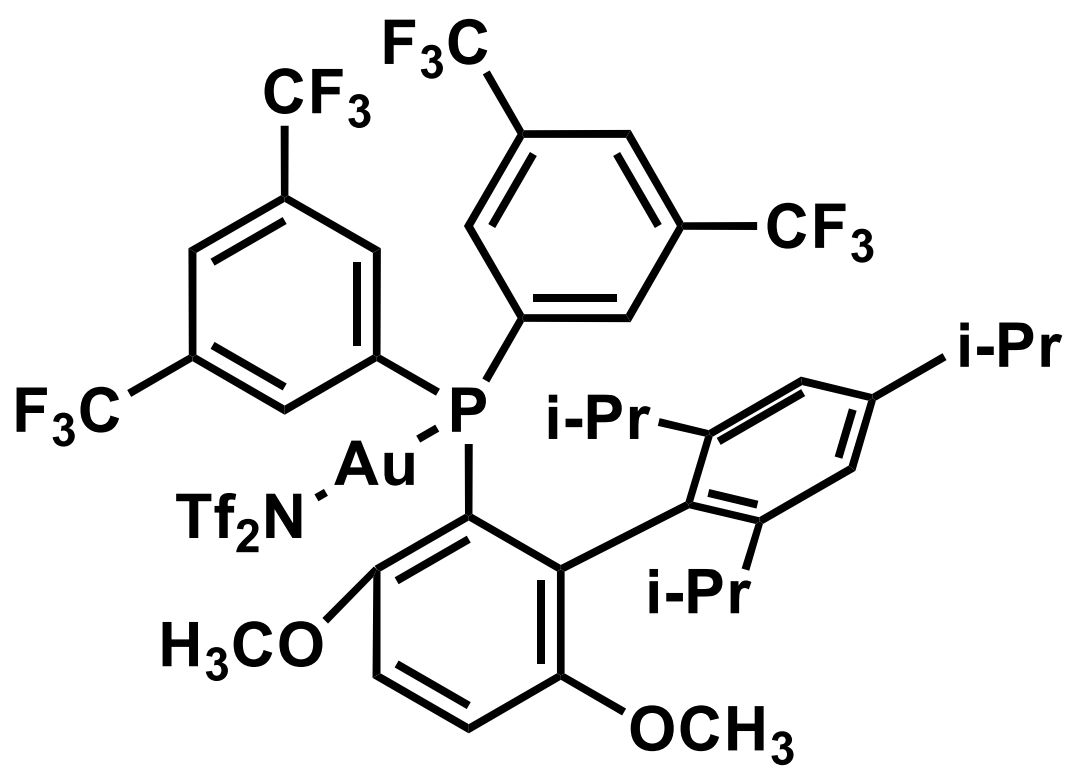

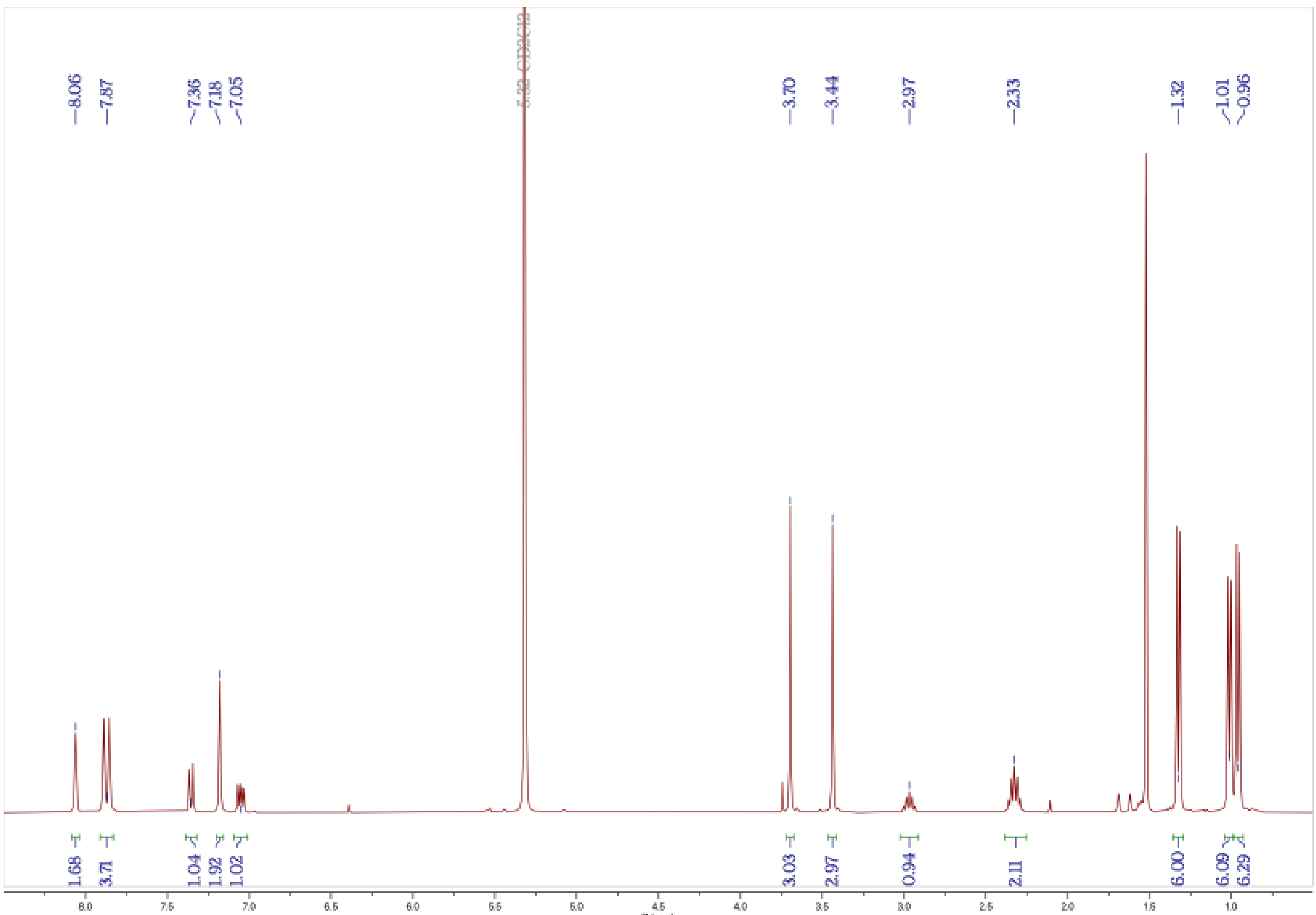

Figure S47. ^{1}H NMR spectrum (400 MHz, CD_2Cl_2) of JackiePhosAuNTf_2 (cat. **A)**.

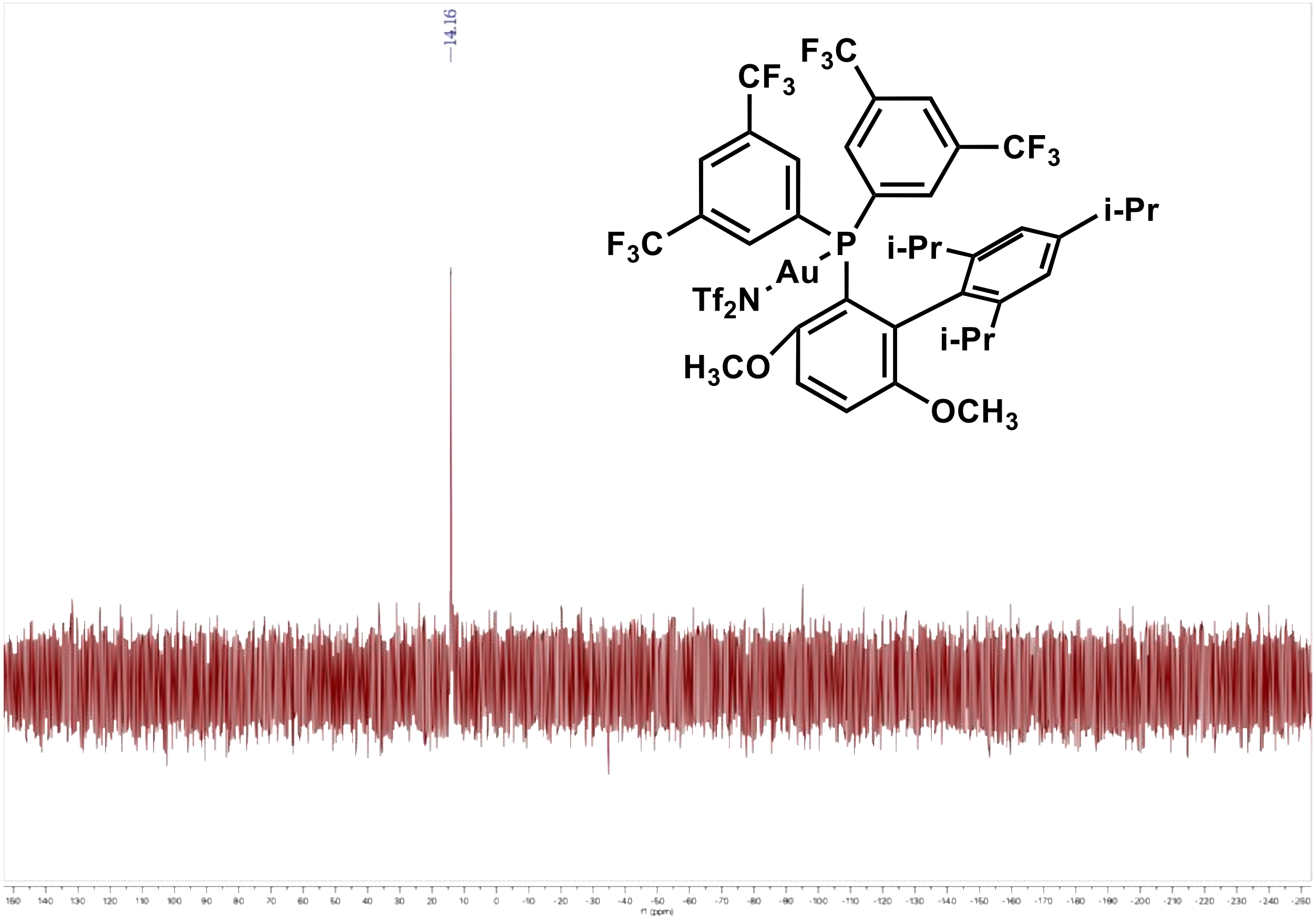

Figure S48. ^{31}P NMR spectrum (162 MHz) of JackiePhosAuNTf$_2$ (cat. **A)**.

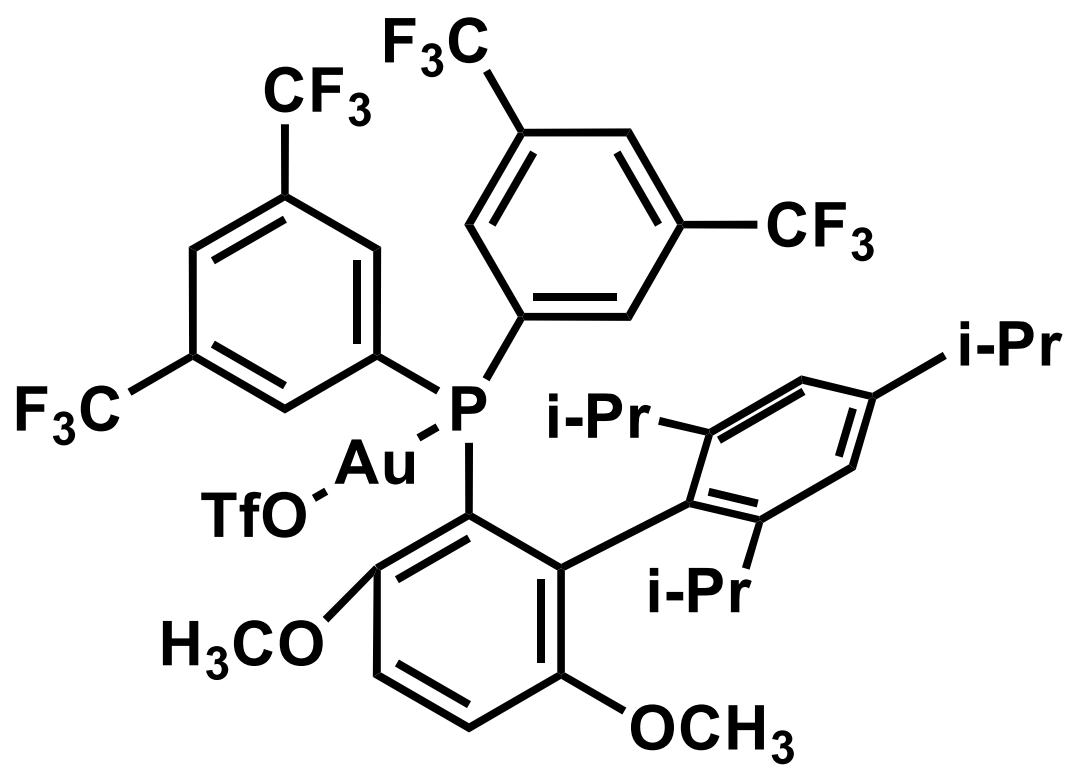

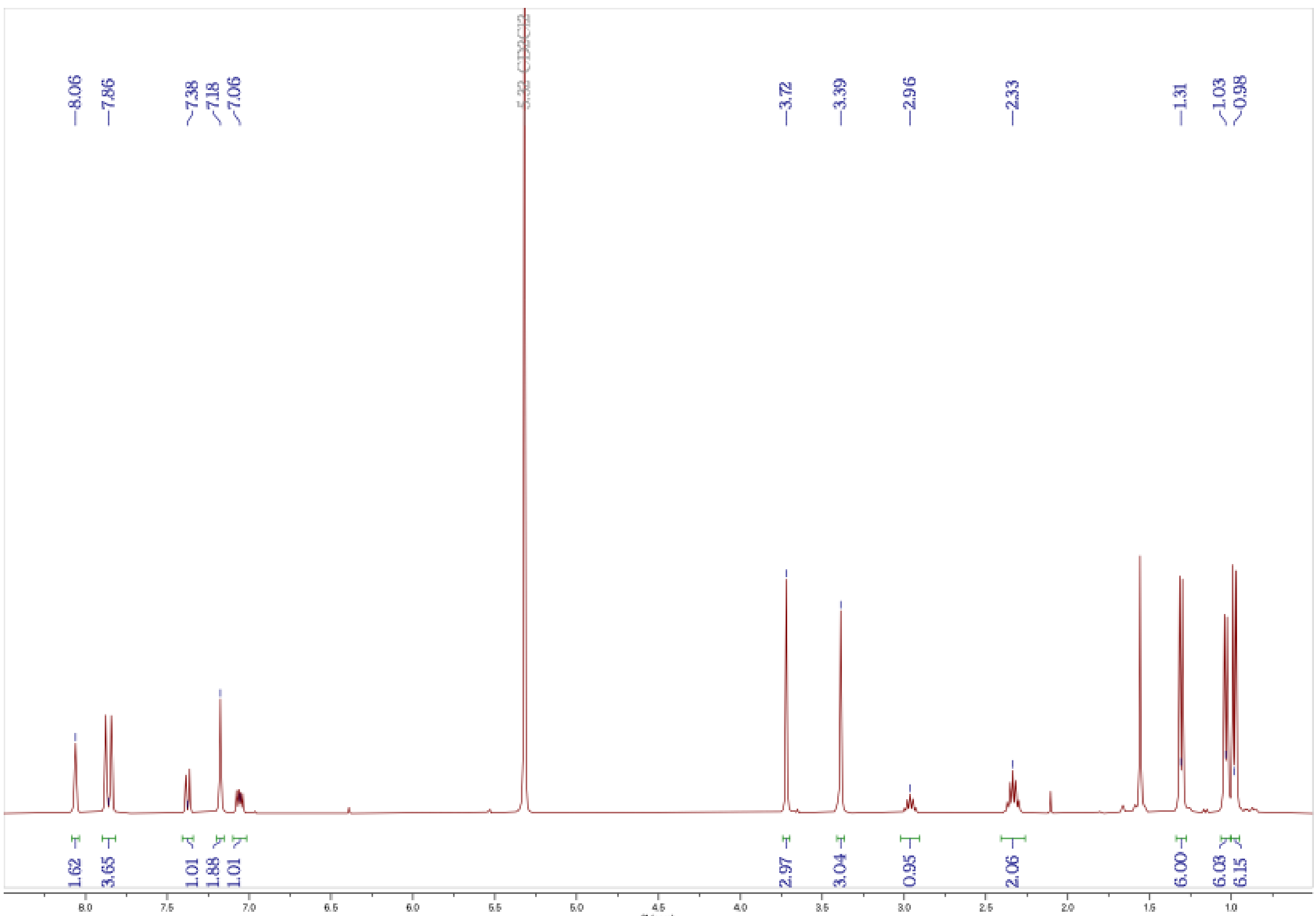

Figure S49. ^{1}H NMR spectrum (400 MHz, CD_2Cl_2) of JackiePhosAuOTf.

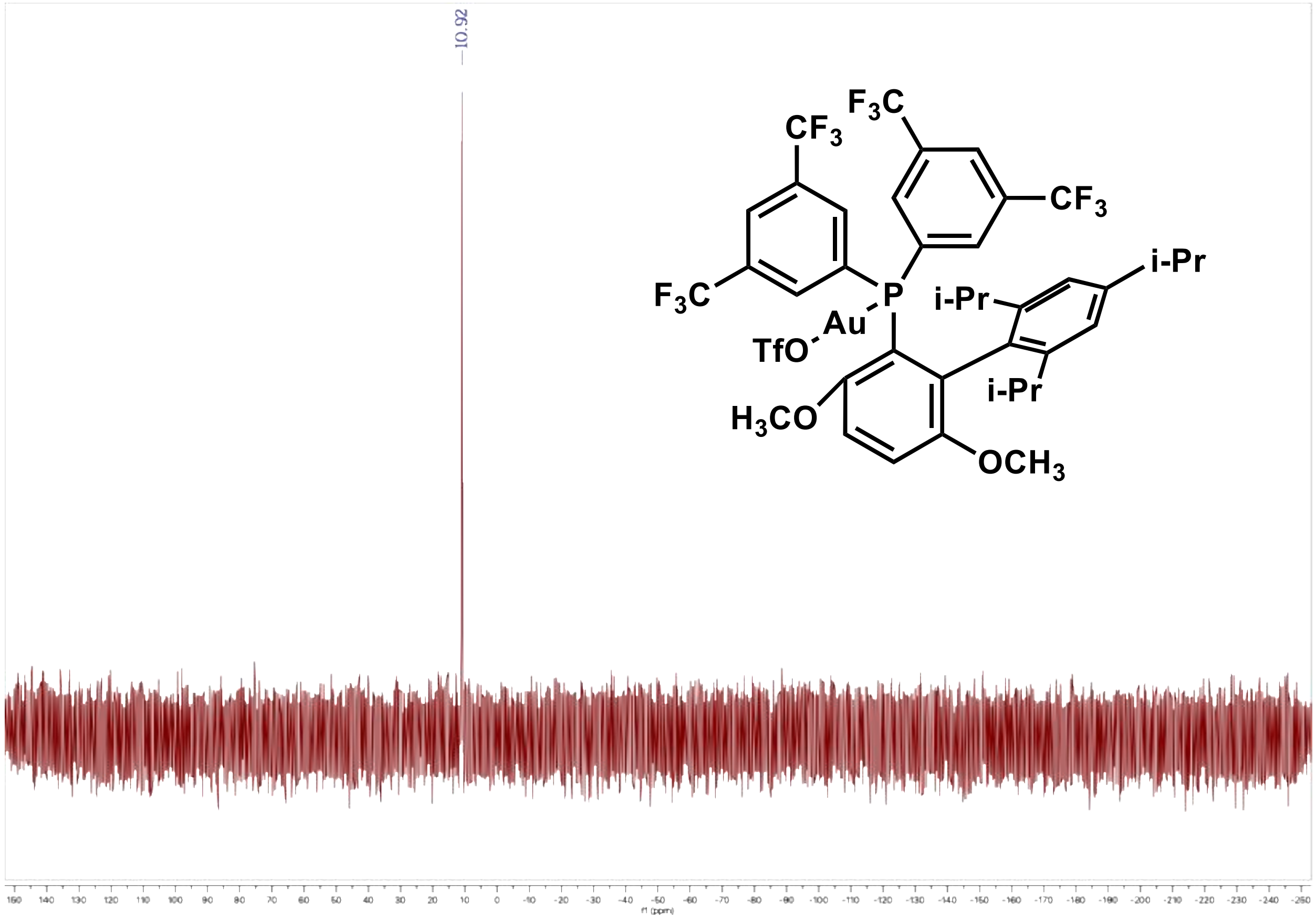

Figure S50. ^{31}P NMR spectrum (162 MHz) of JackiePhosAuOTf.

Product NMR

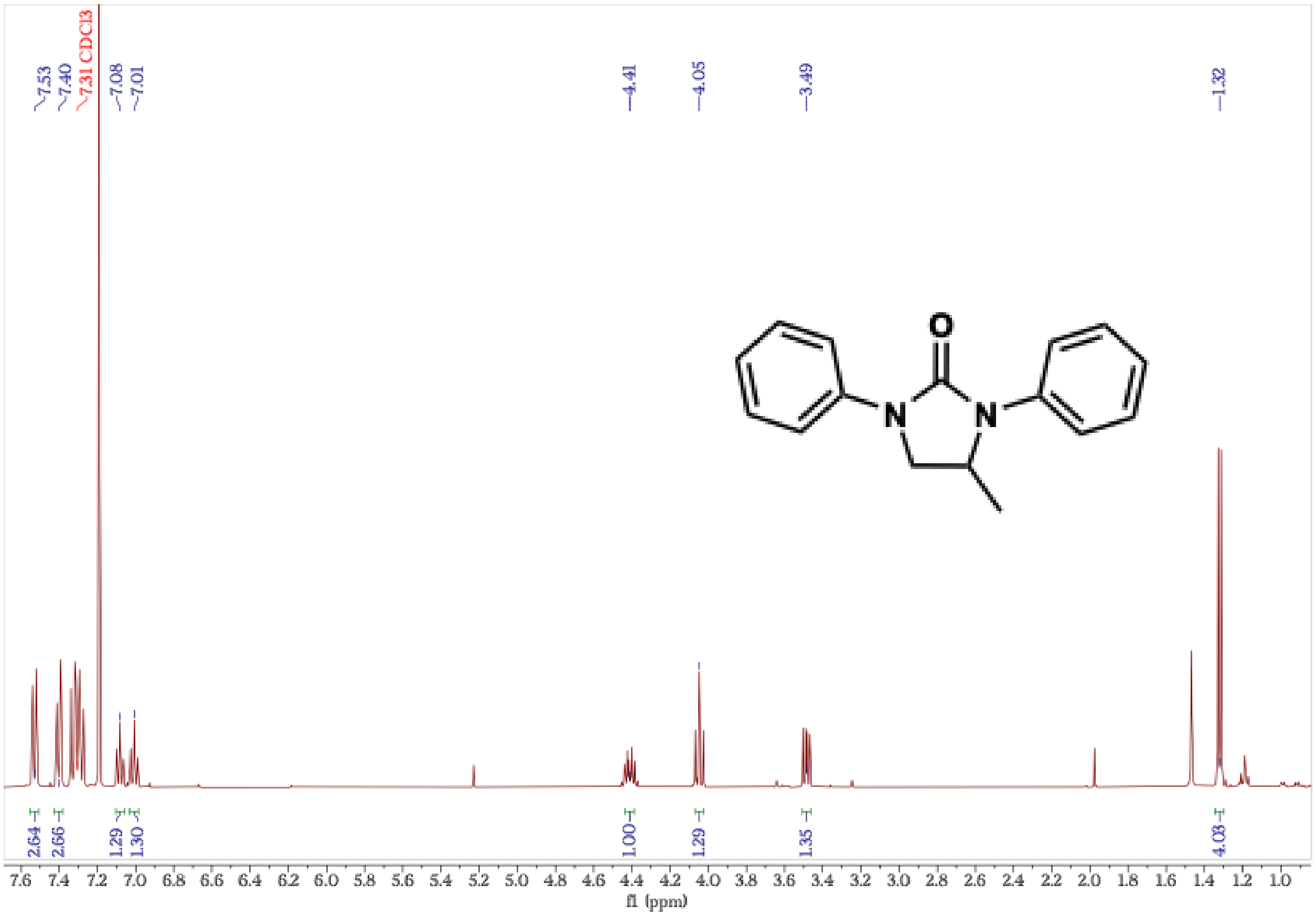

Figure S51. ^{1}H NMR spectrum (400 MHz, $CDCl_3$) of **3**.

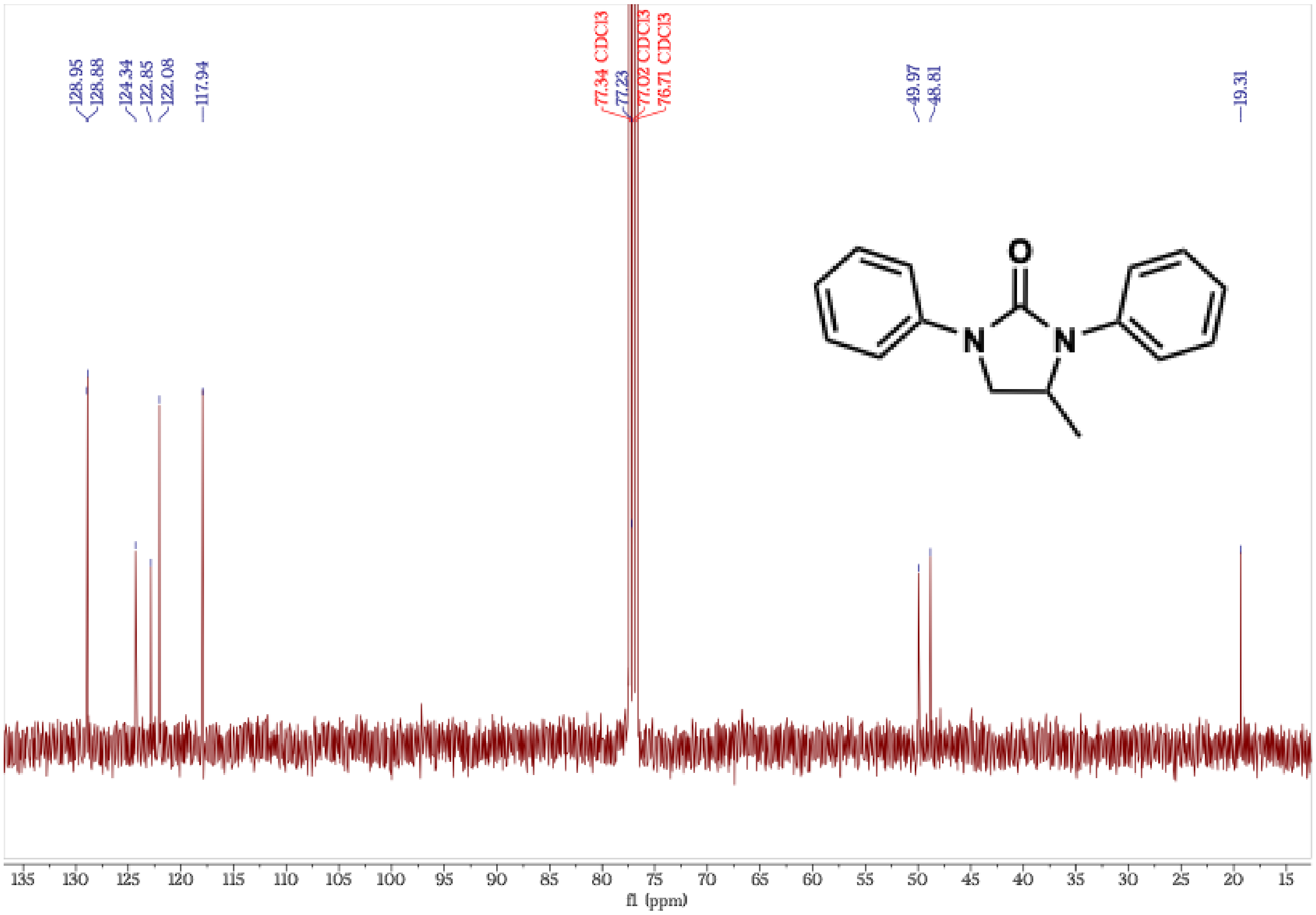

Figure S52. ^{13}C NMR spectrum (101 MHz, $CDCl_3$) of **3**.

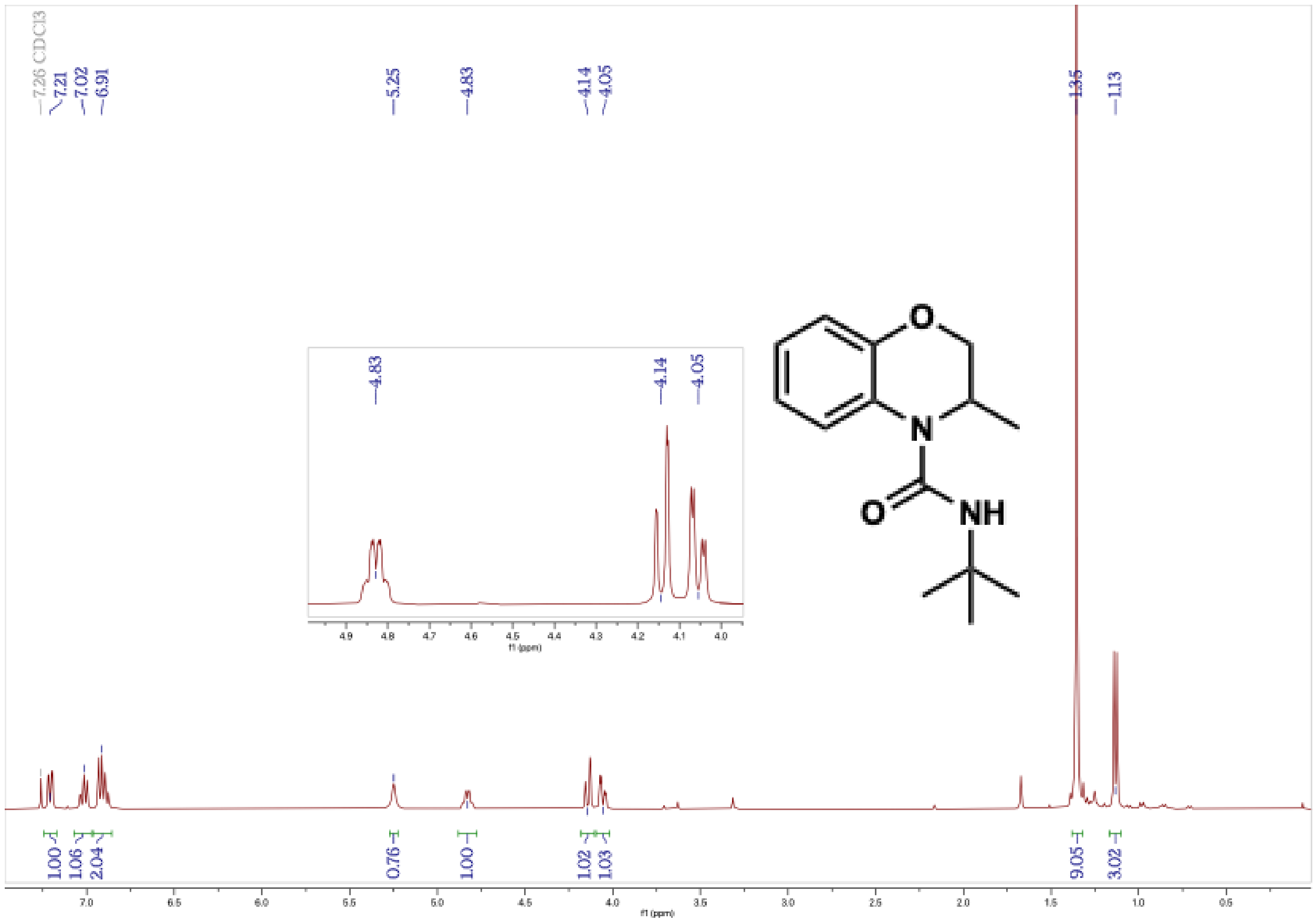

Figure S53. ^{1}H NMR spectrum (400 MHz, $CDCl_3$) of **9b**.

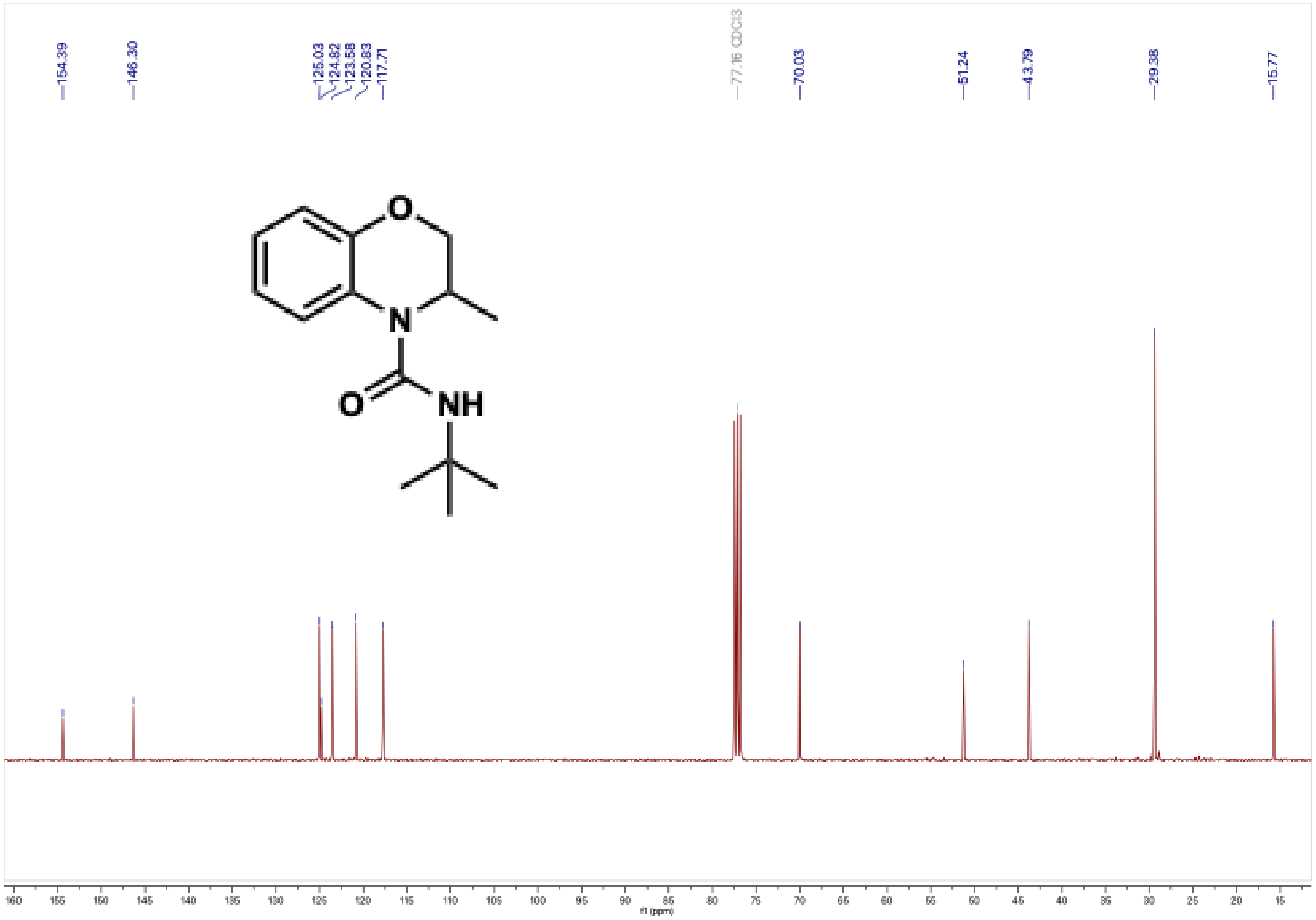

Figure S54. ^{13}C NMR spectrum (101 MHz, $CDCl_3$) of **9b**.

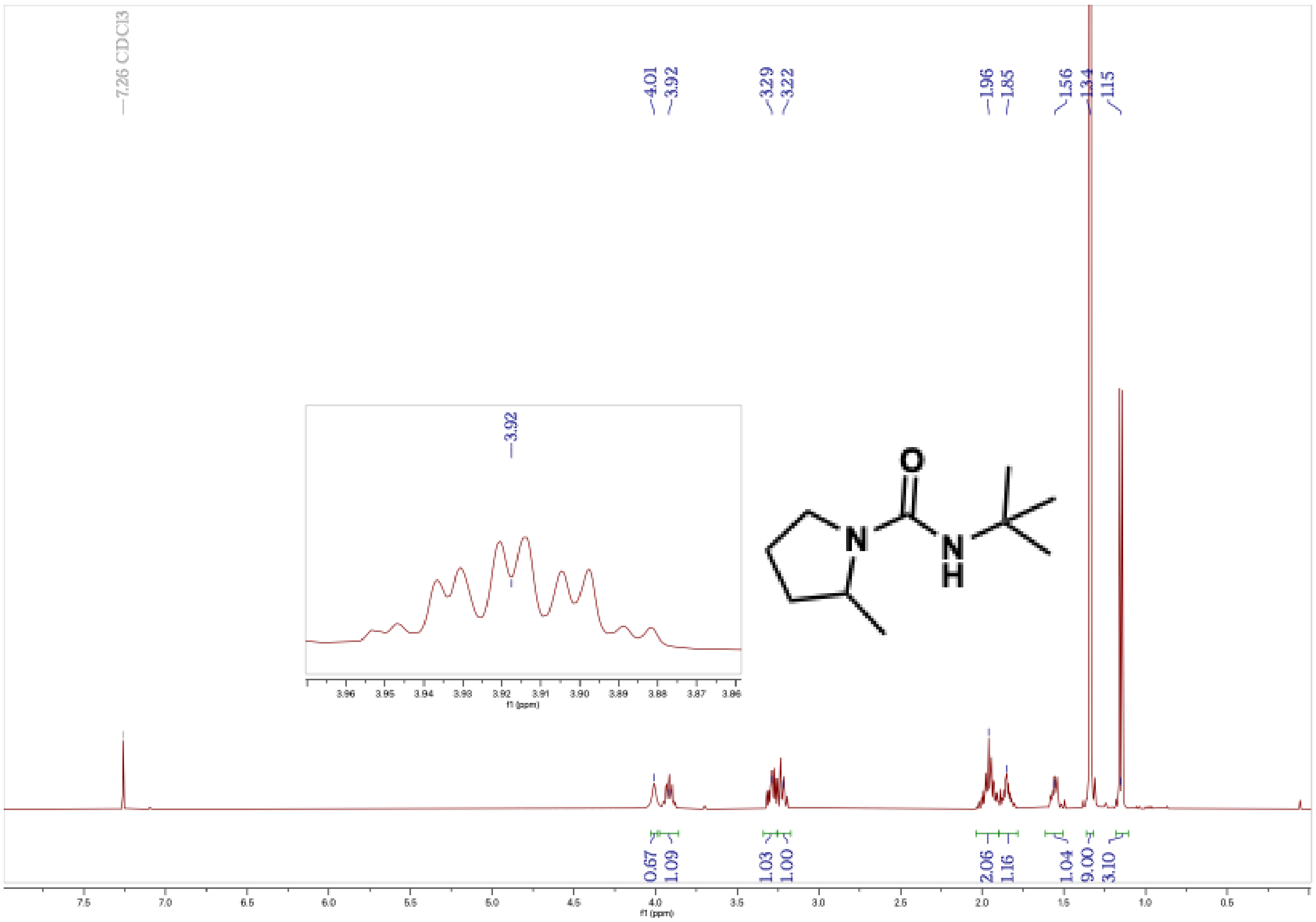

Figure S55. ^{1}H NMR spectrum (400 MHz, $CDCl_3$) of **13**.

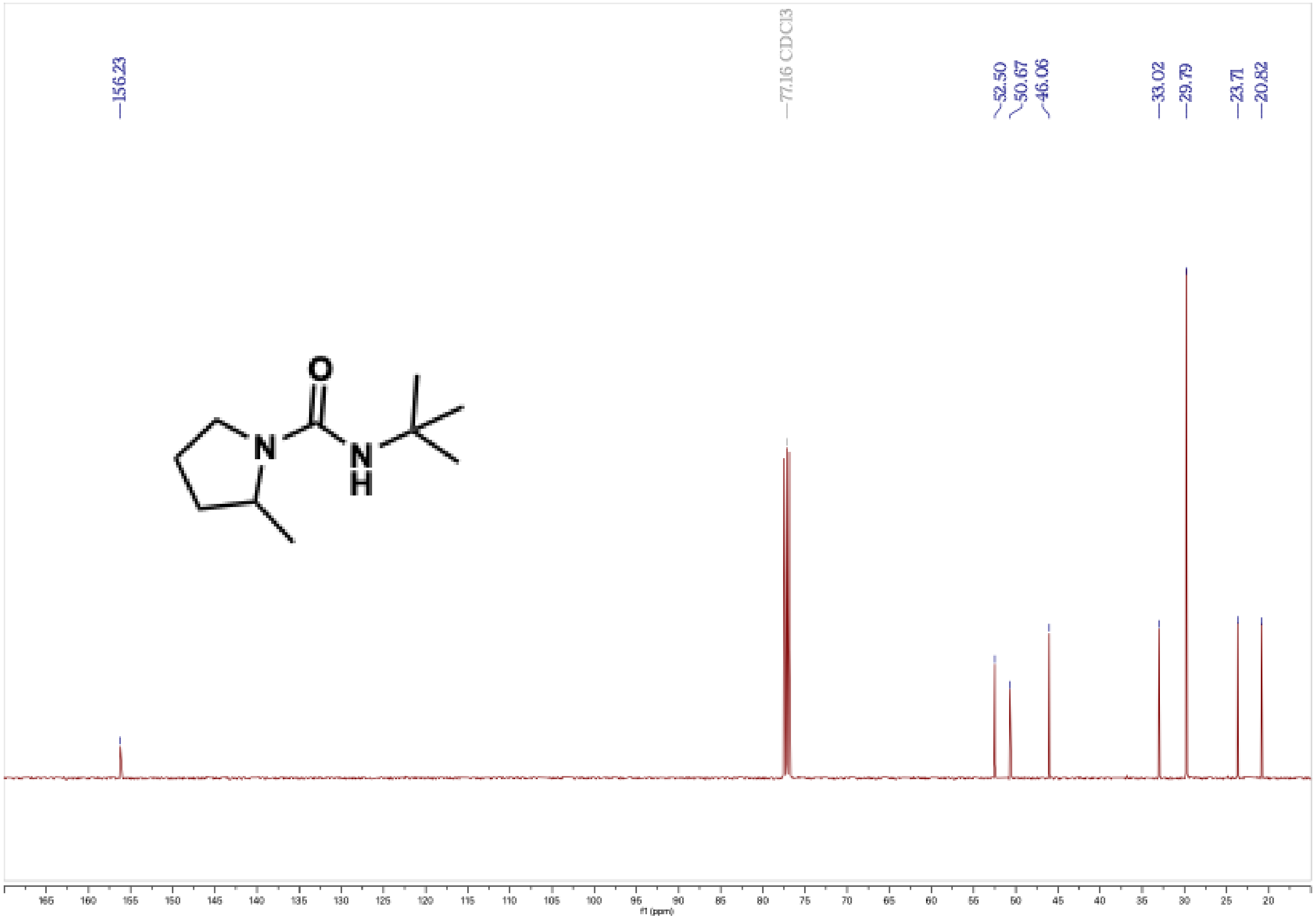

Figure S56. ^{13}C NMR spectrum (101 MHz, $CDCl_3$) of **13**.

References

S1. Sissengaliyeva, G.; Dénès, F.; Girbu, V.; Kulcitki, V.; Hofstetter, E.; Renaud, P. *Adv. Synt. Catal.* **2023**, *365*, 2568–2576.

S2. Ho, S. Y.; Bettens, R. P. A.; Dakternieks, D.; Duthie, A.; Tiekink, E. R. T. *CrystEngComm* **2005**, *7*, 682–689.

S3. Sardarian, A. R.; Dindarloo Inaloo, I.; Zangiabadi, M. *New J. Chem.* **2019**, *43* (22), 8557–8565.

S4. N. Ahmed; S. Khatoon, *ChemistryOpen* **2018**, *7*, 576.

S5. Islam, M.; Kariuki, B.M.; Shafiq, Z.; Wirth, T.; Ahmed, N. Eur. J. Org. Chem., 2019: 1371-1376.

S6. Kim, Y. H.; Kim, D. B.; Youn, S. W. *J. Org. Chem.* **2022**, *87* (17), 11919–11924.

S7. Barabé, F.; Levesque, P.; Korobkov, I.; Barriault, L. *Org. Lett.* **2011**, *13* (20), 5580–5583.
